# Cetacean Welfare Risk and the Educational Integrity of Ecotourism: A Multi-Framework Assessment of Whale-Watching Practices in the New York Metropolitan Area

**DOI:** 10.3390/ani16131955

**Published:** 2026-06-24

**Authors:** Jie Sima, Lien-Siang Chou, Wei-Cheng Yang

**Affiliations:** 1 Institute of Ecology and Evolutionary Biology, National Taiwan University, Taipei 106216, Taiwan; sima.jie@gmail.com (J.S.); chouls@ntu.edu.tw (L.-S.C.); 2 School of Veterinary Medicine, National Taiwan University, Taipei 106216, Taiwan

**Keywords:** animal welfare, whale watching, ecotourism, cetaceans, vessel disturbance, conservation education, New York Bight

## Abstract

Whale watching is frequently presented as a benign form of wildlife interaction, yet its ethical and ecological acceptability depends on two conditions: vessel practices must minimize disturbance to free-ranging cetaceans, and tours must provide meaningful conservation-oriented education. This study assessed whale-watching operations in the New York City Metropolitan Area using three complementary frameworks: the Whale SENSE “On the Water” evaluation, the World Cetacean Alliance (WCA) Best Practice Guidance, and a Higher-Order Thinking Skills (HOTS) framework for interpretation. Eight trips representing the active full-time commercial sector in the study area were observed between May and November 2022. The results have revealed that certified operators generally performed better than uncertified operators, but the difference was not large enough to demonstrate that certification alone ensured welfare-protective practice. Educational content was often present but shallow, with limited discussion of cetacean threats, conservation measures, and legal protections, while higher-order engagement and multilingual accessibility were notably weak. Vessel behavior showed a similar pattern: certified operators achieved higher average scores, yet close approaches, inconsistent adherence to conservative speed and maneuvering guidance, and occasional unacceptable practices were still recorded. Overall, some operations still expose whales to avoidable disturbance and fail to meet the educational standards that give ecotourism its conservation value. Responsible whale watching should therefore be evaluated not only by whether vessels find whales and satisfy tourists, but also by whether operators demonstrably protect animal welfare and cultivate informed conservation attitudes. As such, this study offers a regionally novel benchmark for future comparative research, management evaluation, and the development of more responsible cetacean ecotourism.

## Simple Summary

Whale watching has been widely framed as ecotourism and as a non-extractive use of whale populations, potentially raising cetacean conservation awareness. This claim holds true only if vessels minimize disturbance and if onboard interpretation genuinely promotes conservation. In the New York metropolitan area, whale watching has expanded in a busy urban marine environment where free-ranging cetaceans already face multiple human pressures. Few published studies assess whale-watching practices in this region, making it hard to judge whether operators meet expected cetacean welfare risk and educational standards. This study provides a baseline assessment of whale-watching operators in the region using local and international evaluation frameworks, together with an assessment of higher-order educational engagement. Study results revealed that certified operators generally performed better than uncertified operators, but certification did not guarantee vessel conduct that consistently protected cetaceans or interpretation that fulfilled the educational promise of ecotourism. Operators often made close approaches to whales and showed weak compliance with conservative vessel-handling standards. Combined with shallow conservation messaging and poor multilingual access, these practices make parts of the sector look more like a wildlife spectacle than responsible ecotourism. Poor vessel behavior in this area may increase the risk of disturbance, and weak educational programs are unlikely to inspire conservation awareness in tourists. Therefore, the whale-watching industry in the New York City metropolitan area would benefit from more efficient enforcement of existing laws and guidelines, and operators should create more robust interpretive programming to support conservation awareness.

## 1. Introduction

Following the International Whaling Commission’s moratorium on commercial whaling, whale watching spread worldwide as a non-lethal alternative and is now promoted as a form of ecotourism [1,2]. However, ecotourism is not defined merely by the presence of wildlife or tourist enjoyment. Its ethical credibility depends on how well tourism minimizes harm and contributes to the conservation of the species and habitats it relies on [3,4,5,6]. For cetaceans, this implies that assessments of whale-watching should go beyond visitor satisfaction or profit to include how responsibly vessels operate and how effectively interpretation fosters conservation.

Animal welfare is relevant even in non-captive, non-invasive wildlife tourism contexts. Vessel interactions can change how free-ranging cetaceans move, breathe, communicate, or use space, and repeated disturbance may still cause energetic or acoustic stress even without visible injury [7,8,9,10,11,12,13,14,15,16,17,18,19,20]. Whale-watching guidelines are not just about navigation: they aim to reduce disturbance and protect animal welfare [7,8,9,10,11,12,13,14,15,16,17,18,21]. When vessels approach too closely, maneuver aggressively, restrict travel paths, or remain with animals in ways that intensify pressure, the encounter may shift from observation to disturbance. Poor vessel behavior is consequently an animal-welfare issue as well as a regulatory one [3,7,8,9,10,11,12,13,14,15,16,17,18,19,20].

The educational dimension of whale watching is equally important. Whale watching is often justified socially because it is assumed to increase public knowledge, build empathy, and strengthen conservation support [4,5,6,22,23,24,25,26,27,28,29]. When interpretation is shallow or out of touch with real conservation issues, ecotourism loses the moral weight often used to justify it [3,4,5,6,22,23,24,25,26,27,28,29]. Research shows that the quality of guiding and visitor engagement can shape how satisfied people feel and how strongly they support conservation afterwards [4,5,6,23,25,26,27,28,29]. However, if educational components are weak, the animals are likely to bear the burden of tourism without receiving the conservation benefit that ecotourism claims to provide. Weak education programs are not just service flaws. In fact, they represent a fundamental failure in conservation-oriented tourism.

**Figure 1:**
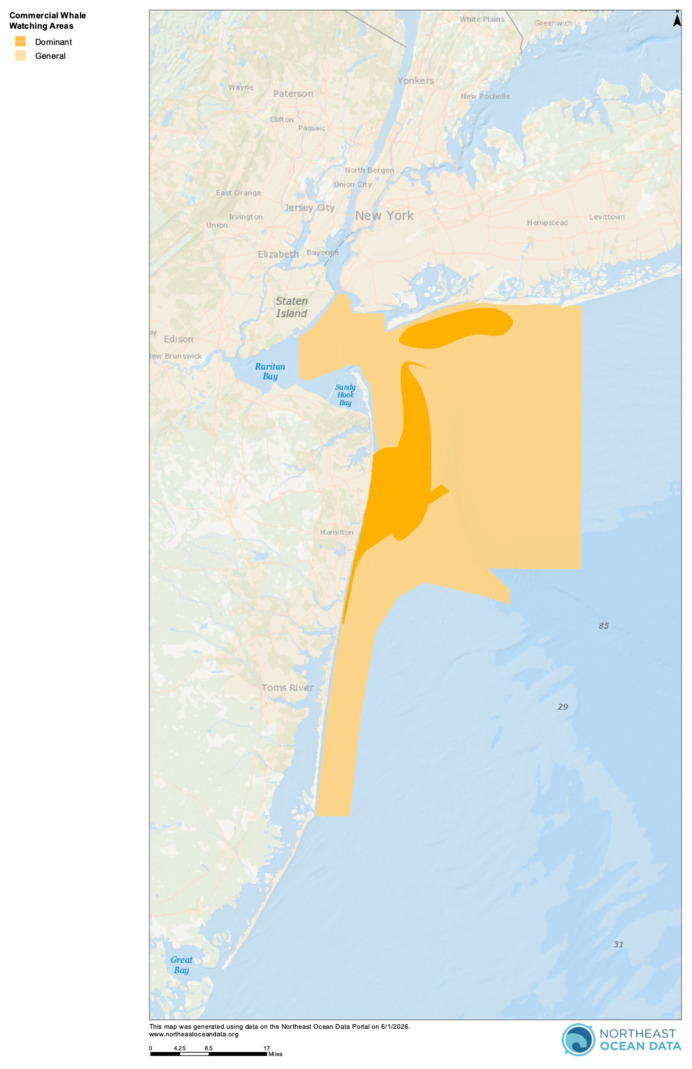
Commercial whale watching areas in the New York Bight region. The map shows dominant (dark orange) and general (light orange) commercial whale watching areas extending from New York Harbor and Raritan Bay southward along the New Jersey coast, based on data from the Northeast Ocean Data Portal (accessed on 1 June 2026).

The New York Bight provides a particularly important case study. The region hosts regular sightings of baleen whales and dolphins, but their habitats overlap with shipping, fishing, recreation, and a growing whale-watching industry [30] (Figure 1). From a conservation perspective, several whale species, such as North Atlantic right whales (*Eubalaena glacialis*) and fin whales (*Balaenoptera physalus*), in the broader region face elevated extinction risk globally [31]. Despite this, the New York metropolitan area has received relatively little published evaluation of whale-watching practices, especially from the combined perspective of vessel conduct and educational quality [30,32]. This study asks two questions: (1) Do vessel practices minimize disturbance to free-ranging cetaceans? and (2) Does onboard interpretation achieve the conservation and educational goals of ecotourism? Using Whale SENSE criteria, the World Cetacean Alliance (WCA) Best Practice Guidance, and a Higher-Order Thinking Skills (HOTS) framework, the study evaluates whether certified and uncertified operators differ in ways that matter for animal welfare and for the educational integrity of urban whale watching [21,24,32,33].

## 2. Materials and Methods

### 2.1. Study Area and Operator Identification

The study focused on the New York City Metropolitan Area, encompassing the New York–New Jersey Bight and the principal whale-watching departure points serving this region. Field observations were conducted between May and November 2022, corresponding to the main whale-watching season, when both cetacean encounters and tourism activity are most common. Commercial operators were identified through systematic internet searches and a review of publicly available business information. The candidate operators were screened using three inclusion criteria: active commercial operations during the study period, regular scheduling of cetacean-focused trips, and publicly advertised whale-watching services on official websites. Operators offering only private charters, those without active programs, and those advertising whale watching without current operations were excluded. The final selection yielded eight active full-time commercial sectors in the study area.

### 2.2. Observational Design

A total of eight whale-watching trips were observed between May and November 2022. Four observed operators (U1–U4) were Whale SENSE certified, and four (U5–U8) were not. Two trained observers attended trips and completed standardized scoring sheets. To reduce overt behavior modification, operators were not informed that compliance and interpretive quality were the specific focus of evaluation during the observation period. In analytical terms, the trip, rather than the individual observer, was treated as the unit of observation. When one observer’s form was incomplete, the more complete paired record was used for the relevant criterion.

This study did not directly measure physiological stress, fine-scale behavioral budgets, or individual cetacean responses. Instead, it assessed welfare-relevant risk indirectly through vessel practices that existing local and international guidance explicitly seeks to regulate, such as approach distance, speed, direction of approach, path restriction, repeated targeting, and other unacceptable maneuvers. The educational component was treated as an indicator of whether whale watching functioned as conservation-oriented ecotourism rather than a wildlife spectacle alone.

### 2.3. Evaluation Frameworks and Scoring

Three complementary frameworks were applied (details provided in the Supplementary File S1). First, the Whale SENSE “On the Water” evaluation was used because it represents the principal regional certification benchmark [21]. It includes both educational and vessel-behavior criteria. Educational items were scored from 1 to 3, where 1 indicated disagreement or absence, 2 indicated limited mention or neutrality, and 3 indicated adequate coverage. Vessel-behavior items were scored from 1 to 5, where higher scores represented stronger adherence to Whale SENSE expectations.

Second, the World Cetacean Alliance (WCA) Global Best Practice Guidance was used to benchmark observed behavior against a broader international standard [33]. The WCA guidance ascribed a simplified 1–3 scale across both educational and vessel-behavior criteria, applied for positive criteria (1 = not met, 2 = partly met, 3 = met), while clearly unacceptable practices were recorded as absent (0) or present (1). This allowed comparison across frameworks while preserving the distinction between good practice and explicitly prohibited conduct.

Whale SENSE is used broadly across the USA and differs from the WCA evaluation, which is used internationally, in that it maintains shorter safety distances from cetaceans and provides a basic overview of acceptable vessel behavior, along with five criteria for narration. The WCA evaluation includes a more comprehensive list of vessel behavior criteria, including a critical list of unacceptable practices, which is missing from Whale SENSE, along with six criteria for narration. This combination was chosen in order to better understand how vessels comply with both local certification standards and international guidelines.

Third, a HOTS framework [34] was applied to interpretive programming to assess whether educational narration went beyond fact delivery. The HOTS criteria considered whether operators encouraged analysis, evaluation, synthesis, and interactive engagement. HOTS education criteria were assessed based on the frequency of HOTS questions included in the narration schedule and the frequency with which tourists were encouraged to interact with staff or each other. Items were scored from 1 to 3, where 1 indicated disagreement or absence, 2 indicated limited application or neutrality, and 3 indicated adequate application. This framework was included because ecotourism should not merely expose passengers to wildlife. It should also promote reflective understanding, ethical awareness, and conservation-oriented thinking.

### 2.4. Data Treatment

Results are presented descriptively as criterion-level scores and category averages. Given the small number of observed trips and the practical nature of the scoring system, the study is best interpreted as a baseline comparative assessment rather than a basis for strong inferential claims. As such, a descriptive assessment was used, and no inferential testing was conducted. Nonetheless, these descriptive patterns are informative because they reveal where current practice aligns with, or falls short of, standards directly relevant to cetacean welfare and ecotourism quality.

## 3. Results

Across frameworks, certified operators generally scored higher than uncertified operators, but the differences were not large enough to support the view that certification alone reliably protected cetacean welfare or ensured meaningful ecotourism. In both the educational and vessel-behavior components, there was overlap between certified and uncertified operators, as well as notable variability within the certified group itself.

### 3.1. Educational Quality and the Ecotourism Attributes of Whale Watching

Under Whale SENSE educational criteria (Table 1), certified operators (U1-U4) averaged 2.00/3, whereas uncertified operators (U5-U8) averaged 1.69/3. This pattern suggests that certified operators generally provided somewhat better interpretive content, but the overall level remained modest. Even among certified operators, scores indicated that laws, threats, conservation measures, and whale-watching guidelines were often mentioned only briefly rather than explained in a way likely to build strong conservation understanding.

The WCA educational criteria (Table 2) reinforced this conclusion and highlighted additional weaknesses. Certified operators averaged 2.21/3 compared with 1.38/3 for uncertified operators. However, all operators scored poorly on multilingual accessibility, indicating that the interpretation was not being designed as an intentional conservation intervention, but rather as a secondary service layered onto wildlife viewing.

The HOTS assessment (Table 3) showed the largest discrepancy. All commercial operators scored at the minimum level for higher-order-thinking opportunities, indicating that narration rarely moved beyond one-way information delivery. A small subset of certified operators provided modest interactivity, but structured prompts for tourists encouraging reflection, evaluation, or moral reasoning were largely absent.

### 3.2. Vessel Behavior and Welfare-Relevant Disturbance Risk

All observed trips included at least one whale encounter. Under the Whale SENSE vessel-behavior criteria (Table 4), certified operators averaged 4.00/5, while uncertified operators averaged 2.89/5. On its face, this suggests that certification was associated with better vessel handling. However, the pattern was uneven. Two certified operators scored below the level that would indicate consistently strong compliance, and some of the weakest behaviors involved issues with direct welfare relevance, including close approaches, path restriction, and departure before animals were fully clear of the vessel.

The WCA vessel-behavior framework revealed a sharper compliance gap (Table 5). Certified operators averaged 2.50/3 on best-practice items, compared with 1.53/3 for uncertified operators, but several problems remained common across the sector. Most notably, all operators failed the WCA criterion requiring that vessels not approach whales within 100 m (or dolphins and porpoises within 50 m), and most operators also failed to consistently reduce to no-wake speed within 300 m. These results identify approach distance and speed reduction as the most consistent areas of non-compliance under the WCA framework.

Practices recorded under the WCA framework are summarized in Table 6. Certified operators averaged 0.50 practices, whereas uncertified operators averaged 2.00. The most severe infractions were concentrated in uncertified operators. Thus, although certification appeared beneficial on average, isolated unacceptable practices also occurred among certified operators.

## 4. Discussion

One of the central implications of this study is not that every observed encounter caused measurable harm, but that inconsistent adherence to disturbance-minimizing practices can increase disturbance risk and narrow the precautionary buffer that responsible whale-watching guidelines are designed to maintain. Several common vessel behaviors eroded the protective buffer intended by responsible whale-watching guidelines [7,8,9,10,11,12,13,14,15,16,17,18,21,33,35]. Across the whale-watching literature, disturbance is increasingly understood as a welfare issue because vessel proximity, speed, angle of approach, repeated passes, and acoustic exposure can alter how cetaceans move, communicate, rest, and allocate attention, even when obvious injury is absent [7,8,9,10,11,12,13,14,15,16,17,18,19,20,35]. In that sense, poor vessel conduct is not merely a technical failure to follow rules; instead, it shifts the burden of coping with the interaction onto the whale, which must alter its behavior or attention in response to the disturbance.

This distinction is especially important in free-ranging systems, where impacts are often cumulative, context-dependent, and behaviorally mediated rather than immediately visible [7,8]. Vulnerability is shaped not only by species identity but also by life-history stage, local traffic density, social context, and the predictability of repeated encounters [16,17,18]. In the New York Bight, this concern is amplified by an already complex acoustic and vessel environment [11,19,30]. Additional close approaches or erratic maneuvers by tourism vessels therefore occur against a background of existing anthropogenic pressure rather than in an otherwise quiet seascape. Even when strikes do not occur, reducing clearance distance, delaying neutral gear, or cutting across travel paths can increase welfare-relevant disturbance risk and narrow the safety margin intended by precautionary guidance [7,8,9,10,11,12,13,14,15,20,33,35].

The educational findings in this study are equally important because whale watching is frequently defended as more than leisure. It is often presented as ecotourism on the grounds that it can cultivate knowledge, empathy, pro-conservation intentions, and public support for marine protection [4,5,6,22,23,24,25,26,27,28,29,36,37]. However, ecotourism should not be treated as inherently conservation-positive simply because it occurs in nature or involves charismatic wildlife. Rather, its conservation value depends on whether tourism creates credible pathways from wildlife encounter to learning, concern, responsible behavior, and support for protection [35,36]. The literature also shows that these outcomes do not arise automatically from seeing whales alone. Tourists are more likely to leave with stronger conservation intentions when tours provide structured, emotionally engaging, and behaviorally meaningful guidance rather than fragmented facts or generic enthusiasm [5,6,23,25,27,28,29]. In the present study, narration was often shallow and minimally interactive on precisely those topics that most justify ecotourism claims, including legal protections, anthropogenic threats, conservation measures, and the responsibilities of both operators and passengers. This was reflected in the minimum higher-order-thinking scores assigned to all commercial operators, indicating limited opportunities for passengers to apply, analyze, evaluate, or interact meaningfully with the conservation content.

This matters pedagogically. If animals are exposed to tourism-related disturbance, the educational return should be substantial rather than symbolic. Prior work has argued that whale watching becomes more defensible when interpretation is linked to clear conservation objectives and when visitors are helped to connect the encounter with responsible future behavior [3,4,5,6,22,25,26,27,28,29]. Conversely, weak interpretation risks turning wildlife encounters into a spectacle accompanied by light commentary. The consistently low HOTS scores indicate more than a missed teaching opportunity: they suggest that educational programming across the sector may be falling short of ecotourism’s goal of providing meaningful educational experiences, and that the conservation value often invoked to justify cetacean tourism may be thinner than operators and consumers assume [22]. The poor multilingual performance deepens this problem in a major international tourism region by restricting who can fully access the educational component. In an international urban tourism setting, limited multilingual interpretation may constrain equitable access to conservation education and reduce the broader conservation reach of whale-watching ecotourism, particularly when international visitors are unable to fully engage with welfare guidelines, threat narratives, and responsible-viewing messages [38].

Certified operators generally outperformed uncertified operators, and this should not be dismissed. The results suggest that certification can improve average practice and may serve as an important management tool [17,21,24]. However, the broader whale-watching literature has shown that the existence of standards does not guarantee consistent field performance, especially when monitoring is intermittent and enforcement weak [17,18,26,32]. The present findings fit that pattern: certification appears to help, but it did not eliminate welfare-relevant shortcomings, nor did it ensure educational depth consistent with strong ecotourism standards [35,36,37]. For that reason, certification should be understood as a partial safeguard rather than a definitive guarantee of responsible whale watching.

The contrast between Whale SENSE and WCA scores is particularly instructive. Some operators appeared broadly acceptable under the local framework but less so under the more precautionary international standard [21,33]. That pattern suggests that the apparent adequacy of a whale-watching operation depends strongly on which threshold of responsibility is applied. From an animal-welfare perspective, this matters because conservative thresholds preserve cetacean control over the interaction and reduce the chance that repeated tourism encounters become cumulative stressors [16,17,18,26]. From an ecotourism perspective, it matters because the credibility of a ‘responsible’ brand depends not merely on meeting a manageable local threshold, but on demonstrating genuinely protective practice.

This study offers several practical recommendations for responsible whale watching. First, vessel standards should be treated as welfare measures and enforced accordingly. Post-certification monitoring, refresher training, and clearer behavioral thresholds for approach speed, positioning, and departure would help reduce the gap between formal certification and observed practice [17,21,24,26,33]. Second, interpretation should be judged by educational quality rather than topic presence alone. Operators should explain why distance, speed reduction, and passenger conduct matter for cetacean welfare, not merely mention rules as procedural requirements. This is consistent with prior studies showing that the conservation value of wildlife tourism improves when interpretation is intentional, structured, and connected to visitor responsibility [5,6,22,25,27,28,29]. Third, whale-watching operators could strengthen ecotourism value without radically redesigning tours. Pre-departure briefings, multilingual digital materials, concise explanations of local conservation issues, and guided prompts that encourage visitors to reflect on respectful wildlife viewing are all realistic interventions. Importantly, the literature does not suggest that stronger welfare protections inevitably reduce visitor satisfaction; rather, tourists often respond positively when tours are perceived as informative, well-managed, and ethically grounded [23,25,28,29]. In other words, welfare-oriented practice and tourist-centered tourism can be mutually reinforcing. A more coherent educational strategy may help operators protect cetaceans while also strengthening the legitimacy and long-term resilience of the industry [3,16,26].

This study should be interpreted as a baseline descriptive assessment. The sample was limited to one metropolitan region and a single field season, and the number of observed trips was necessarily modest. The study also did not measure direct behavioral or physiological outcomes in cetaceans, so it cannot quantify the actual magnitude of welfare impact arising from specific vessel encounters. Instead, it identifies practices that increase or decrease welfare-relevant disturbance risk based on recognized best-practice frameworks. Even so, baseline studies remain valuable in understudied urban marine systems, particularly where whale occurrence is increasing, and animals are already embedded within complex vessel and soundscapes [19,30]. In addition, criterion-based observational scoring necessarily involves some judgment even when standardized rubrics are used. The use of paired observers reduced this limitation, but it does not remove it entirely. Finally, this study evaluated educational quality through delivered content and structure, not through passenger learning outcomes. Future research would be strengthened by integrating tourist surveys, repeated monitoring across multiple seasons, and direct behavioral or acoustic measures that connect vessel practice and interpretive style more explicitly to cetacean welfare and conservation-related human outcomes [17,19,20,26,27,28,29].

## 5. Conclusions

This study provides a welfare-risk assessment of whale watching in the New York City Metropolitan Area by evaluating both how operators behaved around cetaceans and what they taught passengers about them. The two findings are closely linked. Vessel behavior showed that certification was associated with better average performance, but not with consistently precautionary practice. Educational programming showed that conservation interpretation was often too shallow to fully support the ecotourism claims made on behalf of whale watching. Together, these results suggest that responsible whale watching should be defined by more than market success or certification status alone.

To justify its claim as ethical ecotourism, whale watching must both limit unnecessary disturbance to free-ranging cetaceans and offer substantive educational experiences that deepen public understanding of conservation. In the present study, both obligations were met only partially. Accordingly, future industry improvement should focus not only on stricter vessel compliance, but also on richer, more accessible, and more reflective interpretation. Protecting cetacean welfare and preserving the integrity of ecotourism are not separate goals; in whale watching, they are part of the same standard of responsibility.

## Figures and Tables

**Table 1:** Whale SENSE educational evaluation scores.

Criterion	U1	U2	U3	U4	U5	U6	U7	U8
Whale protection laws	2	2	2	2	2	2	1	1
Whale threats	2	3	3	1	2	2	1	1
Whale conservation measures	1	2	2	1	2	3	1	1
Whale-watch guidelines	2	3	3	1	2	2	2	2
Average	1.75	2.5	2.5	1.25	2.0	2.25	1.25	1.25

Scoring: 1 = not mentioned/disagree; 2 = mentioned briefly/neutral; 3 = discussed in detail/agree. Certified Operators = U1–U4; Uncertified Operators = U5–U8.

**Table 2:** World Cetacean Alliance educational evaluation scores.

Criterion	U1	U2	U3	U4	U5	U6	U7	U8
Advance materials accessible to multiple language users	1	1	1	1	1	1	1	1
Explains potential impacts on cetaceans and the marine environment	3	3	2	3	1	1	1	1
Explains why guidelines matter and how they are followed	1	3	3	1	2	1	1	1
Provides a health and safety briefing	3	3	2	1	3	1	1	1
Manages expectations about the activity	3	3	3	3	3	3	3	1
Provides education on threats to animals and the environment	2	2	2	3	1	1	1	1
Average	2.17	2.5	2.17	2.0	1.83	1.33	1.33	1.0

Scoring: 1 = disagree/not addressed; 2 = neutral/limited treatment; 3 = agree/addressed in adequate detail. Certified Operators = U1–U4; Uncertified Operators = U5–U8.

**Table 3:** HOTS evaluation scores.

Criterion	U1	U2	U3	U4	U5	U6	U7	U8
Higher-order thinking opportunities	1	1	1	1	1	1	1	1
Interactivity	2	2	1	2	1	1	1	1
Average	1.5	1.5	1.0	1.5	1.0	1.0	1.0	1.0

Higher scores indicate stronger evidence of interactive, reflective, or cognitively demanding interpretation. Certified Operators = U1–U4; Uncertified Operators = U5–U8.

**Table 4:** Whale SENSE vessel-behavior scores.

Criterion	U1	U2	U3	U4	U5	U6	U7	U8
Coordinates viewing time with other vessels	3	3	5	3	3	3	1	3
Approaches from behind when 600 ft or closer	5	5	5	3	2	2	1	2
Does not attempt a head-on approach within 600 ft	5	5	5	3	4	4	4	4
Does not intentionally approach within 100 ft	2	4	4	3	2	2	1	5
If approached within 100 ft, engines in neutral	4	5	5	3	4	4	1	5
Does not cut off a traveling animal	5	5	5	2	3	4	1	2
Departs at a slow, safe speed	4	4	5	4	4	4	4	5
Departs only after the animal is clear of the vessel	2	5	5	2	4	4	2	4
Average	3.75	4.5	4.88	2.88	3.25	3.38	1.88	3.75

Scoring: 1 = strongly disagree; 2 = disagree; 3 = neutral; 4 = agree; 5 = strongly agree. Certified Operators = U1–U4; Uncertified Operators = U5–U8.

**Table 5:** World Cetacean Alliance best-practice vessel-behavior scores.

Criterion	U1	U2	U3	U4	U5	U6	U7	U8
Knowledgeable guide on board	3	3	3	3	3	3	3	1
Cetaceans have the right of way; the vessel does not restrict movement	3	3	3	3	3	1	1	1
Approaches from the side and slightly behind	3	3	3	3	2	1	1	1
No-wake speed within 300 m	1	1	1	3	3	1	1	1
Does not approach whales within 100 m/dolphins within 50 m	1	1	1	1	1	1	1	1
If a cetacean approaches, engines placed in neutral	3	3	3	3	1	1	1	3
Does not return to the same cetaceans during the trip	3	3	3	3	1	1	1	1
Attempts to visit different cetaceans on each daily trip	3	2	2	3	2	3	1	2
Average	2.5	2.38	2.38	2.75	2.0	1.5	1.25	1.38

Scoring: 1 = disagree/not met; 2 = neutral/partially met; 3 = agree/met. Certified Operators = U1–U4; Uncertified Operators = U5–U8.

**Table 6:** World Cetacean Alliance unacceptable-practice scores.

Criterion	U1	U2	U3	U4	U5	U6	U7	U8
Deliberate chasing absent	0	0	0	0	1	0	1	0
No rubbish or food is thrown overboard	1	0	0	0	0	1	1	0
No excessive loud or disturbing noise	0	0	0	1	0	0	0	0
No leap-frogging	0	0	0	0	0	0	1	0
No scattering or separation of the cetacean group	0	0	0	0	0	1	1	0
No food provisioning	0	0	0	0	0	0	0	0
No corralling	0	0	0	0	0	1	0	0
Summed score	1	0	0	1	1	3	4	0

Scoring: 0 = absent; 1 = present. The final row is the summed count of unacceptable practices recorded for each operator, where a higher score indicates lower compliance. Certified Operators = U1–U4; Uncertified Operators = U5–U8.

## Data Availability

Data is contained within the article.

## Supplementary Materials

The following supporting information can be downloaded at https://www.mdpi.com/article/10.3390/ani16131955/s1. File S1. Supplementary Documents Package.
