# Supplementary material for: Cetacean Welfare Risk and the Educational Integrity of Ecotourism: A Multi-Framework Assessment of Whale-Watching Practices in the New York Metropolitan Area
Source: Animals (Basel). 2026 Jun 24;16(13):1955. doi: 10.3390/ani16131955 (PMC13360588; doi:10.3390/ani16131955)
Supplement: Supplementary file 1 [file animals-16-01955-s001.zip › animals-4300959-supplementary.pdf]

Supplementary Documents Package

Whale SENSE "On the Water" evaluation:

Education
The main categories scored for education were as followed:

Whale protection laws
Whale threats
Whale conservation measures
Whale-watch guidelines

Scored as:

Agree/Addressed in detail (Score of 3)
Mentioned briefly/neutral (Score of 2)
Disagree/ not addressed (Score of 1)

Whale protection laws were scored at a 3 when the operator discussed, in detail, both the Marine Mammal Protection Act of 1972 (MMPA), and the Endangered Species Act of 1973 (ESA). "In detail" signified that the operator discussed the content of both pieces of legislation with some specific details, such as the 'harassment' clause of the MMPA and its application. Mentioned with no detail scored a 2 and demonstrated that the operator mentioned one or both of these pieces of legislation, but only shallowly. Most operators fell into this category; most operators mentioned the existence of the MMPA but never mentioned the contents of the act beyond a simple 'it protects whales and dolphins.' No operators mentioned the ESA. Two operators did not mention the MMPA.

Whale threats was scored at a 3 when the operator discussed, in detail, real local threats to whales such as noise pollution, vessel strikes, or water pollution (list not exhaustive) rather than hypothetical or non-local threats, such as discussing threats to non-local species in lieu of real local threats. Climate change was included when operators made a clear connection to local populations of whales, such as increasing water temperatures in the southern range of Humpback whales affecting migration patterns of this species along its route through the NY/NJ Bight. Mentioned with no detail scored a 2, demonstrating a shallow mention of a single threat or a non-local (relevant) threat such as "whales continue to be hunted in some parts of the world." Three operators did not mention threats to whales at all during the narration.

Whale conservation measures was scored at a 3 when the operator discussed, in detail, actionable items tourists could take in their daily lives to improve the welfare of whales. This included items such as join a stranding network and learn about the process for rescuing stranded cetaceans, pulling plastics out of the water or joining a beach clean up, and writing to local representatives. Operators received a score of two when the description was shallow; all three operators to receive a two simply said, "Recycle," with no context and no direction. Half made no mention of conservation measures at all.

Whale-watch guidelines was scored at a 3 when the operator discussed best practices for tourist and vessel behavior including both the item and its purpose such as keeping noise to a minimum during viewing to avoid impacting cetacean behavior and not throwing trash into the water to prevent harm to wildlife through consumption for tourists and reminding tourists that vessels must maintain a safe distance from cetaceans to prevent behavioral disturbance. A score of 2 was given when there was only discussion of tourist behavior or no discussion as to why the item was important or the item had no immediate bearing on cetaceans such as "No running on deck." Operators who received a 2 did not mention the importance of vessel adherence to guidelines. A score of 1 signified no mention of guidelines at all for tourists or the vessel.

Vessel Behavior
The main categories scored for vessel behavior were as followed:

Coordinates viewing time with other vessels
Approaches from behind when 600 ft or closer
Does not attempt a head-on approach within 600 ft
Does not intentionally approach within 100 ft
If approached within 100 ft, engines in neutral
Does not cut off a traveling animal
Departs at slow, safe speed
Departs only after animal is clear of vessel

With a pre-determined selection of:

Strongly Agree (Score of 5)
Agree (Score of 4)
Not Applicable (Score of 3)
Disagree (Score of 2)
Strongly Disagree (Score of 1)

Note:

Coordinates viewing time with other vessels
Operators received a 5 when the captain actively communicated with other vessels about the length of viewing time each time the vessel viewed cetaceans (whales and dolphins). A score of 4 was granted when the captain regularly reported their location and coordinated with other vessels. A score of 3, not applicable, was given when the vessel reported that they had not viewed cetaceans in the presence of other vessels. A score of 2, disagree, applied to vessels that did not regularly communicate with other vessels while a score of 1, strongly disagree was reserved for vessels that did not communicate at all. A secondary operator confirmed the score of 1 for U7, reporting that U7 did not communicate with them despite regularly being at the same viewing locations.

Approaches from behind when 600 ft or closer
Distances were assessed based on the vessel length; most vessels were between 95ft and 120ft, with the largest vessel at 157ft. A score of 5, or strongly agree, indicated that the vessel was in compliance during every interaction with whales. A score of 4, or agree, indicated that the vessel was in compliance during the majority of the encounters with whales (example: 2 of 3 encounters). A score of 3, or not applicable, indicated that the observers were unable to determine with certainty whether the distance was maintained during the single whale sighting. A score of 2, or disagree, indicated that the majority of interactions with whales failed to meet the 600ft cut off, while a score of 1 indicated all interactions with whales failed to meet the 600ft guideline. U4, which received a 3, was an outlier for vessel behavior; while most vessels were either conservative with their distance allowances or were in clear violation, U4 approached from behind but the distance was borderline.

Does not attempt a head-on approach within 600 ft
A score of 5, or strongly agree, indicated that the vessel was in compliance during every interaction with whales. A score of 4, or agree, indicated that the vessel was in compliance during the majority of the encounters with whales. A score of 3, or not applicable, indicated that the observers were unable to determine with certainty whether the distance was maintained during the single whale sighting. U4 did not attempt a head-on approach but the distance was borderline. A score of 2, or disagree, indicated that the majority of interactions with whales failed to meet the 600ft cut off, while a score of 1 indicated all interactions with whales failed to meet the 600ft guideline.

Does not intentionally approach within 100 ft
A score of 5, or strongly agree, indicated that the vessel was in compliance during every interaction with whales. A score of 4, or agree, indicated that the vessel was in compliance during the majority of the encounters with whales. A score of 3, or not applicable, indicated that the operator did not do enough to meet the ‘agree’ category, but also did not wholly violate the guideline. U4 cut engines around 100ft but continued to drift closer to the whale. U4 did not speed up significantly before cutting engines. A score of 2, or disagree, indicated that the majority of interactions with whales failed to meet the 100ft cut off or that the operator engaged in questionable behavior such as increasing speed before placing engines in neutral. A score of 1 indicated all interactions with whales failed to meet the 100ft guideline.

If approached within 100 ft, engines in neutral

A score of 5, or strongly agree, indicated that the vessel was in compliance during every interaction with whales. A score of 4, or agree, indicated that the vessel was in compliance during the majority of the encounters with whales. A score of 3, or not applicable, indicated that the operator did not do enough to meet the 'agree' category, but also did not wholly violate the guideline, such as placing engines in neutral but maneuvering the vessel closer to the whales. A score of 2, or disagree, indicated that the majority of interactions with whales failed to meet the 100ft cut off or that the operator engaged in questionable behavior such as increasing speed before placing engines in neutral. A score of 1 indicated all interactions with whales failed to meet the 100ft guideline.

Does not cut off a traveling animal

A score of 5, or strongly agree, indicated that the vessel was in compliance during every interaction with whales or dolphins. A score of 4, or agree, indicated that the vessel was in compliance during the majority of the encounters with whales or dolphins. A score of 3, or not applicable, indicated a cetacean had submerged within 100ft of the vessel and was not visible at the time the vessel began moving. A score of 2, or disagree, indicated that the majority of interactions with whales or dolphins failed to meet the guideline; most often vessels were more cautious of whales than dolphins. A score of 1 indicated all interactions with whales and dolphins failed to meet the guidline.

Departs at slow, safe speed

A score of 5, or strongly agree, indicated that the vessel was in compliance during every interaction with whales or dolphins. A score of 4, or agree, indicated that the vessel was in compliance during the majority of the encounters with whales or dolphins. A score of 3, or not applicable, indicated that cetaceans were submerged for the majority of interactions and a determination was not able to be made. A score of 2, or disagree, indicated that the majority of interactions with whales or dolphins failed to meet the guideline with dangerous speeds based on proximity to the cetaceans being viewed. A score of 1 indicated all interactions with whales and dolphins failed to meet the guidline.

Departs only after animal is clear of vessel

A score of 5, or strongly agree, indicated that the vessel was more than 50ft away from cetaceans at the time of departure. A score of 4, or agree, indicated that the vessel was in compliance during the majority of the encounters with whales or dolphins. A score of 3, or not applicable, indicated that cetacean was submerged for the majority of interactions and a determination was unable to be made. A score of 2, or disagree, indicated that the majority of interactions with whales or dolphins failed to meet the guideline with cetaceans within 50ft of the vessel. A score of 1 indicated all interactions with whales and dolphins failed to meet the guidline.

WCA Global Best Practice Guidance for Responsible Whale and Dolphin Watching:

Best Practice: Education
The main categories scored for education were as followed:

Advance materials accessible to multiple language users
Explains potential impacts on cetaceans and marine environment
Explains why guidelines matter and how they are followed
Provides health and safety briefing
Manages expectations about the activity
Provides education on threats to animals and environment

Scored as:

Agree/Addressed in adequate detail (Score of 3)
Not mentioned/ Neutral (Score of 2)
Disagree/ not addressed (Score of 1)

Advance materials accessible to multiple language users
A score of 3, or agree, indicated that the vessel had multilingual information. A score of 2, or neutral, indicated there was some incomplete information in other languages than English. A score of 1 indicated English only.

Explains potential impacts on cetaceans and marine environment
A score of 3, or agree, indicated that the narration on board included detailed information such as discussion of strikes as a result of increased shipping volume in the NY/NJ Bight. A score of 2 indicated there was information briefly mentioned but without meaningful detail or discussion such as stating more simply, "strikes are problematic." A score of 1 indicated no mention.

Explains why guidelines matter and how they are followed
A score of 3, or agree, indicated that the narration on board included detailed information such as discussion of distance requirements and how cetaceans may be impacted by close encounters. A score of 2 indicated there was information briefly mentioned but without meaningful detail or discussion such as stating more simply, "we must maintain a safe distance." A score of 1 indicated no mention.

Provides health and safety briefing
A score of 3, or agree, indicated that the narration on board included detailed information such as prohibiting small children from leaning over railings on deck. A score of 2 indicated there were a few safety issues briefly mentioned but without detail, such as no running, no climbing, etc. A score of 1 indicated no mention.

Manages expectations about the activity
A score of 3, or agree, indicated that the narration on board included detailed information about the process of going out to the viewing sites and returning, probability of seeing cetaceans and why, an account of what to expect once cetaceans are located, etc. A score of 2 indicated there was information briefly mentioned but without meaningful detail. A score of 1 indicated no mention.

Provides education on threats to animals and environment
A score of 3, or agree, indicated that the narration on board included detailed information regarding threats to cetaceans and the environment, such as noise pollution or vessel strikes from competition with shipping lanes. A score of 2 indicated there was information briefly mentioned but without meaningful detail or discussion such as stating more simply, "strikes sometimes happen." A score of 1 indicated no mention.

WCA Best Practice: Vessel Behavior
The main categories scored for education were as followed:

Knowledgeable guide on board
Cetaceans have right of way; vessel does not restrict movement
Approaches from the side and slightly behind
No-wake speed within 300 m
Does not approach whales within 100 m / dolphins within 50 m
If cetacean approaches, engines placed in neutral
Does not return to the same cetaceans during trip
Attempts to visit different cetaceans on each daily trip

Scored as:

Agree/Addressed in adequate detail (Score of 3)
Not mentioned/ Neutral (Score of 2)
Disagree/ not addressed (Score of 1)

Knowledgeable guide on board
A score of 3, or agree, indicated that there was a naturalist on board with specialist training in marine biology or marine ecology. A score of 2, or neutral, indicated there was a naturalist on board with some training, such as a current student in a related field. A score of 1 indicated no relevant education or training.

Cetaceans have right of way; vessel does not restrict movement
A score of 3, or agree, indicated that vessel was consistently compliant. A score of 2, or neutral, indicated the vessel was occasionally noncompliant and/or cetaceans were submerged for some portion of the interaction. A score of 1 indicated the vessel was consistently noncompliant.

Approaches from the side and slightly behind
A score of 3, or agree, indicated that vessel was consistently compliant. A score of 2, or neutral, indicated the vessel was occasionally noncompliant. A score of 1 indicated the vessel was consistently noncompliant.

No-wake speed within 300 m
A score of 3, or agree, indicated that vessel was consistently compliant. A score of 2, or neutral, indicated the vessel was not consistently compliant. A score of 1 indicated the vessel was consistently noncompliant.

Does not approach whales within 100 m / dolphins within 50 m
A score of 3, or agree, indicated that vessel was consistently compliant. A score of 2, or neutral, indicated the vessel was not consistently compliant. A score of 1 indicated the vessel was consistently noncompliant.

If cetacean approaches, engines placed in neutral
A score of 3, or agree, indicated that vessel was consistently compliant. A score of 2, or neutral, indicated the vessel was not consistently compliant or cetacean was submerged during approach. A score of 1 indicated the vessel was consistently noncompliant.

Does not return to the same cetaceans during trip
A score of 3, or agree, indicated that vessel was consistently compliant. A score of 2, or neutral, indicated the vessel was not consistently compliant. A score of 1 indicated the vessel was consistently noncompliant.

Attempts to visit different cetaceans on each daily trip

A score of 3, or agree, indicated that the vessel reported in the affirmative. A score of 2, or neutral, indicated the vessel reported that they occasionally complied. A score of 1 indicated the vessel reported in the negative.

Unacceptable practices
Deliberate chasing absent
No rubbish or food thrown overboard
No excessive loud or disturbing noise
No leap-frogging
No scattering or separation of cetacean group
No food provisioning
No corralling
Higher score indicates lower compliance

Scored as:

Absent (Score of 0)
Present (Score of 1)

If the behavior was observed, it was scored a 1.

HOTS Assessment
Higher Order Thinking Opportunities
Interactivity

Scored as:

Agree (Score of 3)
Present but shallow/irrequglar (Score of 2)
Disagree/none (Score of 1)

Higher Order Thinking Opportunities
Analyzing:
Ex. Identify a problem and a solution in a story told about cetaceans.
Evaluating:
Ex. Assess the benefits of baleen vs teeth in cetaceans.
Synthesis:
Ex. Consider writing a letter to your representative advocating for cetaceans.
Interactivity
Naturalist on board prompts between:<br>1) visitors and staff<br>2) visitors amongst themselves

Higher Order Thinking Opportunities
Originally this assessment included a checklist for question types based on Bloom's taxonomy in categories for analyzing, evaluating, and synthesis. However, it became clear that none of the operators were asking questions that fall into these three categories. For this reason, the original assessment was condensed to the current form with a broad category for HOTS Opportunities.

Interactivity
A score of 3, or agree, indicated that the naturalist on board actively encouraged interaction with visitors through asking probing or bridging questions, encouraging guests to visit with staff on board and ask their own questions, and discussion prompts that encouraged visitors to discuss amongst themselves. A score of 2, or neutral, indicated there was some attempt made to engage, such as tacking on an, "Ask a staff member if you have any questions" at the end of the health and safety briefing. A score of 1 indicated there was no attempt to directly engage visitors.

Whale SENSE 2022 On-The-Water Evaluation

Reminder: Collect videos and photos at minimum to send in with this form

Date

Time

Evaluator Name

Captain Name

Naturalist Name

Whale Watch Company

▶▶▶

Did you see the Whale SENSE placard posted on the vessel and/or in the ticket booth?

Yes Where?

No

Did you see whale watch guidelines posted on the vessel and/or in ticket booth?

Yes Where?

No

Operational Compliance with Whale Watch Guidelines

Time of First Whale Sighting

Species Sighted

# of Animals in Group

Time Spent on Animal/Group

Other vessels present (select all that apply)

- Commercial whale watch
- Recreational vessel
- Commercial fishing vessel
- Recreational fishing vessel
- Other

Animal behavior on vessel approach (select all that apply)

- Feeding
- Logging
- Traveling
- Breaching
- Other active behaviors

Were whale calves present?

Yes No

Disclaimer: Form criteria is limited to criteria that are usually easily observable, but we are aware that evaluators are not guaranteed to accurately capture all criteria despite their best efforts.

Please indicate how much you agree or disagree with the following statements as they relate to the vessel operation around the first sighted whale or dolphin.

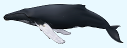 *Applies to all large whale viewing except right whales*

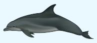 *Applies to dolphin viewing*

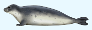 *Applies to seal viewing*

	Strongly Agree	Agree	Unknown/ Not Applicable	Disagree	Strongly Disagree
Lookout is posted when animal is sighted 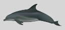 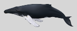					
Vessel coordinates viewing time with other vessels 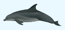 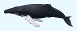					
When 600 feet or closer to whale, vessel approaches from behind 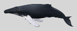					
When 600 feet or closer to whale, vessel does **NOT** attempt a head-on approach. 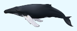					
Vessel does **NOT** intentionally approach whale within 100 feet. 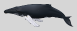					
If approached by animal within 100 feet, vessel places engines in neutral 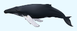					
Vessel does **NOT** cut off traveling animal's path. 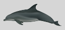 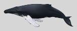					
Vessel departs animal at slow safe speed 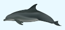 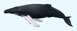					
Vessel departs area **ONLY** after animal is known to be clear of the vessel. 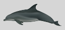 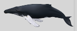					
Animal does **NOT** abruptly change directions or show signs of potential disturbance at the approach of the vessel. 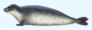 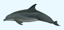 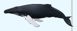					

**Marine mammal illustrations courtesy of Uko Gorter*

Disclaimer: Form criteria is limited to criteria that are usually easily observable, but we are aware that evaluators are not guaranteed to accurately capture all criteria despite their best efforts.

Marine Mammal Viewing Guidelines (Continued)

	Strongly Agree	Agree	Not Applicable	Disagree	Strongly Disagree
Vessel does **NOT** intentionally approach dolphins or seals within 50 yards (150 feet) 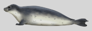 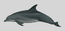					
If approached within 150 feet by dolphins, vessel places engines in neutral. 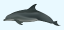					
Vessel does **NOT** intentionally cause dolphin to bow ride or wake surf 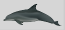					
Vessel does **NOT** flush seals from haul out site. 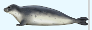					

Additional comments on vessel behavior (concerns and compliments). Please be specific. Provide photos if necessary.

**Marine mammal illustrations couresy of Uko Gorter*

Disclaimer: Form criteria is limited to criteria that are usually easily observable, but we are aware that evaluators are not guaranteed to accurately capture all criteria despite their best efforts.

Naturalist Narration

Please check the topics that were discussed by the naturalist during the whale watch.

	Discussed in detail	Mentioned but no detail	Not mentioned	Not sure
Whale protection laws				
Whale threats				
Whale conservation measures				
Whale watch guidelines				
Whale SENSE				
Stellwagen Bank National Marine Sanctuary*				

*Naturalist mention of Stellwagen Bank National Marine Sanctuary is only required of Massachusetts based companies

Any additional comments (concerns and compliments) on whale watch narration:

Any additional information that may be helpful to know? i.e. sea/weather conditions, # of passengers vs vessel capacity, audio quality?

Disclaimer: Form criteria is limited to criteria that are usually easily observable, but we are aware that evaluators are not guaranteed to accurately capture all criteria despite their best efforts.

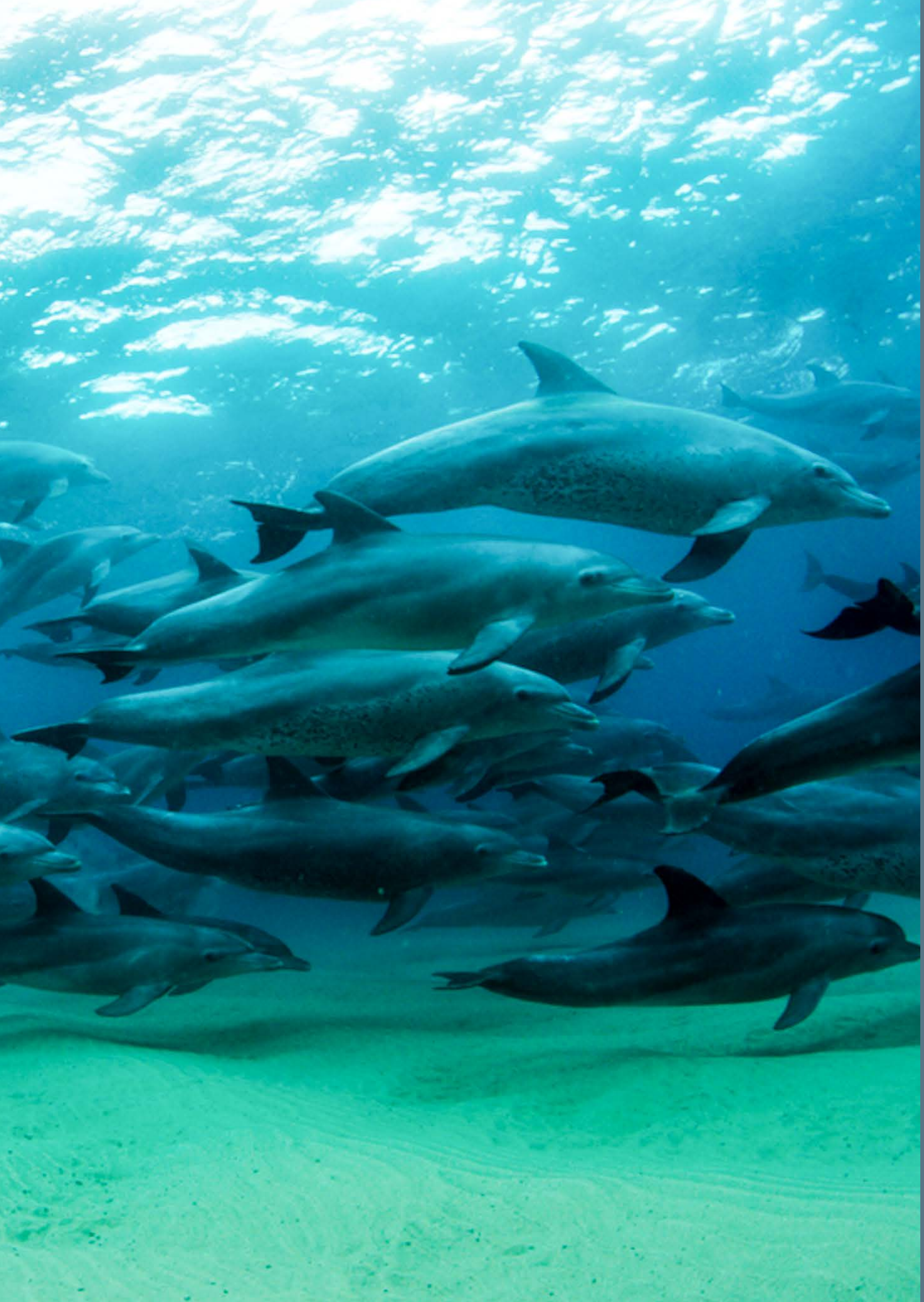

GLOBAL BEST PRACTICE GUIDANCE FOR RESPONSIBLE WHALE AND DOLPHIN WATCHING

TOURISM ACTIVITIES INVOLVING WILD CETACEANS

SOPHIE LEWIS & DYLAN WALKER

GLOBAL BEST PRACTICE GUIDANCE FOR RESPONSIBLE WHALE AND DOLPHIN WATCHING:
Tourism activities involving wild cetaceans. A guide by the World Cetacean Alliance with support from ClubMed.

Requests and enquiries should be addressed to:

Responsible Cetacean Tourism Manager
World Cetacean Alliance (WCA) Headquarters
Studio 3, Lower Promenade,
Madeira Drive,
Brighton,
BN2 1ET, UK

Club Med Contact details:

Sustainability Manager
Sustainability Direction –
Club Med,
11, rue de Cambrai,,
75957, Paris, Cedex 19,
France

Lead author: Sophie Lewis
Author and editor: Dylan Walker
Design: Ailsa Marrs
Infographics: Giulia De Amicis / behance

© 2018 WCA / ClubMed. No part of this document may be reproduced by any process without prior written permission from the authors.

Citation: Lewis, S. & Walker, D. (2018). Global Best Practice Guidance for Responsible Whale and Dolphin Watching: Tourism activities involving wild cetaceans. A guide by the World Cetacean Alliance with support from ClubMed. Brighton, UK.

Disclaimer: This guide is based on information gathered and analysed by WCA through interviews with tour operators, government, non-government, academic, and other stakeholders as well as a review of published research and existing guidelines and regulation. The findings of this guide are dependent on that information which was available at the time of the research, and reflects best practice as defined by industry experts and published scientific research; interpreted and collated by the authors.

The views and opinions expressed in this report are those of the World Cetacean Alliance and do not necessarily reflect the views or positions of contributors.

Cover image © Angie Gullan/ Dolphin Encountours

FOREWORD

Tourism constitutes 10% of the global economy with significant impacts on society and the environment. It has the potential, financial support, and increasing will, to invest in sustainability, local community, and environmental enhancement programmes with a clear benefit for ocean conservation. The way that people connect with whales and dolphins (cetaceans) across the world is strongly associated with tourism. Wild whale and dolphin watching is a US$2.1 billion a year industry which continues to show strong growth in many regions.

When poorly managed, whale and dolphin watching tourism in all its forms has been shown to negatively impact cetaceans, compromising welfare, and potentially even causing declines in populations, with likely implications for the health of associated marine ecosystems. By contrast, well managed whale and dolphin watching tours put the welfare and conservation of the species they wish to encounter first.

At Club Med, we strive for sustainable and responsible tourism practices across our network of resorts and visitor experiences. Seeking improvements in the care of animals experienced through tourism has become a larger part of our work in recent years, and whilst we feel we still have work to do, we are confident to be moving in the right direction.

As part of our efforts to progressively ensure that any wild whale and dolphin watching tours taken by Club Med holidaymakers meet the highest standards of customer care, animal welfare, and education, we commissioned the World Cetacean Alliance (WCA) to write this report to assist both Club Med and other tour operators when auditing for best practice standards for whale and dolphin watching. The WCAs unique partnership of stakeholders, including NGOs and scientists, whale and dolphin watch tour operators, tourism associations, and educational institutes, gives it an appreciation of the need to balance the protection of cetaceans with the practical requirements of running a tour business.

This task has taken longer than we expected; but following 18 months of revision and review, including stakeholder consultations with experts worldwide, we are confident that the recommendations in this guide will be adopted widely by the travel industry. As part of our commitment, Club Med will be promoting use of the guidelines across our supply chain, and the guide will also be freely available through the WCA website.

We hope that you, like us, will be able to work with these guidelines to ensure that people around the world can continue to watch these incredible animals in their natural habitats, whilst supporting local communities and contributing to the protection of our oceans

Regards,

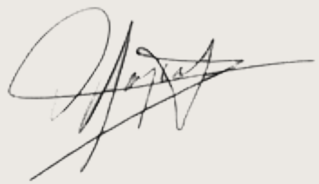

FLORIAN DUPRAT

CONTENTS

4 Key definitions
5 About this Guide
5 How to use this guide

6 **CHAPTER 1. BEST PRACTICE WHALE AND DOLPHIN WATCHING: WHAT. WHY. HOW.**

7 1.1. What is best practice?
7 1.2. Why is it important to achieve best practice?
7 1.3. How can we ensure the guidelines are effective?

9 **CHAPTER 2. BOAT-BASED WHALE AND DOLPHIN WATCHING**

10 2.1. Unacceptable practices
10 2.2. Best Practice

16 **CHAPTER 3. SWIMMING WITH WILD CETACEANS**

18 3.1. Unacceptable practices
21 3.2. Best practice
21 Operational management
23 Boat management
25 Swimmer management
25 Instructions for Swimmers

28 **CHAPTER 4. SUSTAINABILITY CRITERIA FOR BEST PRACTICE OPERATORS**

29
30 4.1. Environmental sustainability
31 4.2. Social sustainability
4.3. Economic sustainability

32 **CHAPTER 5. BEST PRACTICE WHALE AND DOLPHIN WATCHING FOR DESTINATIONS**

34 5.1. Collaborative Development of local guidelines
34 5.2. Monitoring and enforcement
35 5.3. Training
35 5.4. Licensing
35 5.5. Time-area closures and zoning
35 5.6. Multi-day excursions

36 **CHAPTER 6. THE SCIENCE BEHIND WHALE AND DOLPHIN WATCHING: IMPACTS AND BENEFITS**

37 6.1. Benefits
37 6.2. Impacts: boat-based whale and dolphin watching
39 6.3. Impacts: swimming with wild cetaceans
40 6.4. Importance of guidelines

41 **CHAPTER 7. CETACEANS IN CAPTIVITY**

42 7.1. Improving living conditions for cetaceans in captivity

CASE STUDIES

17 CASE STUDY 1. Developing a respectful swim-with operation; Ponta do Ouro, Mozambique
20 CASE STUDY 2. Customer experience in Mauritius
33 CASE STUDY 3. Vancouver Island North

INFO BOXES

14-15 INFO BOX 1. Behavioural signals
22 INFO BOX 2. The role of the guide
24 INFO BOX 3. Special guidance for calves
26 INFO BOX 4. Surface ropes

43 Acknowledgments
44 References

KEY DEFINITIONS

Avoidance	The deliberate horizontal or vertical movement of an animal away from a situation that may be causing distress or disturbance.
Best practice	The processes and procedures that are most effective in ensuring the long-term sustainability of commercial whale and dolphin watching environmentally, socially and economically. This term encompasses all aspects of cetacean tourism, including boat handling, research, education, eco-friendly use of resources, and community engagement.
Carrying capacity	The number of boats or swimmers a location can support without environmental degradation or compromising cetacean welfare or survival.
Cetacean	Collective term for the group of marine mammals in the order Cetacea, including all whales, dolphins and porpoises.
Cetacean watching	Also referred to as 'whale watching' or 'whale and dolphin watching', cetacean watching is the practice of observing whales, dolphins and porpoises (cetaceans) in their natural habitat. Cetacean watching is mostly an organised tourism activity, but it can also serve scientific and/or educational purposes.
Corralling	Term used to explain the act of taking a motor-powered boat and driving in circles around dolphins in order to encourage them to bow ride.
Disturbance	The act of disrupting normal behaviour in a negative manner, potentially leading to increased stress and avoidance.
Economic sustainability	The ability of an economy to support a defined level of economic production indefinitely.
Environmental sustainability	The ability of the environment to support a defined level of environmental quality and natural resource extraction rates indefinitely.
Food provisioning	The deliberate feeding of a wild animal to encourage an interaction or repeated interactions.
In-water encounter	A practice in which a person or group of people attempt to enter the water to float motionless within visible range of a cetacean in the wild. This technique aims to keep disturbance to an absolute minimum and encourages interactions on the animal's terms. See also Swim-with activity.
J manoeuvre	The movement of a boat that travels ahead of a cetacean or group of cetaceans in order to position swimmers directly in line and in front of the animals.
Leap-frogging	The repeated act of manoeuvring ahead of a cetacean and stopping in its path in an attempt to intercept and solicit an interaction.
Minimum standards	The minimum requirement for an operator to adhere to in order to ensure that both the welfare of the targeted cetaceans and the safety of passengers are prioritised.
NGO	Commonly referred to as NGOs, non-governmental organisations are usually non-profit and sometimes international organisations independent of governments and international governmental organisations that are active in humanitarian, educational, health care, public policy, social, human rights, environmental, and other areas to effect changes according to their objectives.
Nursery group	A group of dolphins whose members consist of 50% or more calves.
Seaside sanctuary	A managed, enclosed area of water, usually within a sheltered bay or cove in which marine mammals can be rehabilitated or retired from captivity or after injury or stranding. A seaside sanctuary provides a space with stimulating conditions allowing for natural behaviours. Animals held in seaside sanctuaries will have their health and welfare closely monitored.
Social sustainability	The ability of a social system, such as a country, family, or organisation, to function at a defined level of social well-being and harmony indefinitely.
Surface ropes	Also known as Mermaid lines or Snorkel lines, these are ropes that are attached to a boat and deployed in the water as an aid for swimmers to hold on to during a swim-with activity.
Sustainability	The ability to sustain or continue; or the avoidance of the depletion of natural resources in order to maintain an ecological balance.
Swim-with activity	A practice in which a person or group of people attempt to enter the water to swim within visible range of a cetacean. This can be in the wild or in a captive environment. See also In-water encounter.
Swim-with attempt	The point at which swimmers enter the water with the intention of having an in-water encounter with a cetacean.
Swimmer	A client of a swim-with tourism operator. Refers to all clients irrelevant of whether they are in or out of the water.
Unacceptable practices	Practices that are widely recognised as having a negative impact on cetacean welfare and/or have the potential to pose a high risk to human safety.
Whale & dolphin watching	See cetacean watching
Welfare	The health and well-being of an animal.

ABOUT THIS GUIDE

The last 50 years have seen an unprecedented interest in whales, dolphins and porpoises (cetaceans), leading to tourism activities on an expanding and global scale. Watching whales, dolphins, and porpoises in the wild is highly valuable to economies, with an estimated yearly worth of over two billion US dollars. More than 3,300 operators run trips to see cetaceans in the wild across 119 countries and territories, with over 13 million people participating every year [1]. The cetacean tourism industry also includes the viewing of captive whales, dolphins and porpoises in man-made facilities, with over 3,000 cetaceans on display in over 300 facilities around the world [2].

Whale and dolphin watching tours in the wild provide platforms for research, conservation, and environmental education, but they can also lead to disturbance, habitat displacement and even physical injury to cetaceans when poorly managed. Furthermore, public concern for cetacean welfare in captive facilities is growing, leading to the likelihood of increased demand to see cetaceans in the wild.

By providing practical, scientific and stakeholder-based guidance, Chapters 01 to 05 of this guide aim to assist wild whale and dolphin watching tour operators and destination managers wishing to achieve best practice standards. Chapter 06 outlines what scientific evidence tells us about the impacts and benefits of whale and dolphin watching.

Additionally, Chapter 07 provides information on the new area of seaside sanctuary development, and a brief introduction to current welfare standards for captive cetacean facilities, including where to find more information.

HOW TO USE THIS GUIDE

This guide covers guidance for:

- Tour operators
  - Best practice tour management
  - Sustainable practices
- Destinations
  - Management options
  - Sustainable practices

Global Best Practice Guidance for Responsible Whale and Dolphin Watching has been developed for use by commercial tour operators but should also apply to research boats and photography or filming expeditions where appropriate. It is not recommended for private boats to intentionally attempt to view or encounter cetaceans.

This guidance represents international best practice for responsible whale and dolphin watching in the wild. However, nationally applied regulations or jointly agreed guidelines that are location-specific tend to be the most appropriate in a local context. They should meet or exceed the recommendations detailed in this guidance. Nevertheless, due to unique species or location specific requirements, there may be exceptions to this rule. In such cases, whale and dolphin watch tour operators wishing to achieve best practice within the framework of local guidelines or regulations can apply for up to three exceptions to the Global Best Practice Guidance for Responsible Whale and Dolphin Watching standards through a multi-stakeholder international committee of experts facilitated by the World Cetacean Alliance (see 1.3.1 and 1.3.3).

All guidelines should be clearly displayed and available to customers at all times and actively referenced both before and during each tour.

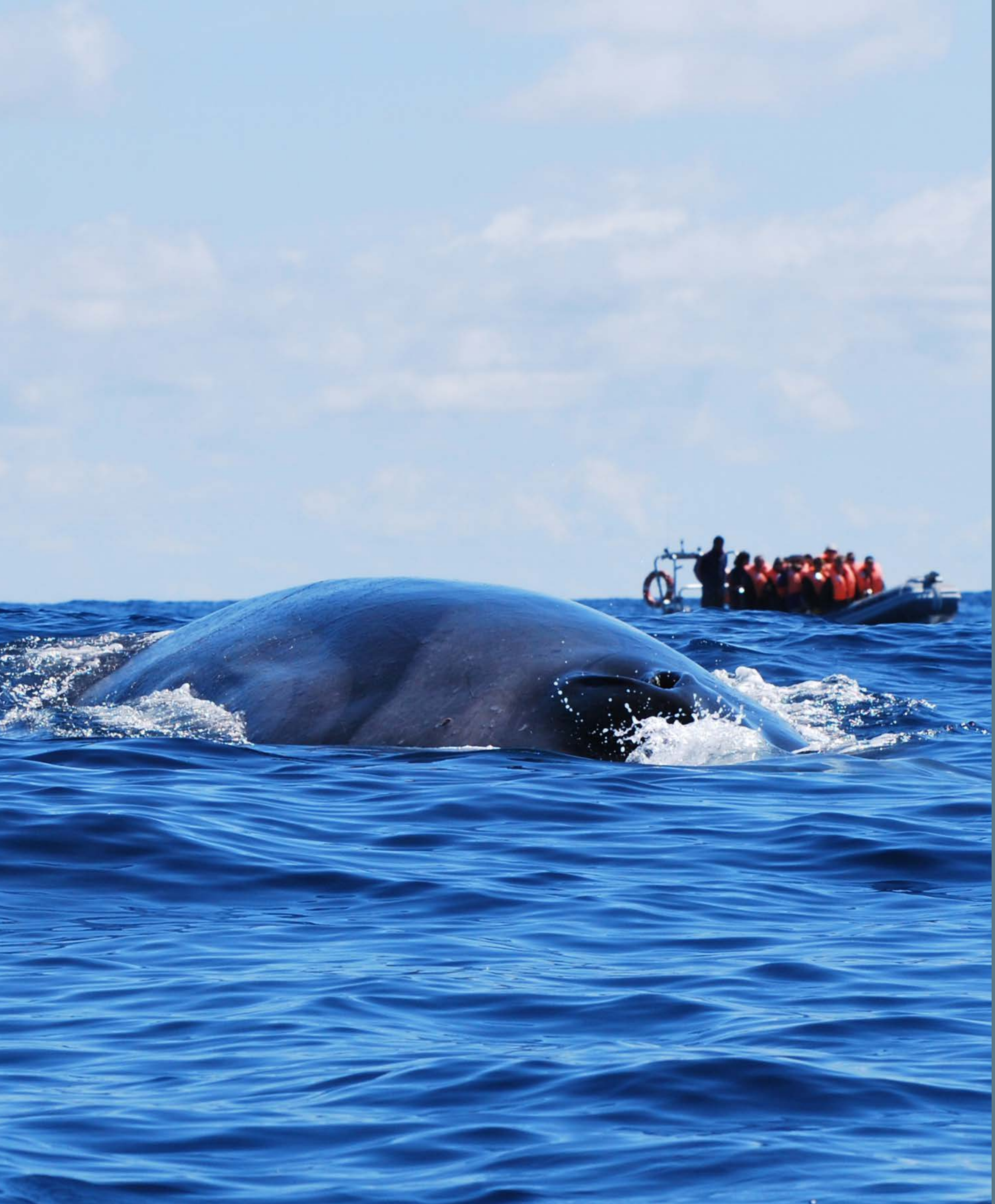

BEST PRACTICE WHALE AND DOLPHIN WATCHING: WHAT. WHY. HOW.

CHAPTER 01

© MIRANDA VAN DER LINDE / AZORES WHALE WATCHING FUTURISMO

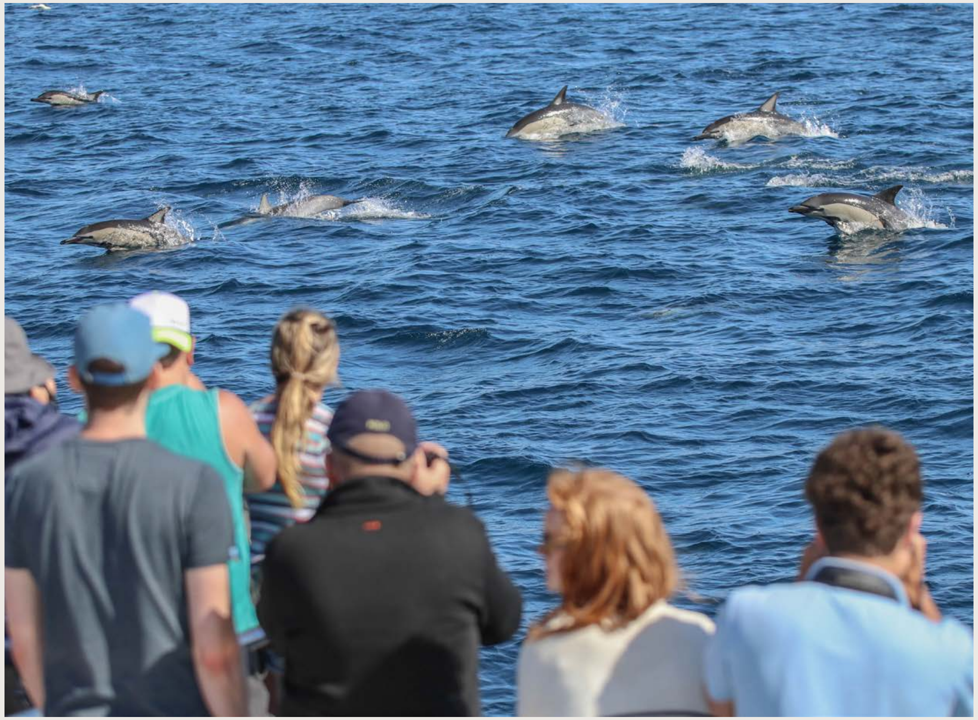

SHORT-BEAKED COMMON DOLPHINS PASS BY AT A SAFE DISTANCE FROM WCA RESPONSIBLE WHALE WATCHING OPERATOR AUCKLAND WHALE AND DOLPHIN SAFARI.
© AUCKLAND WHALE & DOLPHIN SAFARI

1.1. WHAT IS BEST PRACTICE?

Best practice for whale and dolphin watching includes guidelines, ethics and ideas that ensure cetacean watching is conducted in a manner that:

- Minimises negative impacts on cetaceans and the environment;
- Results in high levels of customer satisfaction;
- Positively benefits local communities and the environment; and
- Educates and inspires the public and the wider tourism industry.

Best practice for tour operators involves precautionary boat handling that reduces disturbance on the targeted animals to an absolute minimum, in combination with the development of a sustainability plan that will maximise the environmental, social and economic benefits delivered by the operator.

Best practice for destinations involves implementing a combination of management techniques to help minimise the potential short and long-term impacts of whale and dolphin watching on cetacean populations. Destinations should also work collaboratively towards achieving economic, social and environmental sustainability. The gold standard for sustainable whale and dolphin watching destinations are Whale Heritage Sites. Established by the World Cetacean Alliance, Whale Heritage Sites are outstanding locations where cetaceans are embraced through the cultural, economic, social, and political lives of associated communities, and where people and cetaceans coexist in an authentic and respectful way.

To find out more about Whale Heritage Sites, please visit www.whaleheritagesites.org.

1.2. WHY IS IT IMPORTANT TO ACHIEVE BEST PRACTICE?

Over the last 50 years, whale and dolphin watching has been influenced by guidelines and regulations drawn up by national and international institutions, regulatory bodies, governments, NGOs, and travel industry associations. Yet despite the progress that has been made, research highlighting the negative effects of commercial whale and dolphin watching on cetaceans in many locations continues to provide compelling evidence that standards still need to be improved and implemented more widely.

The World Cetacean Alliance is working with its Partners and the wider travel industry to implement best practice whale and dolphin watching principles and site-based management through a range of accreditation programmes, training courses, and facilitation. Global Best Practice Guidance for Responsible Whale and Dolphin Watching recommendations underpin all of our work on wild cetacean tourism. In addition, we hope that Global Best Practice Guidance for Responsible Whale and Dolphin Watching will support the work of other organisations working towards responsible whale and dolphin watching, including tour operators, governments, travel associations, and accreditation schemes.

1.3. HOW CAN WE ENSURE THE GUIDELINES ARE EFFECTIVE?

Successful guidelines must be practical, measurable and flexible. There must be a process to enable them to continually evolve that is based on the latest scientific research and industry input. Finally, there must be a mechanism for ongoing assessment to ensure compliance, with rewards for those sites and operators achieving the required standards and consequences for those sites and

operators that do not. Ultimately, the success of these guidelines will be measured on the long term viability of whale and dolphin watching, which in turn relies entirely on the continued existence of healthy cetacean populations.

The following mechanisms will ensure that these guidelines are effective wherever they are applied:

1.3.1. GUIDELINES ARE REGULARLY REVIEWED AND UPDATED

In order for these best practice guidelines to be kept up to date and inclusive of our growing knowledge they will be reviewed every two years by a multi-stakeholder international committee of experts facilitated by the World Cetacean Alliance. Each review will incorporate the latest scientific research and feedback from whale and dolphin watch tour operators on practical application.

1.3.2. GUIDELINES SUPPORT NATIONAL AND INTERNATIONAL STANDARDS

The Global Best Practice Guidance for Whale and Dolphin Watching will support pre-existing national and international standards, and form the basis for accrediting organisations that seek to audit wild whale and dolphin watching activities and revise voluntary codes of conduct and national regulation.

1.3.3. EXCEPTIONS ALLOW FOR FLEXIBILITY

Location or species specific variations of the global guidelines will be assessed on a case by case basis by a multi-stakeholder international committee of experts facilitated by the World Cetacean Alliance. A maximum of three exceptions will be granted for each applicant.

1.3.4. AUDITS ENSURE STANDARDS ARE BEING REACHED AND MAINTAINED

The guidelines are developed in a way that facilitates a simple audit process. The World Cetacean Alliance uses a range of assessments and audits involving both trained staff and independent auditors to ensure that all WCA accredited responsible whale and dolphin watching tours meet the global guidelines required for best practice.

1.3.5. GUIDELINES ARE PRACTICAL

During the development of these guidelines, careful consideration was given to the practicalities of implementing them. Whale and dolphin watch tour operators contributed extensively through a series of consultations. The result is a set of guidelines that are achievable and realistic for commercial businesses, whilst setting a high standard for the welfare of cetaceans based on current research.

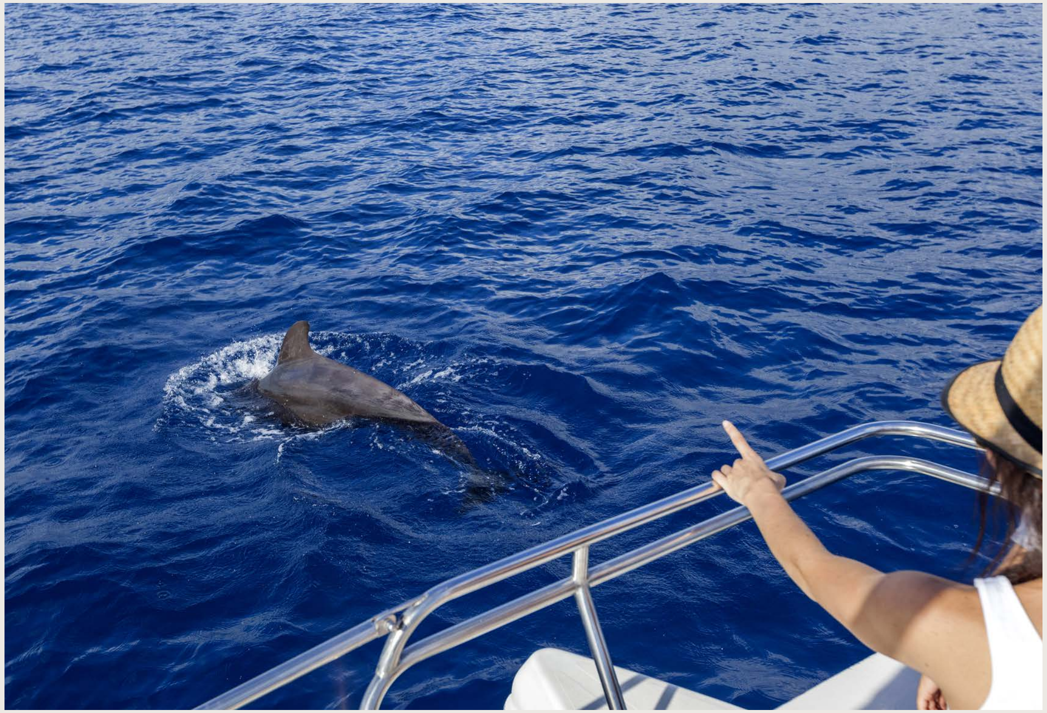

A BOTTLENOSE DOLPHIN BOW RIDES IN FRONT OF A BOAT WHILE A TOURIST ENJOYS THE VIEW WITH WCA RESPONSIBLE WHALE WATCHING PARTNER WHITE TENERIFE.
© WHITE TENERIFE

BOAT-BASED WHALE AND DOLPHIN WATCHING

CHAPTER 02

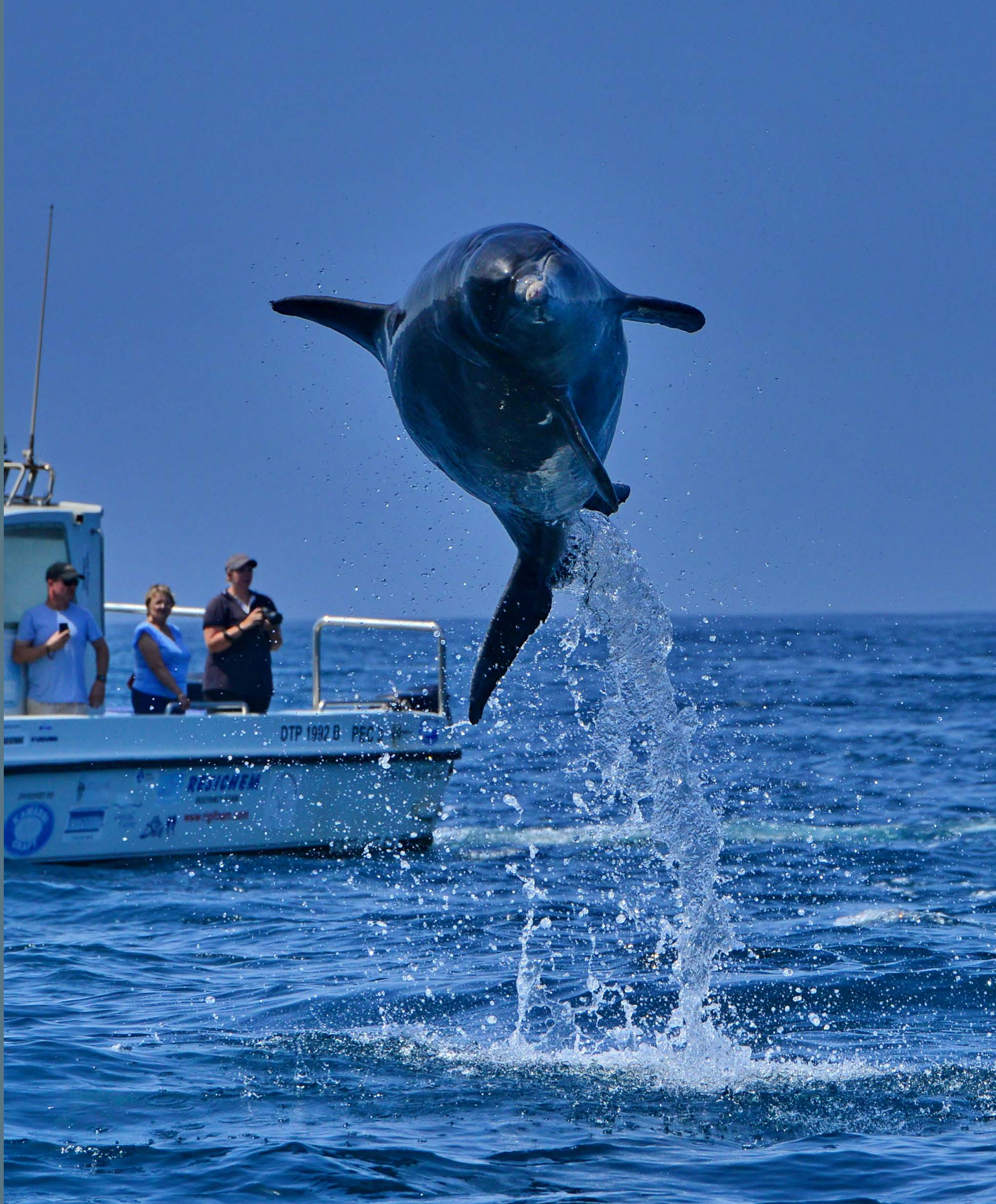

© LLOYD EDWARDS/
RAGGY CHARTERS

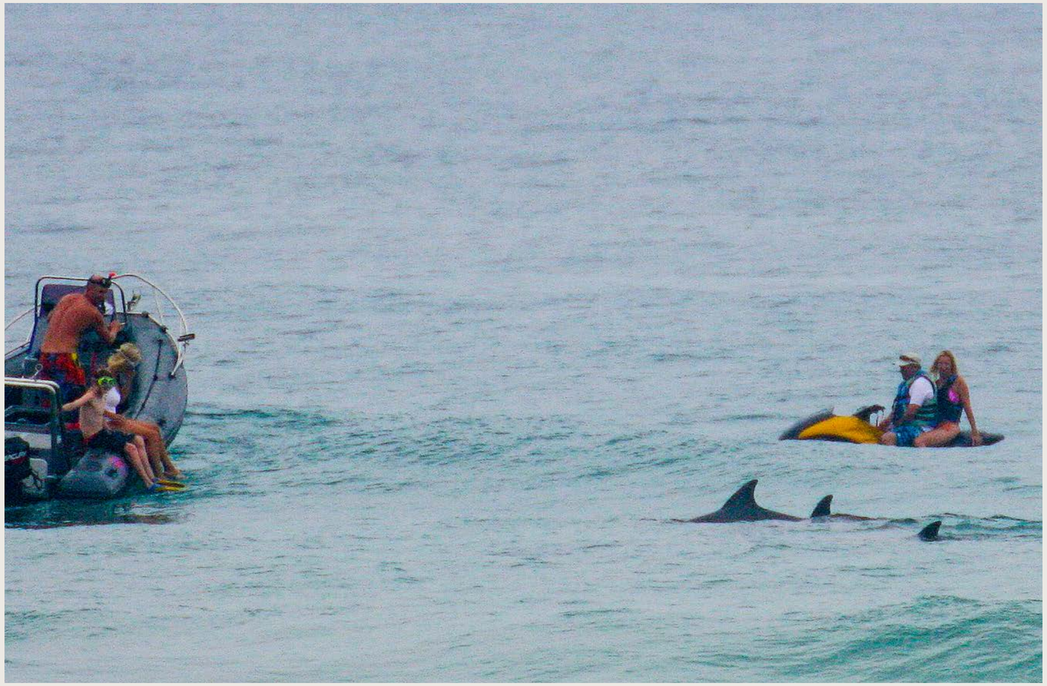

INDO-PACIFIC BOTTLENOSE DOLPHINS BEING CHASED BY RECREATIONAL BOATS AND JET SKIS IN PONTA DO OURO, MOZAMBIQUE.
©ANGIE GULLAN/ DOLPHIN ENCOUNTOURS

2.1. UNACCEPTABLE PRACTICES

2.1.1. No deliberate chasing of cetaceans by boats. Boats must ensure that cetaceans have right of way, either choosing to engage with or to ignore the presence of the boat.

2.1.2. No rubbish or food shall be thrown overboard.

2.1.3. No person shall make excessive loud, disturbing or continuous noise audible to a cetacean. Whales, dolphins and porpoises are highly sensitive to sounds. Loud or disturbing noises can result in cetaceans moving away from the area. Noises should not be used to attract cetaceans.

2.1.4. No person shall use motorised swimming aids for cetacean watching or swimming. No personal motorised boat that is designed to be operated by sitting, standing or kneeling on rather than inside the boat (e.g. jet skis) should be used for watching cetaceans.

2.1.5. The practice of leap-frogging is prohibited (leap-frogging is defined as the repeated act of manoeuvring ahead of a cetacean and stopping in its path in an attempt to intercept and solicit an interaction).

2.1.6. Operators should not enter restricted zones or areas protected from boats or swimmers as designated by local, national, or international law, conventions or agreements.

2.1.7. No person or boat shall cause any cetacean to become separated from a group or cause any members of such a group to be scattered.

2.1.8. Food provisioning of wild cetaceans should be strictly prohibited.

2.1.9. The practice of corralling is prohibited (corralling is the term used to explain the act of taking a motor-powered boat and driving in circles around dolphins in order to encourage them to bow ride.).

2.2. BEST PRACTICE

Whale and dolphin watch tour operators should meet or exceed the standards required by locally agreed guidelines or national regulations at all times. These guidelines or regulations should be communicated to all customers and made visible and accessible to customers throughout the duration of all tours.

2.2.1. Customers should be well briefed in advance, using materials that enable multiple language users to gain an understanding of:

- The potential impacts of the tour on cetaceans and the marine environment;
- The importance of guidelines and how they are adhered to;
- A health and safety briefing; and
- An understanding of what to expect from all aspects of the activity. Emphasis should be on managing expectations (see INFO BOX 2).

2.2.2. Customers must be provided with educational information on the threats to the animals and environment they are encountering.

2.2.3. Operators must ensure that each boat has a knowledgeable guide on board (see INFO BOX 2).

UNACCEPTABLE PRACTICES

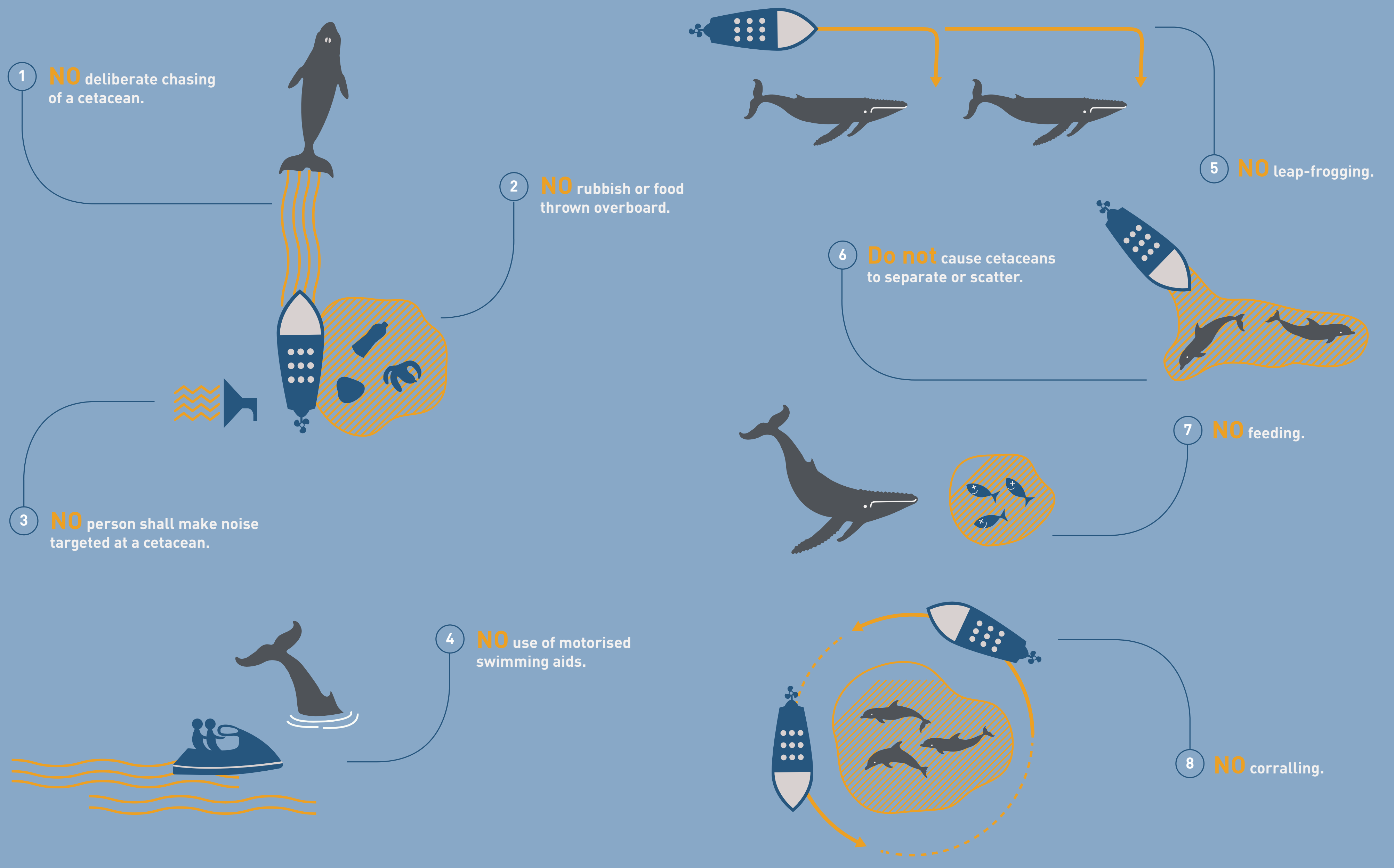

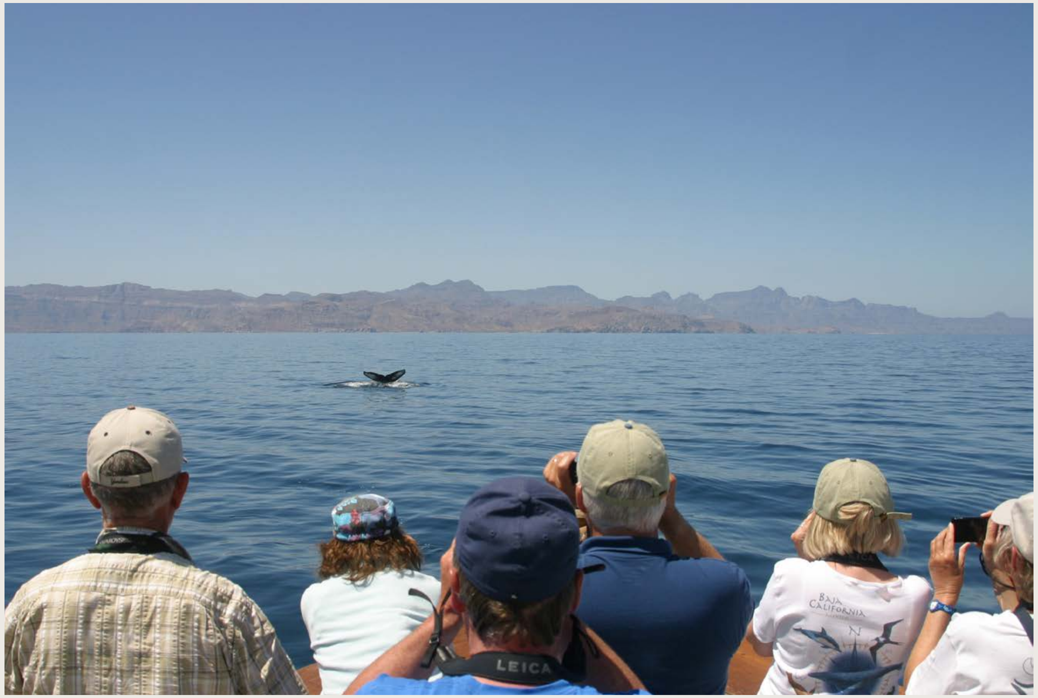

A HUMPBACK WHALE SHOWS ITS UNIQUE FLUKE PATTERNS TO A GROUP OF TOURISTS OFF THE BAJA CALIFORNIA PENINSULA, MEXICO.
© DYLAN WALKER

2.2.4. Cetaceans always have right of way. Boat skippers/captains should be constantly aware of any animal's movements within the vicinity to ensure the boat does not restrict those movements.

2.2.5. Cetaceans should always be approached from the side and slightly behind, with the boat moving in parallel. Cetaceans should never be approached directly from behind or in front.

2.2.6. Once within 300 metres of a cetacean, boat speed should be reduced to a no wake speed, avoiding gear changes and any sudden changes of speed or direction within this vicinity, except in cases of emergency.

2.2.7. Boats should not approach a whale closer than 100 metres and should not approach a dolphin or porpoise closer than 50 metres.

2.2.8. Boats must switch off echo sounders within 300 metres of a cetacean, if it is safe to do so.

2.2.9. Do not approach closer than 300 metres to a lone cetacean calf.

2.2.10. If a cetacean approaches closer than 100m to a boat, engines should be put in neutral. The boat should remain in neutral and not leave the sighting area before clearly seeing the cetacean on the surface at a minimum distance of 50 metres from the boat. The exception to this is if dolphins or porpoises approach to bow ride then 2.2.18 must be adhered to.

2.2.11. No more than three boats should be between 300 metres and the minimum approach distance of a cetacean at any one time.

2.2.12. If a fourth boat arrives, this boat should first attempt to locate another individual or group of cetaceans to view. If this is not possible, the fourth boat must wait at least 300m from the closest cetacean and all boats should reduce their viewing time to 10 minutes to reduce cumulative pressure on the animals. If the first three boats have been there for 10 minutes or more when the fourth boat arrives they must halt their activity immediately, move away and allow the fourth boat to move into position.

2.2.13. If the number of boats within 300 metres of a cetacean remains three or less, viewing time should be kept to a maximum of 30 minutes per boat.

2.2.14. When more than one boat is within 300 metres of a cetacean, all boats must stay on the same side of the cetacean so that movement is less restricted. Special care must be taken to ensure that cetaceans are not trapped between boats and other physical barriers such as islands.

2.2.15. If a cetacean shows avoidance behaviour, it must not be pursued (see INFO BOX 1).

2.2.16. Cetaceans should not be approached within 300 metres if resting or nursing behaviour is evident (see INFO BOX 1).

2.2.17. Extra caution needs to be taken by the operator when calves are present. These are especially vulnerable and sensitive individuals and should therefore be approached with increased care and sensitivity (see INFO BOX 3).

2.2.18. If dolphins or porpoises bow-ride alongside the boat, the boat should remain at a constant speed with no sudden changes in direction.

2.2.19. The operator should not return to the same cetaceans during the same trip to sea.

2.2.20. The operator must attempt to visit different cetaceans on each trip to sea during one day.

BEST PRACTICE

1 Tourists should be well briefed in advance on the potential impacts of the tour on cetaceans.

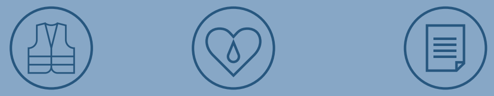

2 Operators must ensure that each vessel has a knowledgeable guide on board.

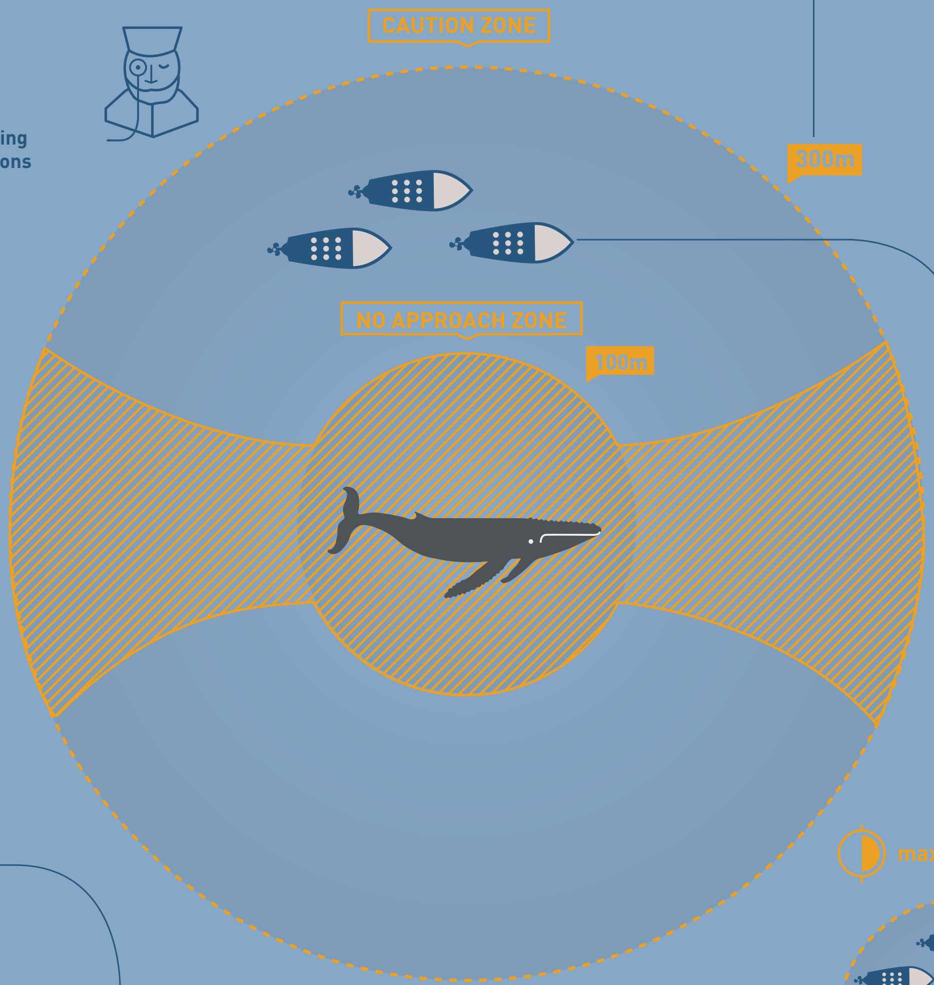

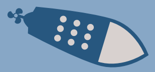

3 The vessel should approach from a direction that is parallel and slightly to the rear of the cetacean.

4 If dolphins bow-ride, the vessel should remain at a constant speed with no sudden change in direction.

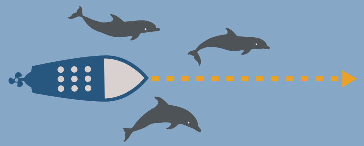

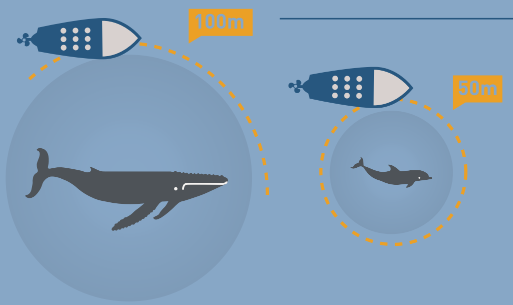

5 Vessels should not approach a whale closer than 100m and a dolphin closer than 50m.

6 Once within 300m of a cetacean, vessels should:

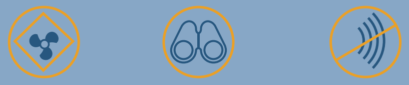

7 Stay at 300m if a lone calf is present or resting or nursing behaviour is evident.

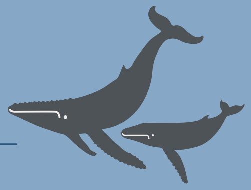

8 No more than three vessels should be between 300m and the minimum approach distance.

9 All vessels must ensure they stay on the same side as one another.

10

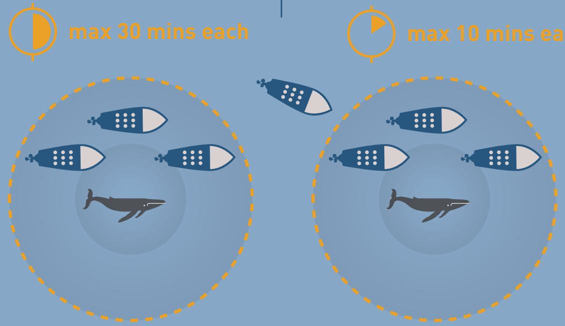

INFO BOX 01

BEHAVIOURAL SIGNALS

Being able to understand and interpret behavioural signals of cetaceans is a vital part of conducting responsible whale and dolphin watching tours. Cetaceans are intelligent and dynamic and they display a vast range of behaviours with different meanings, many of which are still unknown to us. Cetaceans vary their vocal and physical behaviours depending on the activity they are engaged in. Learning to recognise these behavioural signals ensures that an operator acts in a way that is sensitive to the targeted animals' needs and moods, minimising disturbance of cetaceans and any risks to customer safety.

Behavioural signals are often subtle and may vary between species and locations, and so it is important for operators to spend time learning the unique characteristics of the cetaceans in their location.

1

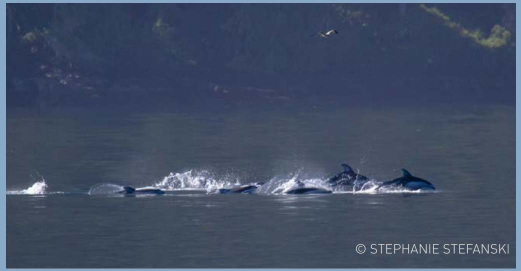

© STEPHANIE STEFANSKI

2

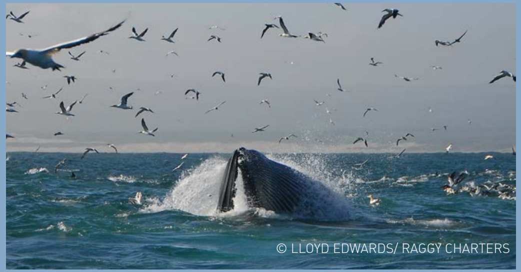

© LLOYD EDWARDS/ RAGGY CHARTERS

3

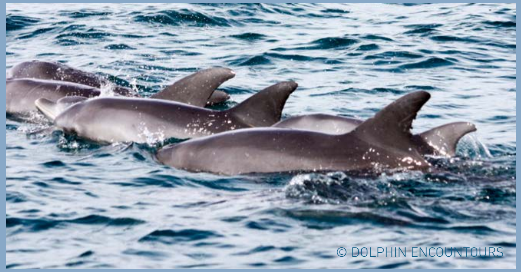

© DOLPHIN ENCOUNTOURS

4

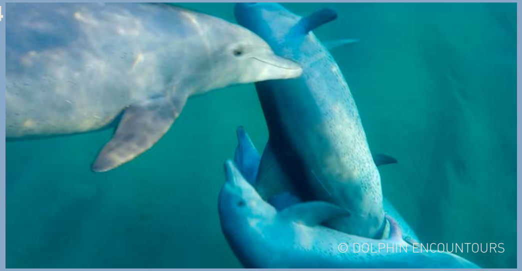

© DOLPHIN ENCOUNTOURS

The following is a non-exhaustive list of behaviours that an operator should be able to identify from a boat, or in the water during a swim-with activity.

BEHAVIOURAL STATE	DESCRIPTION
1 TRAVELLING	Consistent and directional movement, making noticeable headway along a specific compass bearing, with short, relatively constant dive intervals.
2 FEEDING	Perusal, capture and consumption of prey, as defined by observations of two or more of the following: erratic movements at the surface; multi-directional diving; coordinated deep diving; fish chasing; rapid circle swimming; prey leaping; seabirds circling or diving above cetaceans, or large predatory fish hunting alongside cetaceans.
3 RESTING	Low activity level, with surfacing slow and more predictable than those observed in other behavioural states. Tight groups (less than one body length between individuals), with little evidence of forward propulsion.
4 SOCIALISING	Chasing, copulating, petting, rubbing, playing and any other physical contact between individuals. Aerial behaviours such as breaching or tail slapping may be frequently observed.
MILLING	Non-directional movement. Frequent changes in bearing preventing dolphins from making noticeable headway in any specific direction. Individuals surfacing facing various directions.

INFO BOX 01 CONT...

DOLPHINS:
The following signals may indicate aggressive behaviour:

- Jaw clapping;
- Open jaw;
- Tail slapping;
- Fast directional swimming towards swimmers;
- Dolphin creates an 'S' shaped posture with its body.

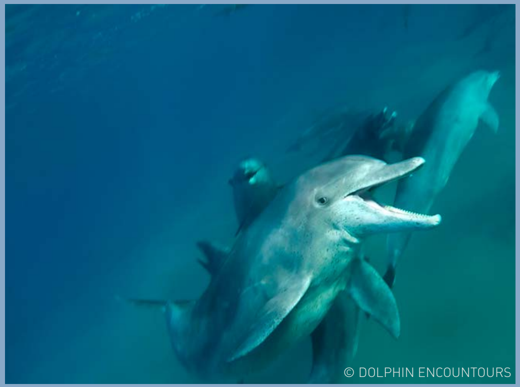

WHALES:
The following behaviours, in particular, can pose a physical risk to swimmers:

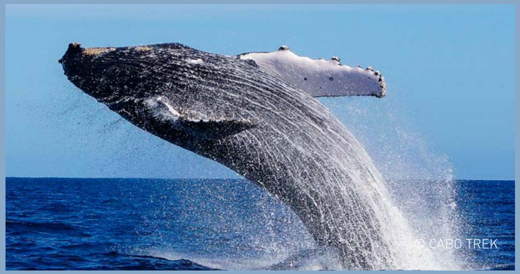

BREACHING

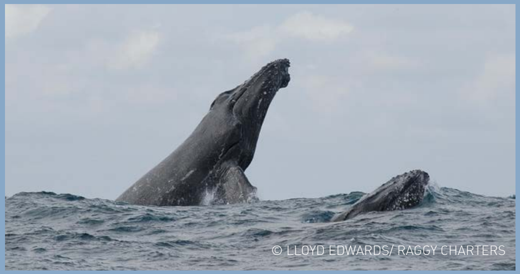

HEAD SLAPPING

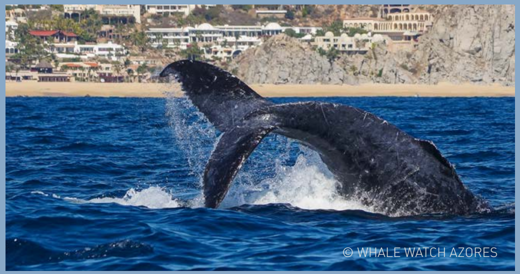

LOB-TAILING

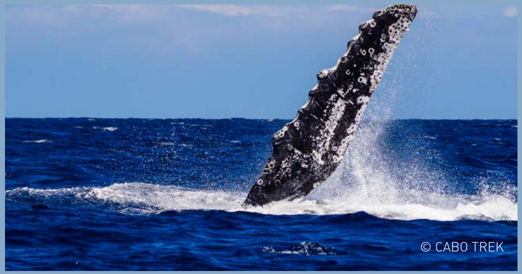

FLIPPER SLAPPING

The following signals may indicate disturbance or avoidance behaviour:

- Changes in swimming speed and direction, either horizontally or vertically (to avoid a boat or swimmers);
- Changes in breathing/diving patterns;
- Stopping or changing activity patterns;
- Changes in group size or cohesion;
- Changes in acoustic behaviour;
- Tail or flipper slapping and trumpet blows;
- Increased time spent diving compared to time spent at the surface;
- Mothers shielding their young.

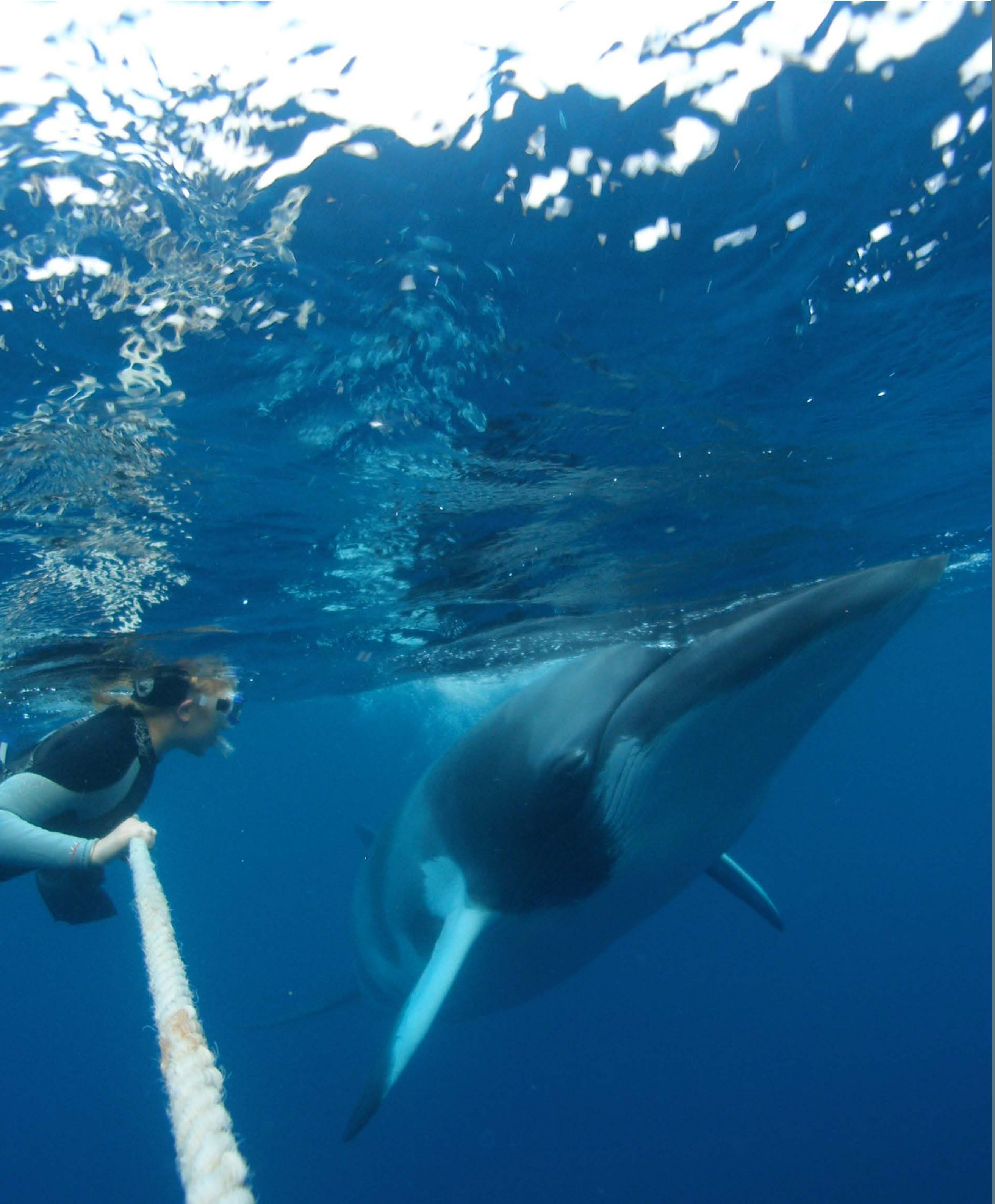

SWIMMING WITH WILD CETACEANS

CHAPTER 03

Swimming with wild cetaceans is arguably a more invasive form of tourism than observing from boat, land or air. This is partly because boats often attempt to get closer to cetaceans to place swimmers in the water, and because the act of entering the water can disturb animals that were previously engaged in other activities.

Guidelines for swim-with activities have gained less attention and refinement than for boat-based whale and dolphin watching activities. This may be due to the fact that many swim-with activities have developed relatively recently, and because there are a lack of long term studies to help support the development of responsible guidelines.

© JOHN RUMNEY/ EYE TO EYE MARINE ENCOUNTERS

CASE STUDY 01

DEVELOPING A RESPECTFUL SWIM-WITH OPERATION PONTA DO OURO, MOZAMBIQUE.

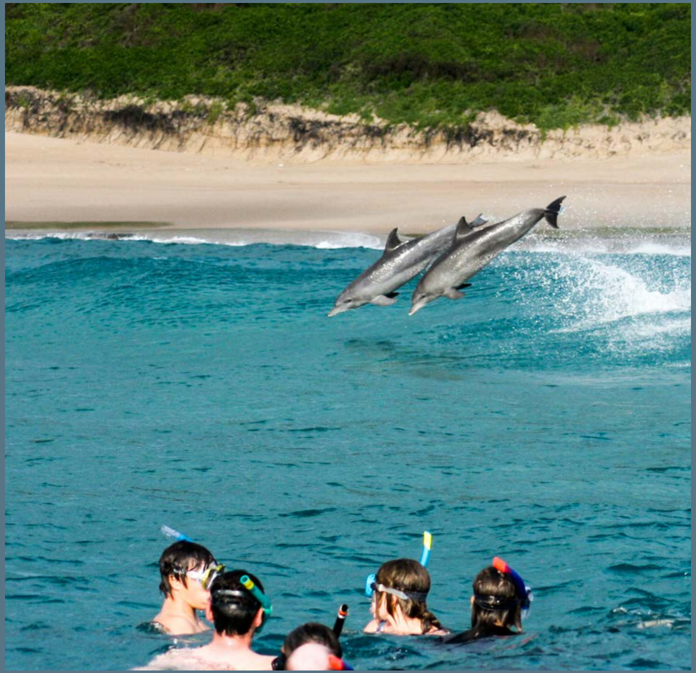

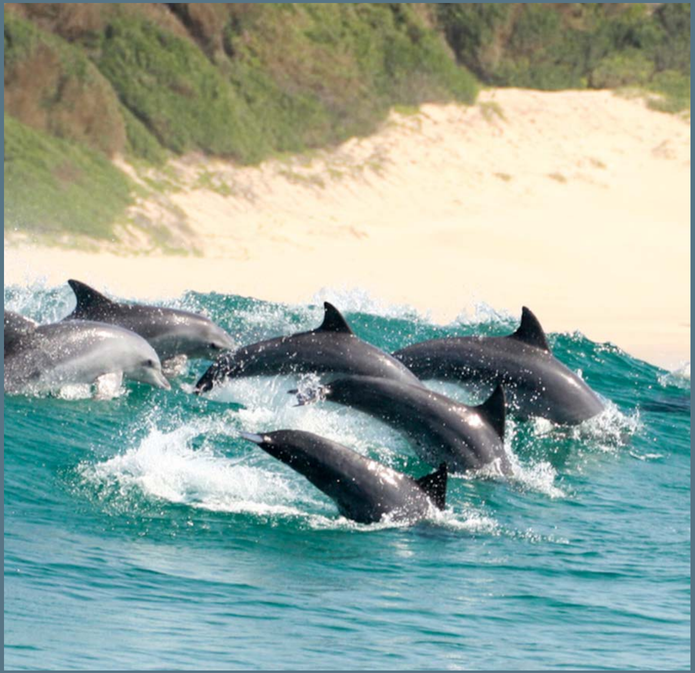

CUSTOMERS IN PONTA DO OURO, MOZAMBIQUE, ENJOY RESPECTFUL SWIM-WITH ACTIVITIES WITH WCA RESPONSIBLE WHALE WATCHING OPERATOR DOLPHIN ENCOUNTOURS. © DOLPHIN ENCOUNTOURS

Swim-with activities began in the sheltered bay of Ponta do Ouro, Mozambique, in the mid 1990's when Dolphin Encountours founder Angie Gullan stumbled across a resident pod of Indo-Pacific bottlenose dolphins.

Shortly after this operation was established, an area and species specific code of conduct was developed through consultations with experts from both the scientific and tourism communities. This code of conduct was created with the aim of establishing an activity that is safe for swimmers whilst also being sensitive to dolphins. It became apparent that a vital component of this code of conduct was allowing time prior to swim-with attempts to observe and interpret the dolphins'moods and needs through their behaviours. Many years of observations have meant that the Dolphin Encountours crew have gained an in depth understanding of these behaviours, and are able to identify subtle signals that ensure that all encounters are on the animals' terms.

Ongoing science has also been fundamental in the development of this responsible operation, with tours providing a regular platform for researchers to collect valuable data such as behavioural observations and photographic identification images. The data is then incorporated into long-term datasets used to monitor dolphin populations.

Through collaboration and sensitive management, Dolphin Encountours have developed a swim-with programme focused on responsible practices, scientific research, and education. Dolphin Encountours, in partnership with their not-for-profit organisation, DolphinCare-Africa, also recognise the need for flexibility and continued development and adaptation of their code of conduct as new scientific knowledge emerges and tourism pressure continues to rise.

3.1. UNACCEPTABLE PRACTICES

3.1.1. No touch, no dive and no chase policy ensures that cetaceans have right of way, either choosing to engage with or to ignore the presence of swimmers.

3.1.2. No swimming when there is a whale calf present (see INFO BOX 3).

3.1.3. No swimming with a nursery group of dolphins (a group of dolphins consisting of 50% or more calves) (see INFO BOX 3).

3.1.4. No person shall use Self Contained Underwater Breathing Apparatus (SCUBA) for diving or swimming with cetaceans.

3.1.5. No person shall use artificial light sources around cetaceans, including flash photography.

3.1.6. No towing of swimmers during a swim-with activity.

3.1.7. No foreign objects shall be taken into the water, with the exception of cameras and floatation devices. This includes selfie sticks, which may cause injury to cetaceans or other swimmers.

3.1.8. Refer to Unacceptable practices for boat-based whale and dolphin watching (2.1.) which also apply.

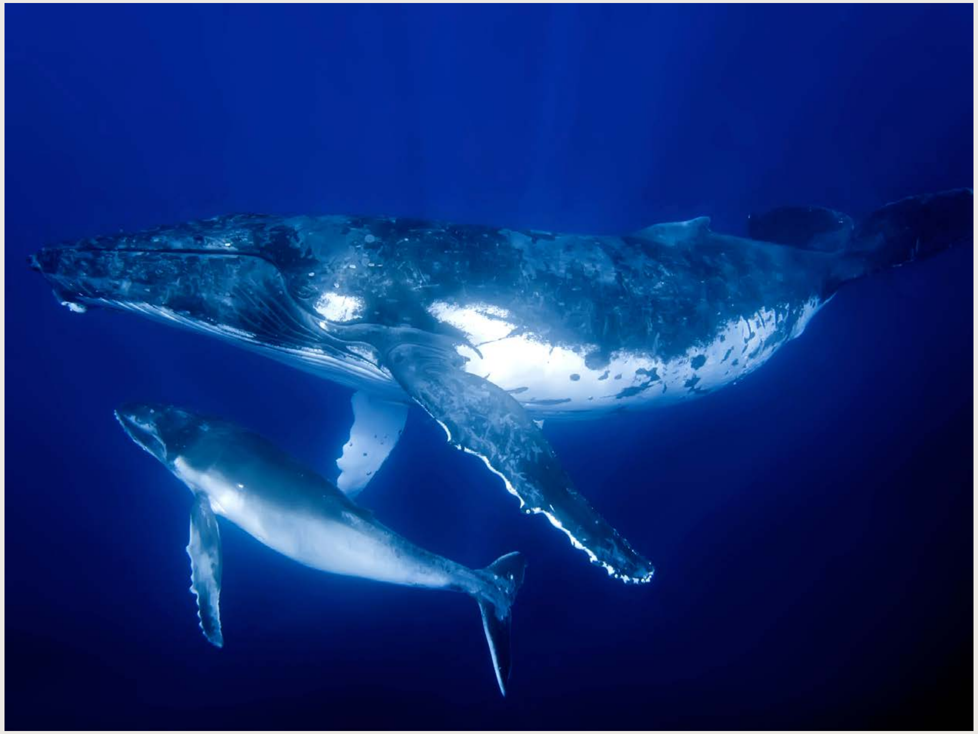

HUMPBACK WHALE ADULT AND CALF STAY CLOSE TOGETHER DURING THE EARLY STAGE OF THE CALF'S LIFE.

UNNACCEPTABLE PRACTICES

1. **NO** touch, no dive and no chase policy. Ensure that cetaceans have right of way.

2. **NO** swimming when there is a whale calf present.

3. **NO** swimming with a nursery group of dolphins (a group made up of 50% calves or more).

4. **NO** person shall use Self Contained Underwater Breathing Apparatus (SCUBA) for diving or swimming with cetaceans.

5. **NO** person shall use artificial light sources around cetaceans, including flash photography.

6. **NO** towing of swimmers during a swim-with activity.

7. **NO** foreign objects shall be taken into the water, with the exception of cameras and floatation devices.

8. **NO** water entry if feeding or resting are evident.

CASE STUDY 02

UNACCEPTABLE PRACTICE:
A CUSTOMER EXPERIENCE IN MAURITIUS

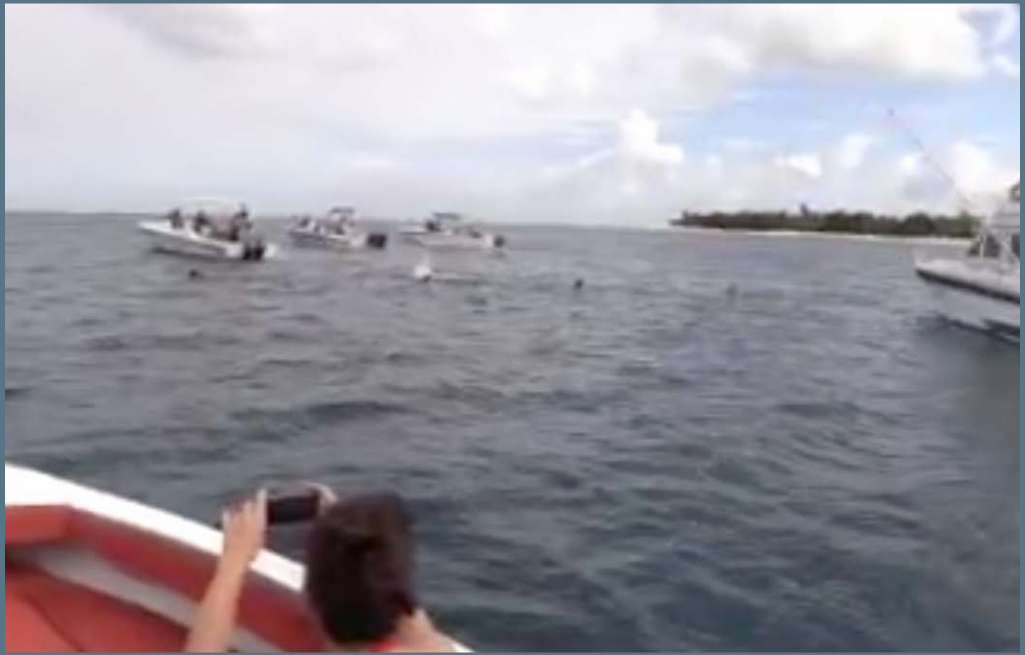

VIDEO STILL: MULTIPLE BOATS OFFERING SWIM-WITH ACTIVITIES CROWD AROUND A POD OF DOLPHINS IN MAURITIUS. GUIDELINES WERE NOT FOLLOWED, LEADING TO POOR CUSTOMER EXPERIENCES AND A POTENTIAL RISK TO CUSTOMER SAFETY. © CLIVE MARTIN.

This case study outlines a tourist's account of a swim-with encounter that was taken in Mauritius in October 2016. Parts of the encounter were recorded to document the worrying practices being observed. Several examples of unacceptable swim-with operator practices were witnessed during the tour, which were considered dangerous for both dolphins and swimmers.

The practices witnessed included:

- Too many boats around the dolphins;
- Boats too close to swimmers and maneuvering dangerously;
- Swimmers told to jump into the water, often on top of the dolphins;
- Boats chasing dolphins when dolphins tried to move away.

The below account is from the tourist that took the video footage:

"When the boat got into position ahead of the dolphins the crew shouted to the swimmers to jump, and in the case of my boat they actually pushed clients into the water!

One female swimmer immediately got into swimming difficulties and I was able to attract the 'crew swimmer' to give her assistance.

Initially and because we had arrived early, only six boats participated in the encounter, but as I videoed the tour, this quickly elevated to over 45 boats. All of the boats were "speed boats", powered by powerful outboard engines.

VIDEO STILL: A CUSTOMER JUMPS OFF A MOVING BOAT IN FRONT OF A GROUP OF DOLPHINS (IN THE DISTANCE). © CLIVE MARTIN.

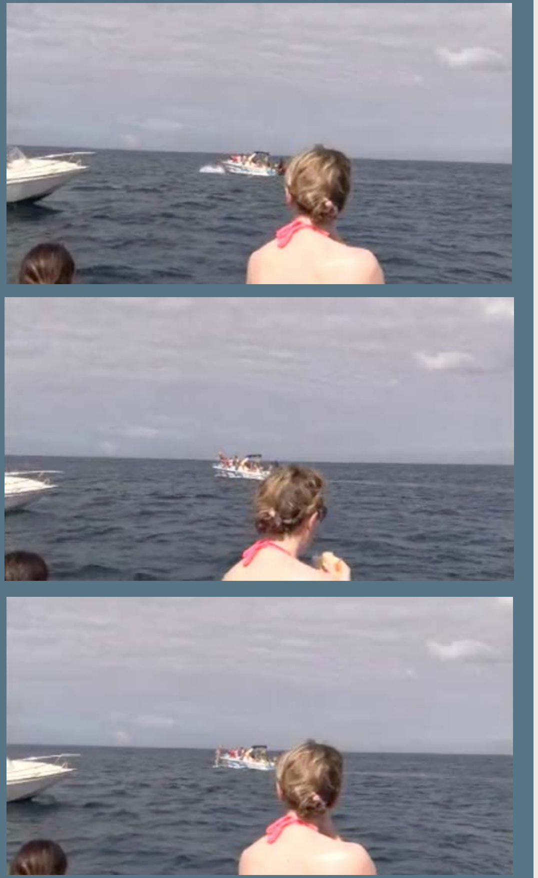

Danger existed as each of the boats maneuvered to get ahead of the dolphins and to discharge their clients into the sea. This was often accomplished at high speed with swimmers in very close proximity: Forty five boats with up to 10 swimmers in each boat....mayhem!"

- Clive Martin, UK, 2017.

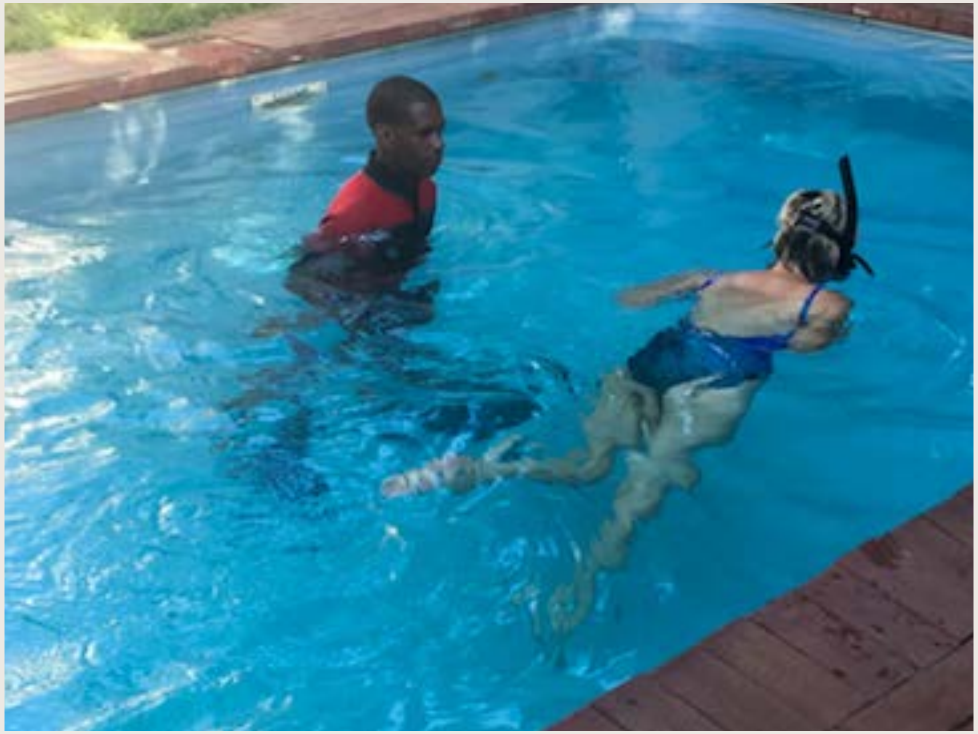

A TOURIST COMPLETES A PRACTICE SWIM BEFORE HEADING OUT ON THE WATER WITH WCA RESPONSIBLE WHALE WATCHING PARTNER DOLPHIN ENCOUNTOURS.
© DOLPHIN ENCOUNTOURS

3.2. BEST PRACTICE OPERATIONAL MANAGEMENT

3.2.1. Operators should have at least one guide on each boat with excellent knowledge of the behaviours of all targeted cetaceans. They should be able to recognise behaviours from the boat and be able to act accordingly (see INFO BOX 1).

.

3.2.2. Operators must have one crew member on board that is fully trained in aquatic rescue and able to recognise swimmers in distress and therefore act accordingly. It is recommended that this crew member is additional to the swim-with guide and can therefore stay on board the boat to monitor the situation. Each boat must carry an approved first aid kit and at least one member of the crew on board must have a current first aid certificate.

3.2.3. Operators must ensure that swimmers disclose any health conditions not conducive to a swim-with activity and should not permit those swimmers to participate.

3.2.4. Operators must ensure that all swimmers have a good level of fitness to enable them to swim for a sustained amount of time in open ocean conditions and to enter and exit the water by the method employed by the operator.

3.2.5. Operators must complete a practice swim with swimmers before any attempt to swim with a wild cetacean. It is recommended, where possible, that this is conducted in the same environment that the swim-with activity will take place. This is to:

- Enable swimmers to practice the procedures that will occur when the activity begins;
- Allow the guide to assess swimming ability.

3.2.6. Customers should be well briefed in advance, using materials that enable a good understanding in multiple languages, of:

- The potential impacts of the activity on cetaceans;
- The protocol and guidelines to follow in the water, including warning that any non-compliance with guidelines will result in termination of the activity by that individual. The guide and/or skipper/captain has final say;
- The health and safety briefing;
- What to expect in all aspects of the activity.

Emphasis should be on treating cetaceans with the utmost respect and managing expectations by explaining that there is no guarantee of an encounter.

3.2.7. Customers must be provided with information on the threats to cetaceans and their environment.

3.2.8. Swimmers must be given an explanation of cetacean behaviours and how to recognise specific behavioural signals, particularly relating to avoidance, disturbance and aggression (see INFO BOX 1).

3.2.9. There should be no water entry if the following behaviours are evident:

- Feeding
- Resting
- Avoidance

INFO BOX 02

THE ROLE OF THE GUIDE

Guides play a vital role on any responsible whale and dolphin watching trip. A knowledgeable guide has the ability to positively influence the success of a trip, irrespective of marine cetacean sightings.

A guide should be able to:

- Manage customer expectations at all times on the trip;

- Have excellent knowledge of cetacean behaviours and be able to communicate those behaviours;

- Explain the need for careful boat handling and/or responsible swimming.

- Educate passengers on current threats to cetaceans and their environment, describe some simple options customers can undertake to help protect cetaceans, and provide further information to encourage customers to undertake some of those options.

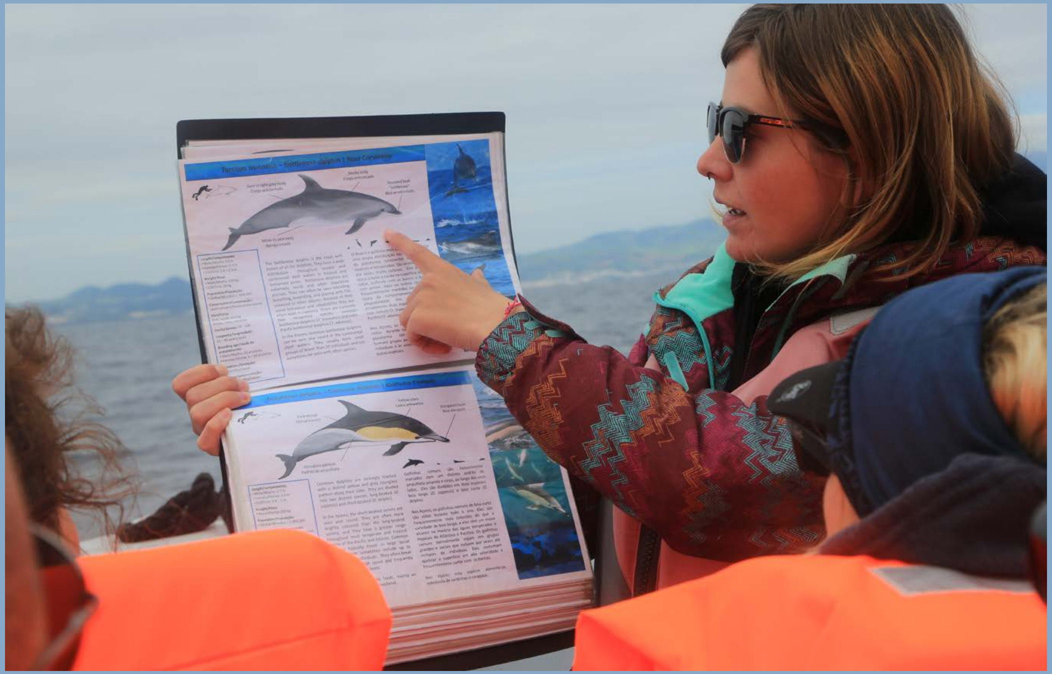

A TRAINED WHALE WATCHING GUIDE PROVIDES CUSTOMERS WITH INFORMATION ABOUT THE BIOLOGY OF THE CETACEAN SPECIES THAT MAY BE ENCOUNTERED DURING A TOUR WITH WCA RESPONSIBLE WHALE WATCHING OPERATOR DOLPHIN AND WHALE CONNECTION.
© DOLPHIN AND WHALE CONNECTION

MANAGING EXPECTATIONS

Achieving high levels of customer satisfaction can often put pressure on operators to deliver an experience that can live up to the high expectations of passengers. This pressure is often exacerbated by the marketing and promotional materials provided by the operator or through the wider media. Tourists often expect close encounters that may not be realistic. This pressure to meet the unrealistic expectations of customers can lead to non-compliance of guidelines and disturbance of cetaceans.

Appropriate expectations should be set by providing realistic information on: the likelihood of seeing the target species; the kinds of behaviours usually encountered; the variability in the weather; and other elements of the tour. Care should be taken to provide realistic information and appropriate images on websites, social media and advertising material, through sales staff, and by the guide throughout the tour.

It is a common misconception that meeting the high expectations of customers is better than managing those expectations carefully. Instead, tour operators adopting the mantra of 'under sell and over deliver' benefit from good customer reviews without compromising guidelines or regulations in place to protect cetaceans.

WHAT MAKES A GREAT WHALE AND DOLPHIN WATCHING GUIDE?

Key attributes:

- Personality: Honest, enthusiastic, personable, welcoming;

- Knowledge: Broad knowledge of marine wildlife gained from background learning, conferences and experience. Knowledge of local wildlife including stories and facts relating to the population of animals that will be observed. Knowledge of threats to marine wildlife and potential conservation strategies;

- Communication: Communicates with groups and individuals; appeals to different learner types, engages visitors through story-telling and other interpretive techniques, makes themselves available to answer and ask questions.

A good guide will increase customer appreciation and understanding of the animals and their environment, help ensure compliance with guidelines, and be the trusted point of communication for passengers.

A great guide will develop emotional and informative connections between passengers and the wildlife and environment that they encounter. Connections will be formed through story-telling, interactive activities, and information provision that relates the lives of cetaceans to the lives of people in order to encourage empathy and appreciation. The goal of a great guide is to inspire customers and encourage short or long term behaviour change to care for and protect our oceans.

BOAT MANAGEMENT

3.2.10. The boat should approach from a direction that is parallel and slightly to the rear of a cetacean, and never in the area directly in front of or behind a cetacean.

3.2.11. Once within 300 metres of a cetacean, boats must slow to a no wake speed. Avoid gear changes and any sudden changes of speed or direction except in cases of emergency.

3.2.12. Boats must switch off echo sounders within 300 metres of a cetacean, if it is safe to do so.

3.2.13. Only boats participating in swim-with activities may approach closer than 100 metres, if it is appropriate to do so, but no boats should approach a cetacean closer than 50 metres. When within 100 metres, boats should travel with extreme caution and at a no-wake speed.

3.2.14. No boat should be positioned up wind of, or in a position where it will drift into, the direction of travel of any cetacean when in neutral.

3.2.15. Only one boat is permitted between 100 metres and 50 metres of a cetacean.

3.2.16. If there is already a boat approaching and/or involved in a swim-with attempt with a cetacean, additional boats must stay at least 300 metres from that cetacean.

3.2.17. Do not attempt to approach a cetacean immediately after another boat has attempted a swim-with encounter. If you see a boat involved in an encounter with a cetacean, move away and attempt to find different individuals to approach. As a minimum, you must wait for at least one hour after the previous encounter has finished before attempting an encounter.

3.2.18. If a cetacean unexpectedly appears within 50 metres of the boat, the boat should be put in neutral and remain in neutral until the cetacean is clearly on the surface at a minimum distance of 50 metres from the boat.

3.2.19. When a boat stops for swimmers to enter the water, the engines must be placed in neutral and remain so until the swimmers exit the water, with the exception of an emergency.

3.2.20. When readying to place swimmers in the water, boats should not position themselves any closer than 50 metres to a cetacean whilst following the boat approach guidelines at all times (see 3.2.10. to 3.2.19.).

3.2.21. Swimmers should never be placed directly in a cetacean's line of travel or on top of the animals.

3.2.22. Once swimmers are in the water, the boat should raise the alpha flag or a location specific alternative.

3.2.23. Boats must allow enough space for swimmers and cetaceans without risk of injury, but close enough to react in an emergency.

3.2.24. Departing boats should ensure that all animals are visible on the surface at least 50 metres away from the boat before proceeding slowly at no wake speed until the boat is at least 300 metres from the nearest animal.

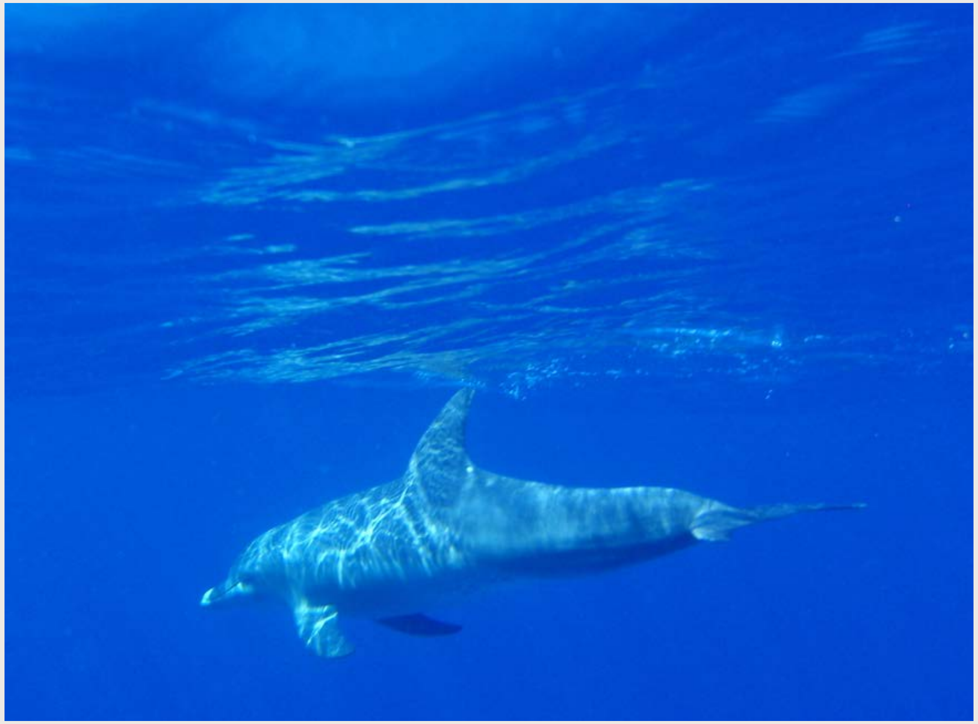

A PREFERENCE FOR COASTAL WATERS MEANS THAT INDO-PACIFIC BOTTLENOSE DOLPHINS ARE A POPULAR TARGET SPECIES FOR COMMERCIAL SWIM-WITH TOURS. © RACHEL BARBER

INFO BOX 03

SPECIAL GUIDANCE FOR CALVES

Due to a lack of long term studies, it is challenging to discern the effect that tourism pressure is having on the overall reproductive success of populations. Limited studies have found that tourism pressure may reduce calf survival (4). Cetacean calves are particularly vulnerable to external factors and during this stage of life they rely heavily on their mother for food and protection. Although the exact means in which tourism can affect calves is often unclear, it is suggested that a variety of mechanisms may, singularly or in combination, have negative impacts, including:

- Reduced resting time when nursing is most likely to occur (5);
- Increased risk of predation through mother and calf separation (4);
- Decreased calf health due to increased energy expenditure as a result of avoidance behaviours (6).

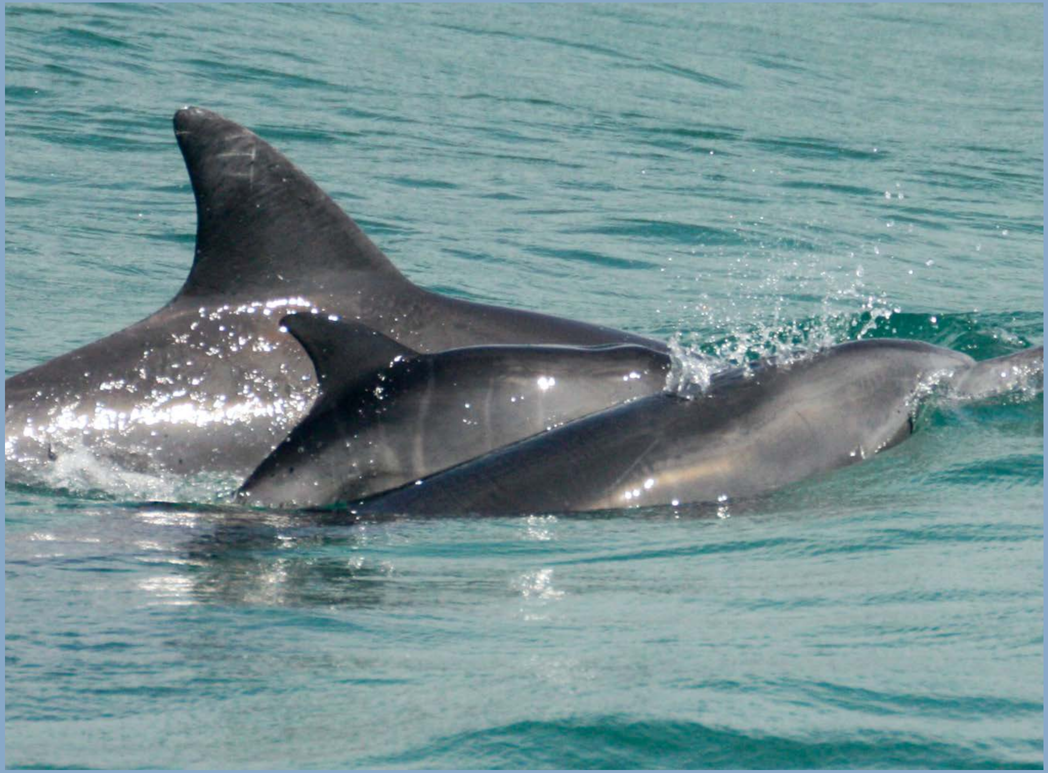

FLANKED BY TWO ADULTS, THIS INDO-PACIFIC BOTTLENOSE DOLPHIN CALF IS DISPLAYING VERTICAL WHITE MARKINGS ON ITS BODY KNOWN AS FOETAL FOLDS.
© ANGIE GULLAN/ DOLPHIN ENCOUNTOURS.

This guide recommends that lone calves should not be approached closer than 300 metres, and that swimming should not be allowed with whale calves or nursery groups of dolphins (where calves make up 50% or more of group composition).

It can be challenging to identify calves from a boat or in the water, and therefore a precautionary approach should always be taken. Characteristics that can be used to identify calves differ between species, so a good field guide is recommended. As a general rule, the following signs can be used to identify calves:

- The calf appears less than half the body length of its mother;
- The calf displays different colouration, usually a more consistent darker or lighter colour, than adults;
- In the case of dolphins and some whale species, calves may have foetal fold markings on their bodies. These markings tend to indicate calves in their first few weeks of life, though they can remain visible for several months after birth.

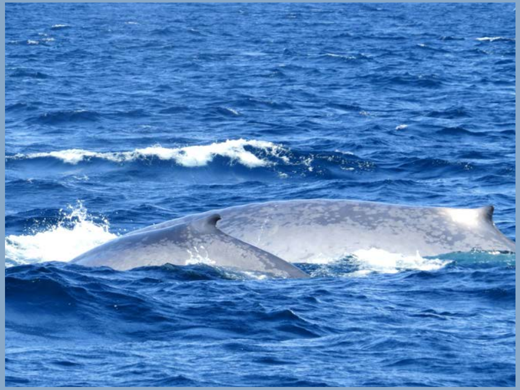

BLUE WHALE ADULT AND CALF. © ANN ELPHICK

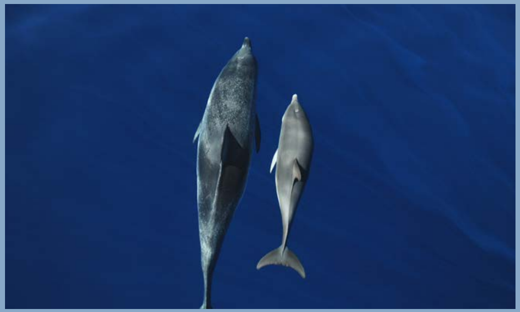

ATLANTIC SPOTTED DOLPHIN ADULT AND CALF.
© MIRANDA VAN DER LINDE/ AZORES WHALE WATCHING FUTURISMO

SWIMMER MANAGEMENT

3.2.25. Operators must limit the number of swimmers in the water with a cetacean at any one time. This is for the safety of the swimmers and to minimise disturbance to the targeted cetacean.

For swim-with wild dolphin activities: operators should permit no more than eight swimmers in the water at one time. All swimmers should enter and leave the water together to decrease disturbance.

For swim-with wild whale activities: operators should permit no more than four swimmers in the water at one time. All swimmers should enter and leave the water together to decrease disturbance.

3.2.26. Swimmers must be under constant supervision by an experienced guide and be ready to follow instructions at all times (see INFO BOX 2).

3.2.27. A surface rope should be used to aid swimmers in the water. Exceptions to the use of surface ropes apply only when conditions are unsafe (see INFO BOX 4).

3.2.28. The guide should enter the water first, assess the behavioural state of cetaceans, and deploy the surface rope (assuming conditions are safe to do so). Only when the guide has confirmed that the situation is safe for both swimmers and cetaceans, should swimmers be allowed to enter the water. The guide must have final say.

3.2.29. The guide must monitor the behaviour of the cetaceans and swimmers at all times. A swim-with attempt must be stopped if:

- A cetacean displays signs of:
  - feeding
  - nursing
  - disturbance
  - aggression
  - behaviours that pose a physical risk to swimmers (see INFO BOX 1)
- Weather conditions present a risk to swimmer safety;
- Swimmers do not comply with the guidelines (applies only to the swimmer/swimmers that do not comply);
- Another boat arrives and places swimmers in the water;
- Any Unacceptable Practice takes place.

3.2.30. In-water time must be limited to a total of 20 minutes with a cetacean. There must be no return to that cetacean during the same day even if the encounter lasts less than 20 minutes.

3.2.31. Only one successful swim-with attempt should be allowed during each trip (or each day for multi-day trips). If two failed swim-with attempts occur, where no cetacean is visible under water, all swimmers should cease swim-with attempts and exit the water (a swim-with attempt is defined as the point at which swimmers enter the water with the intention of having an in-water encounter with a cetacean).

INSTRUCTIONS FOR SWIMMERS

3.2.32. Swimmers should remove rings, piercings and any other sharp object which may cause harm to a cetacean during accidental contact.

3.2.33. Swimmers should enter the water gently, attempting to make as little splashing as possible.

3.2.34. When a surface rope is being used, swimmers must hold on to the rope at all times during an encounter provided that conditions are safe to do so. Swimmers must position themselves so as to avoid bumping into one another.

3.2.35. If not using a surface rope (see INFO BOX 4), swimmers must keep their arms by their sides, across their chest or behind their back and swim in a calm and predictable manner. Swimmers should position themselves in a line.

3.2.36. If a cetacean chooses to approach swimmers, the swimmers should remain as still as possible whilst holding on to the surface rope. If not using a surface rope, a calm and predictable swimming pattern should be maintained and swimmers should position themselves in a line.

INFO BOX 04

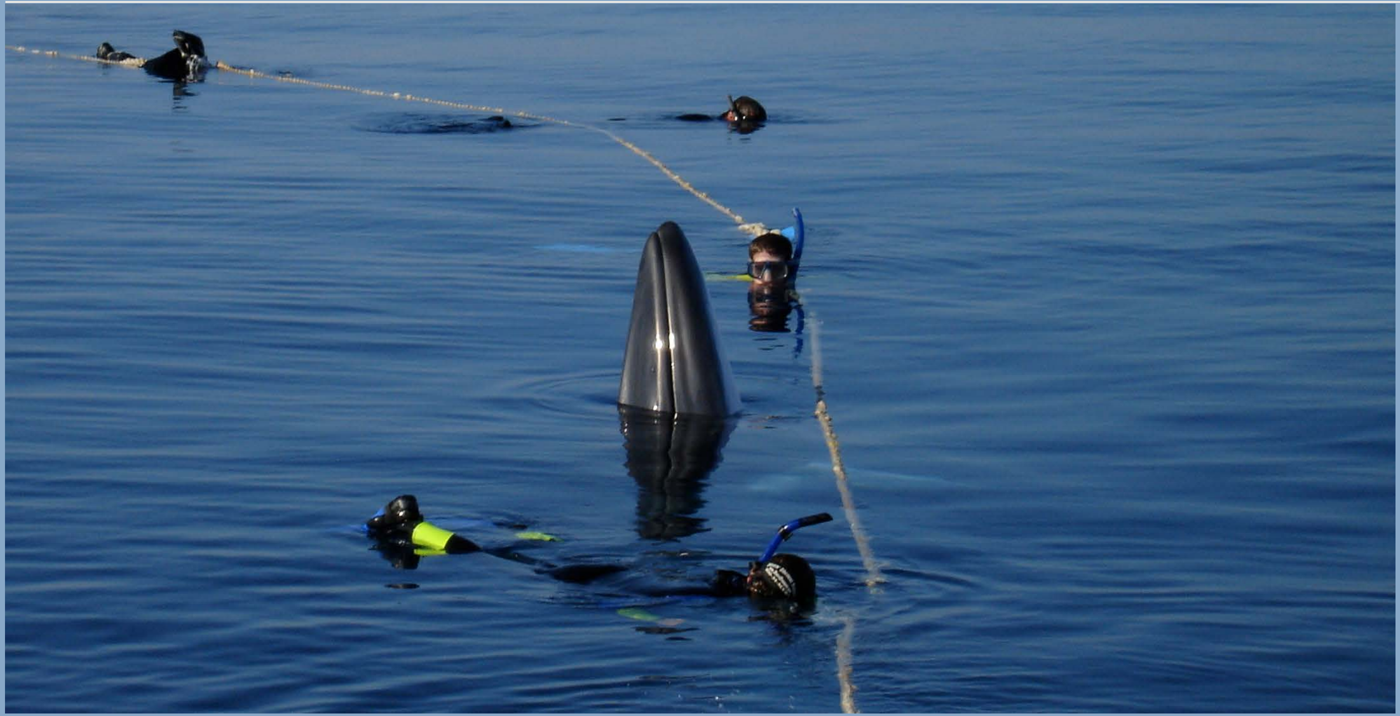

CUSTOMERS ON AUSTRALIA'S GREAT BARRIER REEF WITH WCA RESPONSIBLE WHALE WATCHING OPERATOR EYE TO EYE MARINE ENCOUNTERS HOLD ON TO A SURFACE ROPE TO CREATE A SAFE AND PREDICTABLE ENVIRONMENT FOR CURIOUS DWARF MINKE WHALES.

© JOHN RUMNEY/ EYE TO EYE MARINE ENCOUNTERS.

SURFACE ROPES

Surface ropes (also known as mermaid lines or snorkel lines) are a proven effective method of reducing disturbance during wild swim-with activities. The use of a surface rope allows swimmers to be present at the surface of the water in a calm and predictable manner, thereby reducing disturbance. There is anecdotal evidence that the predictable nature of the encounter also puts cetaceans at ease, resulting in improved experiences for customers. Research has shown that using a surface rope result in increased customer satisfaction, particularly when an explanation of the purpose of the ropes is explained (39).

Surface rope specifications vary widely and there is some debate over the characteristics of the ideal rope for different target species, including the length, thickness, number of floatation devices, and breakage points. The optimum rope characteristics for these activities requires further investigation.

The surface rope should be attached to the vessel and positioned in a straight line in the water running away from the vessel. The rope should not be slack in the water to avoid the chance of cetaceans becoming entangled. Floatation devices along the length of the rope help to reduce line slack, aid swimmers, and prevent swimmers from diving down.

EXCEPTIONS

A surface rope should not be used where conditions may present a risk to human safety, for example, in areas of high wave activity such as in shallow waters close to shore or coral reefs.

A surface rope must not be used for swim-with activities involving humpback whales (*Megaptera novaeangliae*). These are large, powerful animals with large pectoral fins that often display surface active behaviours. Therefore, there is an increased risk of entanglement with the surface rope, leading to potential danger to swimmers, the boat and the whales themselves.

BEST PRACTICE

1 Tourists should be well briefed in advance on the potential impacts of the tour on cetaceans.

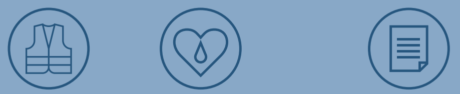

2 Operators must ensure that each vessel has a knowledgeable guide on board.

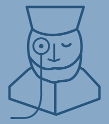

3 Operators must always run a swim test.

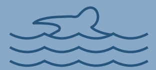

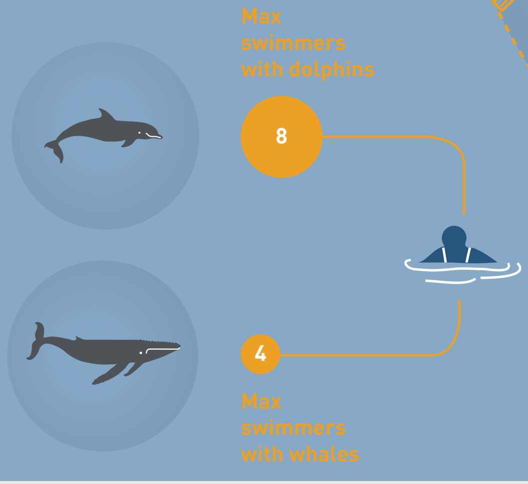

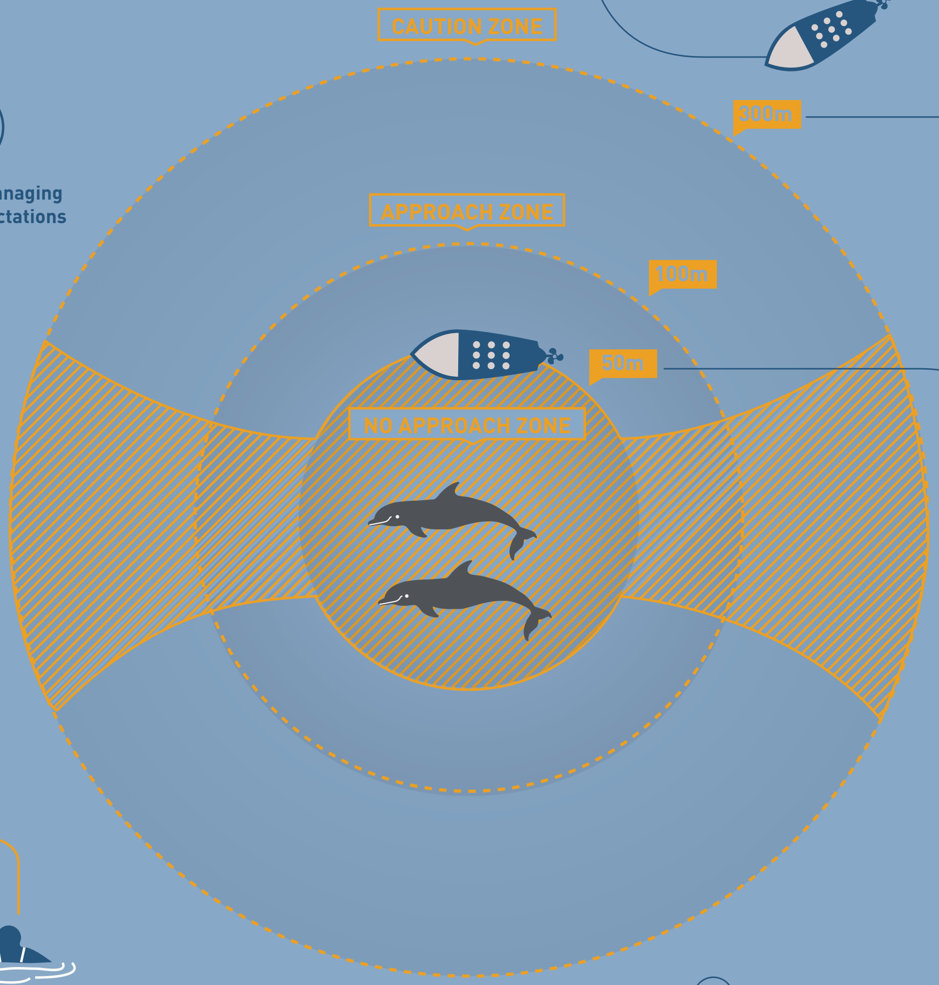

4 The vessel should approach from a direction that is parallel and slightly to the rear of the cetacean.

5 Once within 300m of a cetacean, vessels should:

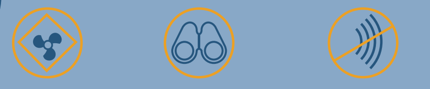

6 If approach zone occupied: stay outside, seek other cetaceans.

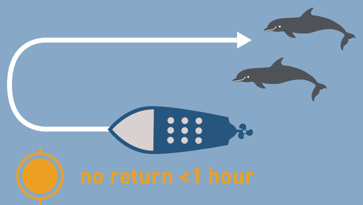

7 Only one vessel is permitted within the approach zone.

8 Tourists should be placed 50m from a cetacean and away from their line of travel.

9 Surface ropes must be used for swimming with wild cetaceans.

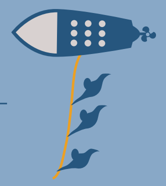

10 The operator should not return to the same cetacean during the same day.

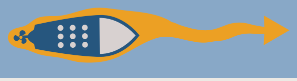

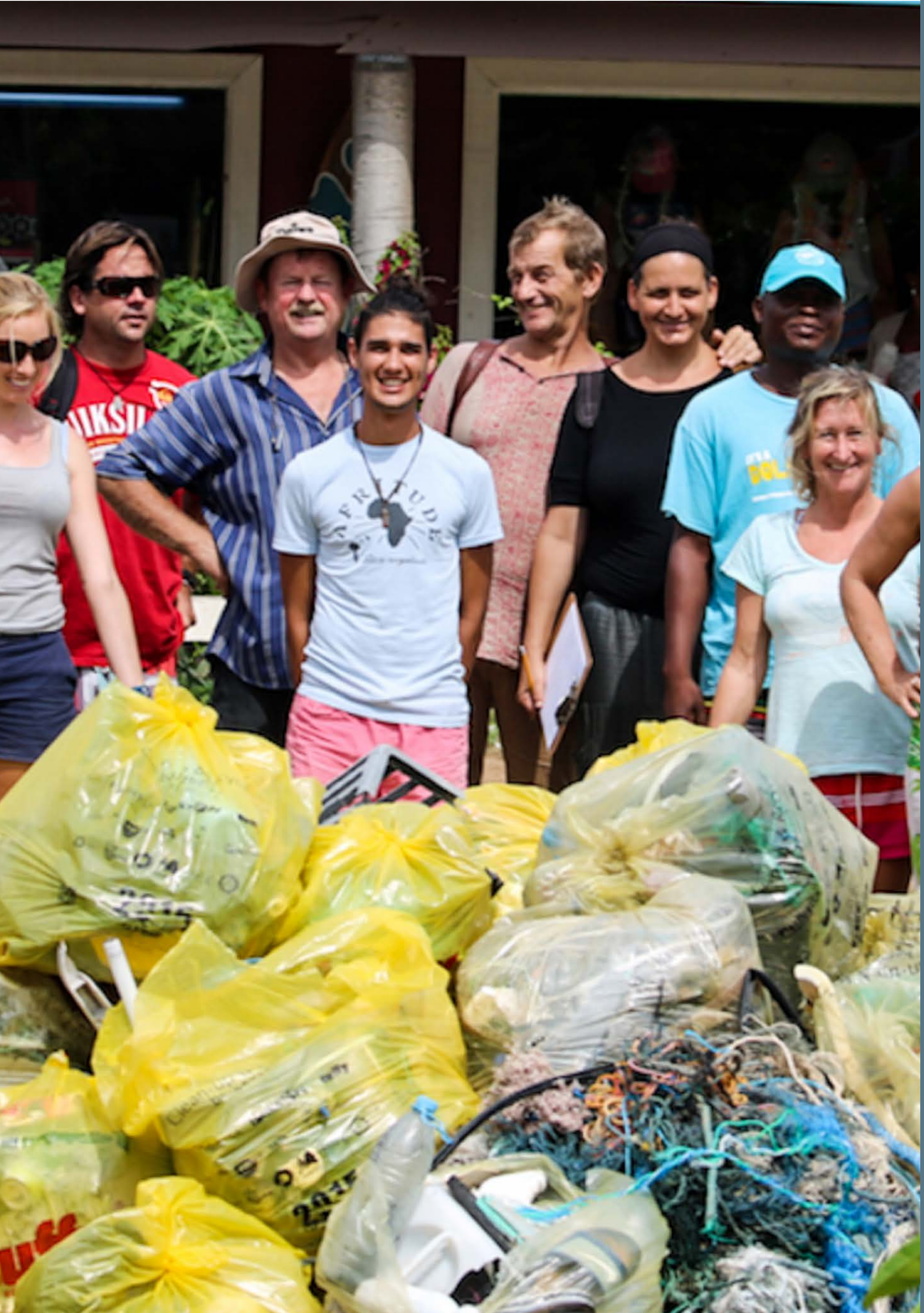

SUSTAINABILITY CRITERIA FOR BEST PRACTICE OPERATORS

CHAPTER 04

© DOLPHIN ENCOUNTOURS

4.1. ENVIRONMENTAL SUSTAINABILITY

4.1.1. Threats relevant to the primary cetacean habitat should be identified and an action plan developed to address those impacts within the control of the operator.

THEME

LOCAL MARINE AND TERRESTRIAL ECOSYSTEMS MUST BE MAINTAINED AND PREFERABLY ENHANCED TO SUPPORT A THRIVING POPULATION OF WILD CETACEANS.

THREE OF THE FOUR SUB-CRITERIA MUST BE MET.

4.1.2. A range of local sustainability initiatives benefitting the marine environment should be undertaken and showcased.

Examples might include, but are not limited to the following:

a) Implementation of measures to encourage reduction in energy use and adoption of renewable technologies;
b) Initiatives and incentives to measure and reduce carbon emissions;
c) Initiatives to improve biodiversity, both marine and terrestrial;
d) Implementation of measures to reduce water pollution;
e) Implementation of measures to reduce plastics, discarded fishing gear and other solid waste entering waterways;
f) Provision of sustainability training for staff;
g) Supporting coral reef or other habitat restoration projects;
h) Participation in patrol programmes or reporting procedures to protect critical habitat;
i) Sale of arts and crafts utilizing local and sustainable materials;
j) Prioritising local produce and local restaurants to provide food for clients;
k) Working with local stakeholders to deliver site-based protection such as through UNESCO Biosphere Reserves, Whale Heritage Sites, Marine Protected Areas, etc.;
l) Award of an additional recognised sustainability accreditation programme, e.g.Blue Flag, Green Tourism Award, ISO 14000, etc.;
m) Use of solar or wind power for operations and boats;
n) Focus on removing ocean plastics during tours and through community events.

4.1.3. Conservation-directed science and research programmes are incorporated into cetacean interactions.

Examples might include, but are not limited to the following:

a) Citizen science initiatives;
b) Collaborative short and long-term data gathering projects run by whale and dolphin watch tour operators working with each other and/or in association with academic institutions or NGOs;
c) Opportunities for researchers aboard whale and dolphin watch boats through internship programmes;
d) Focused research projects that assess the potential impacts of whale and dolphin watching on local cetacean and other wildlife populations in order to improve standards and reduce any direct impacts identified;
e) Onboard tracking systems that ensure that boat movements and time spent with cetaceans can be assessed to ensure responsible practices are being applied.

4.1.4. Conservation-based cetacean research is showcased.

Examples might include, but are not limited to the following:

a) Peer reviewed journal publications;
b) Non-scientific articles, television or radio interviews;
c) Dissemination of the latest research or policy changes to whale and dolphin watching naturalist guides;
d) Display of scientific information prominently in visitor centres, onboard boats, or during school visits;
e) Attendance at scientific conferences and meetings;
f) Research findings published in newsletters, on social media, or in the local press.

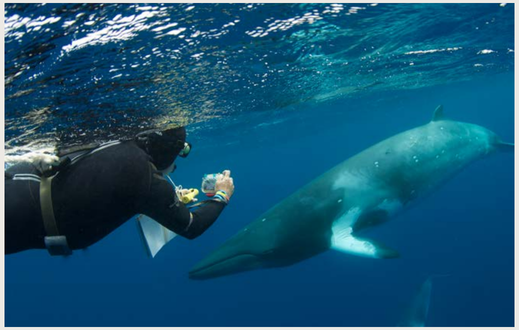

DATA COLLECTION WAS ESSENTIAL IN THE DEVELOPMENT OF DWARF MINKE WHALE SWIM-WITH ACTIVITIES ON THE GREAT BARRIER REEF, AUSTRALIA. THIS RESEARCH HAS LED TO THE CREATION OF A CODE OF CONDUCT THAT IS SENSITIVE TO THE NEEDS OF THE SPECIES AND POPULATION AT THIS LOCATION.

© JOHN RUMNEY/ EYE TO EYE MARINE ENCOUNTERS

4.2. SOCIAL SUSTAINABILITY

4.2.1 Providing information to customers during the tour that reflects cultural, historical, and contemporary practices that harness a greater appreciation for living cetaceans in their natural habitats.

THEME

THE OPERATOR RECOGNISES LOCAL COMMUNITIES AS KEY STAKEHOLDERS AND CELEBRATES THE CLOSE CULTURAL ASSOCIATION BETWEEN CETACEANS AND PEOPLE

THREE OF THE FOUR SUB-CRITERIA MUST BE MET.

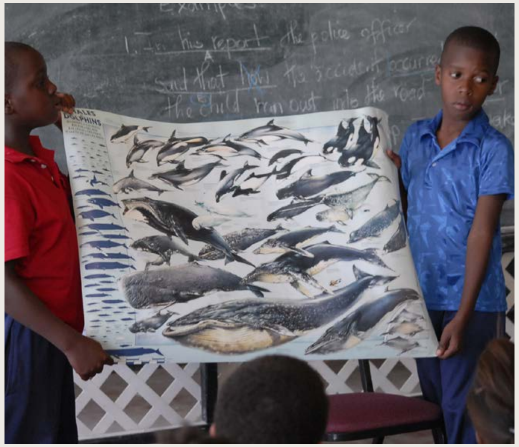

SCHOOL CHILDREN IN DOMINICA PRESENT INFORMATION ABOUT CETACEANS TO THEIR CLASS AS PART OF A MARINE EDUCATION OUTREACH PROGRAMME BY WCA NON-PROFIT PARTNER POTTWALE E.V. © POTTWALE E.V.0

4.2.2. Informing local communities of news, projects and developments, and their rationale, via press, social media or other means.

Examples might include, but are not limited to the following:

a) Delivering regular talks to the public;
b) Attending local events;
c) Utilizing printed and online media, radio and television;
d) Developing a citizen science project as a tool for local community involvement.

4.2.3. Engaging in a minimum of two community events held annually that promote cetacean conservation and encourage a sense of pride, heritage, history, sustainability, and legacy.

Examples might include, but are not limited to the following:

e) School outreach programmes in which children learn about the marine environment and perhaps experience it during a tour;
f) Community beach cleans;
g) Incentivised opportunities for local community groups or schools to meet cetacean experts and take whale and dolphin watching tours;
h) Citizen science projects with a focus on raising awareness among local participants;
i) Opportunities for local students to study cetaceans and their habitats;
j) Awards ceremonies for student projects linked to cetaceans and their habitats;
k) Collaborations with local artists to raise awareness about marine wildlife and conservation;
l) Organisation or participation in celebratory events such as festivals or film nights;
m) Utilise important moments in the calendar year to celebrate marine wildlife, such as the return of migrating whales or turtles to local areas.

4.2.4. Encouraging the importance of cetacean-related works of art in the local community.

Examples might include, but are not limited to the following:

a) Signposting local arts attractions, such as music, dance, and theatre, murals and art galleries, and literature, from both written and spoken traditions;
b) Providing opportunities for customers to purchase local artistic works, including photographs, illustrations, books, crafts, and films.

4.3. ECONOMIC SUSTAINABILITY

THEME

SUSTAINABLE LIVELIHOODS ARE CREATED, GENERATING EMPLOYMENT AND FINANCIAL BENEFITS, TO PROVIDE A CLEAR DEMONSTRATION OF THE ECONOMIC VALUE OF RESPONSIBLE WHALE AND DOLPHIN WATCHING.

ONE SUB-CRITERION MUST BE MET.

4.3.1. Sustainable livelihoods are achieved directly (through boat crewing, tour guiding, research and conservation, etc.) and indirectly (through employment in whale and dolphin watching related services, such as transport, accommodation, local crafts, food suppliers etc.). This should include a balance of jobs (i.e., should ideally not be overly dominated by low paid, informal work).

Examples might include, but are not limited to the following:

a) Collaborate with partner organisations to create opportunities for year-round employment including beyond the whale and dolphin watching season
b) Provide a living wage (a wage that is high enough to maintain a normal standard of living) rather than a minimum wage;
c) Prioritise the use of local products and services that benefit local employers.

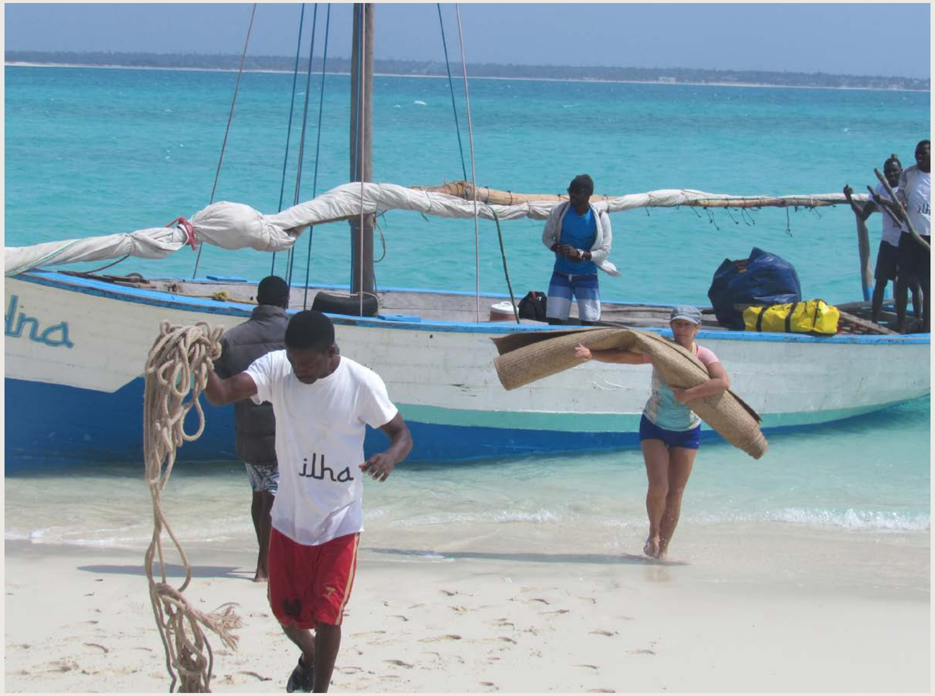

BY PROVIDING EMPLOYMENT AND TRAINING OPPORTUNITIES WHILE USING LOCAL PRODUCTS AND TRADITIONAL SAILING BOATS, WCA RESPONSIBLE WHALE WATCHING OPERATOR ILHA BLUE ADOPTS A SENSITIVE APPROACH THAT BENEFITS THE LOCAL COMMUNITY.
© ILHA BLUE

BEST PRACTICE WHALE AND DOLPHIN WATCHING FOR DESTINATIONS

CHAPTER 05

In order to minimise the impacts of whale and dolphin watching, it is essential that best practices are achieved for the entire site or destination. From a cetacean's perspective, there is little value in the existence of one responsible operator running tours with a minimal impact, if all of the other tours encountering the same cetacean are disruptive.

Stakeholders in responsible whale and dolphin watching destinations work collaboratively to ensure that whale and dolphin watching is managed sustainably. Tour operators, conservationists, NGOs, academic institutions, and government agencies develop strategies to communicate with each other, share information, and monitor current practices. Best practice whale and dolphin watching should always act based on research that identifies the social, economic, and environmental carrying capacity of the activity. Incorporating that knowledge into an effectively implemented management plan that involves all relevant stakeholders is the responsibility of any government or managing organisation wishing to maintain whale and dolphin watching activities as part of its tourism portfolio.

Responsible management practices are the foundation for the Whale Heritage Site initiative; the World Cetacean Alliance's flagship programme to accredit locations that achieve best practice for responsible whale and dolphin watching. Whale Heritage Sites are outstanding locations where cetaceans are embraced through the cultural, economic, social, and political lives of associated communities, and where people and cetaceans coexist in an authentic and respectful way. Their aim is to preserve cetacean species and their habitats on a global scale by harnessing sustainable tourism practices and encouraging local communities to embrace marine conservation.

The following management advice for Whale Heritage Sites is designed to assist individuals and organisations wishing to develop responsible whale and dolphin watching practices for tourism sites.

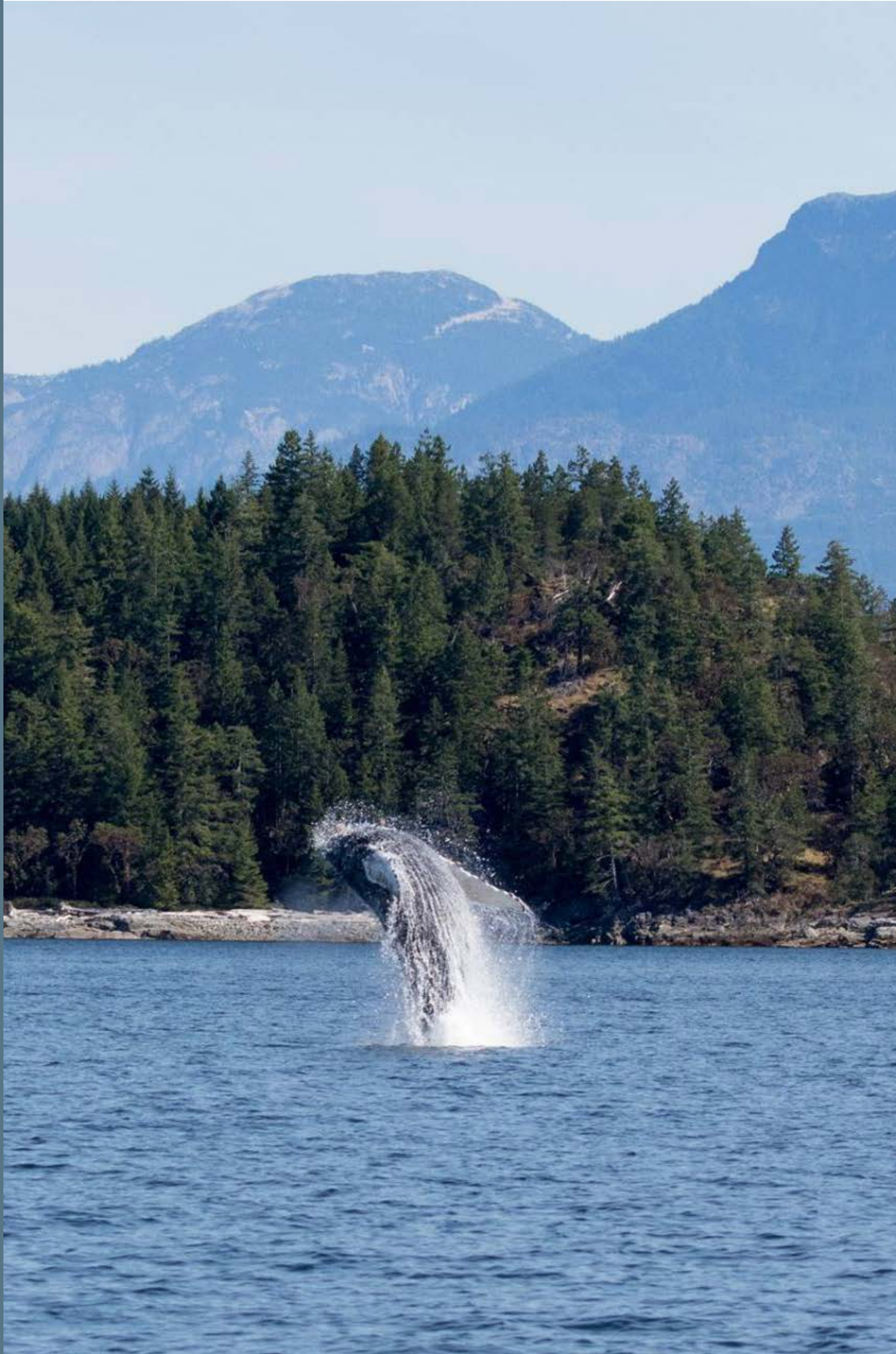

© EAGLE EYE ADVENTURES

CASE STUDY 03

VANCOUVER ISLAND NORTH

Whale and dolphin watching by boat in Vancouver Island North first began in 1980 with 40 guests coming aboard Stubbs Island Whale Watching that year, pre-dating all other killer whale-focused marine tourism in the world. The whale and dolphin watching industry developed as a result of the attention brought to the research on killer whales in the Vancouver Island North area. Pioneer whale and dolphin watching boats followed the lead of researchers and used, for whale watching purposes, the same distances that researchers had found did not interfere with the whales' behaviours.

Michael Bigg published the first guidelines for approaching whales in 1986, and standards of practice and the culture of the whale and dolphin watching industry developed organically, over time, and cooperatively. Today, forty companies from several ports employ over 250 staff and use several types of vessels including sailboats, powerboats, and kayaks to view whales off Vancouver Island North (7).

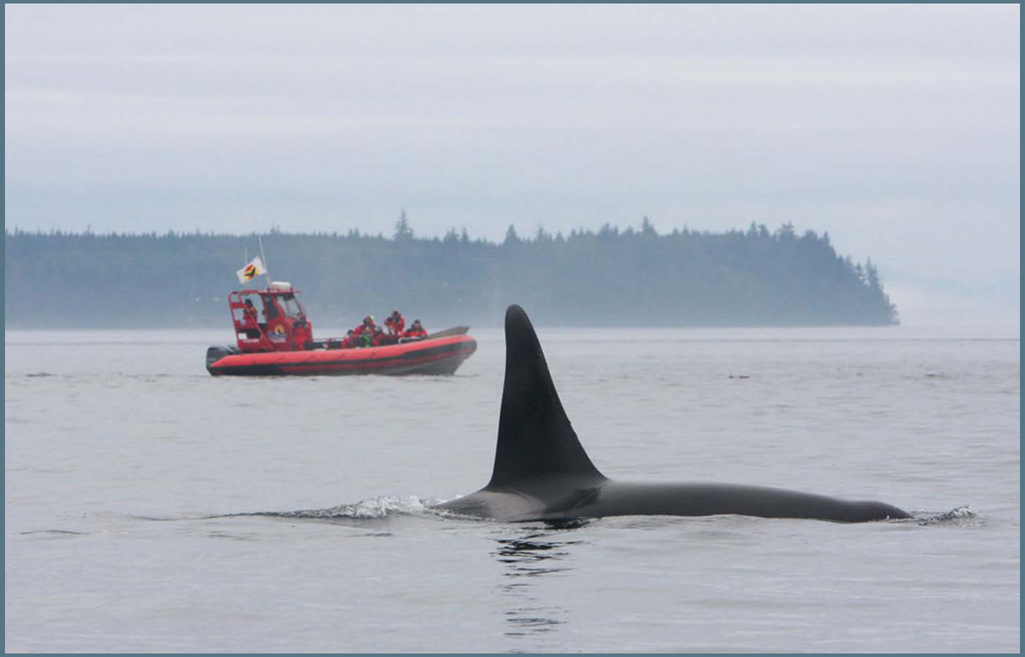

RESPECTING LOCAL GUIDELINES, A WHALE WATCHING BOAT IN VANCOUVER ISLAND NORTH VIEWS A KILLER WHALE AT A DISTANCE OF 100 METRES. © JARED TOWERS

REGULATORY FRAMEWORK

Under the Canadian Marine Mammal Regulations, it is prohibited to disturb a marine mammal unless you are hunting it, which requires a licence. In 2002, Department of Fisheries and Oceans (DFO) began a consultation process to amend the current Marine Mammal Regulations in line with requirements for whale and dolphin watching. After two phases of consultation, the proposed amendments to the regulations await final publication, but are likely to: define 'disturbing' a marine mammal; provide for a general minimum approach distance of 100m for marine mammals with some exceptions; provide for licensing of activities, such as research, that may require disturbing marine mammals; and require reporting whenever there is accidental contact, i.e. entanglement or a collision with a marine mammal. Licences for whale and dolphin watching operations are currently considered unnecessary.

BE WHALE WISE GUIDELINES

The 'Be Whale Wise' cetacean viewing guidelines provide clear guidance on proper behaviour when whale and dolphin watching. DFO includes the Be Whale Wise Guidelines on their website and they are also promoted in the United States, creating cross-border standards of practice. The Be Whale Wise Guidelines provide direction so that boat operators do not violate the "do not disturb" regulation. On a practical level, operators and boaters treat the guidelines as regulations and follow them, even though they are voluntary. This may be due to the fact that the Be Whale Wise Guidelines were developed collaboratively over time, and there was a mechanism for habitat and stewardship protection which provided funding to two programmes in the Pacific Northwest, Straitwatch and Soundwatch, to educate boaters and collect data. These programmes put a spotlight on anyone not complying with the guidelines, encouraged everyone to understand the guidelines, and laid the groundwork to ensure that these are treated as standard practice in the Vancouver Island North and Pacific Northwest area.

NORTH ISLAND MARINE MAMMAL STEWARDSHIP ASSOCIATION (NIMMSA)

NIMMSA promotes sustainability of the whale and dolphin watching industry, and actively engages in initiating or funding conservation-based stewardship activities that benefit marine mammals and their environment off Vancouver Island North. For example, NIMMSA members adhere to an operational Code of Conduct tailored to the Vancouver Island North context that benefits cetaceans by ensuring that they are approached in a consistent and respectful manner within the confines of the law. Adherence to this Code is mandatory for NIMMSA members. NIMMSA members have been shown to consistently adhere to this Code of Conduct during tourism operations. Several NIMMSA members also fly a whale flag when their vessel is engaged in whale and dolphin watching to alert other boaters that there are whales nearby and that they should be vigilant so as to avoid being in contravention of whale and dolphin watching guidelines or regulations.

5.1. COLLABORATIVE DEVELOPMENT OF LOCAL GUIDELINES

The participation of whale and dolphin watching tour operators in both the development and implementation of guidelines or regulations is critical to their success. Tour operators should be actively involved in the process, for example through a whale watching association, an annual review, or other meetings, training sessions, workshops etc.

The following list provides some options for developing guidelines or regulations collaboratively, reviewing them regularly, and clearly communicating them to relevant audiences:

- Organisation of regular communication between all stakeholders;
- Collaboration on guidelines or regulations should be cyclical;
- Regular skipper training or sharing of best practice between skippers with variable experience;
- Regular scientific input and output, with scientific knowledge contributing to the evolution of guidelines or regulations;
- Involve recreational boaters in educational programmes;
- Anonymous reporting of bad practice with a feedback mechanism to discuss at meetings;
- Providing incentives for compliance;
- Encouraging input from citizen science projects run collaboratively between whale and dolphin watching tour operators;
- Encourage partnerships with local NGOs or academic institutions able to provide independent advice and expertise.

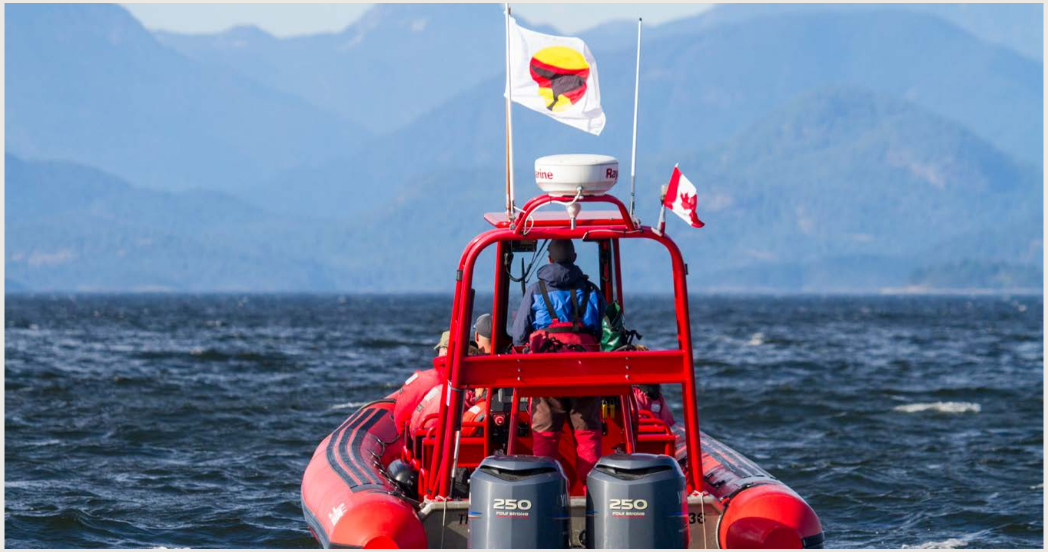

WHALE WATCHING BOAT FLIES THE WHALE WISE FLAG AS IT HEADS OUT FOR A TRIP IN VANCOUVER ISLAND NORTH.
© STEPHANIE STEFANSKI

5.2. MONITORING AND ENFORCEMENT

The authority responsible for monitoring and enforcing guidelines or regulations for whale and dolphin watching varies by location. In many places, the relevant local government authority performs this task through, for example, the Department for the Environment, National Park or Marine Park Authority, the Navy, or Coastguard. The role of government in monitoring is important in many areas because it is also the authority able to enforce penalties for infringements of the guidelines or regulations. Some government authorities have also found that incentive schemes rewarding best practice act as an effective method for improving standards among whale and dolphin watching tour operators.

However, other stakeholders can also play a very important role. For example, in some places academic institutions and NGOs have strategies for reporting bad practice and a mechanism for feeding this information back to government, whale and dolphin watching companies or both, to encourage change. In other cases, tourists have been encouraged to report bad practice, and NGOs have run independent observer schemes (sometimes using the 'secret shopper' technique) to monitor whale and dolphin watching tours.

Perhaps one of the most important, cost-effective, and overlooked ways to ensure guidelines or regulations are adhered to is through self-regulation. This requires whale and dolphin watching tour operators to work collaboratively to ensure that the rules are followed at all times. This is only possible if there is good communication between operators, mutual respect, and an acknowledgement that all operators depend upon a shared resource. Self-management is most effective in locations where whale and dolphin watching tour operators have formed a whale watch association, or have regular meetings to discuss any issues arising. These meetings should ideally involve other relevant stakeholders, including academic institutions, government representatives, NGOs, and the public.

5.3. TRAINING

Regular training should take place for skippers, crew, guides, onshore staff, and other stakeholders to ensure ownership and maintenance of responsible standards for interactions. Training can be through an official training course involving classroom activities and practical training on the water. Alternatively, the sharing of best practice concepts between skippers, crew, guides, onshore staff, and other stakeholders with local expertise is also important. Training courses are offered by NGOs, academic institutions, government, industry, etc.

5.4. LICENSING

For many places, ensuring that commercial whale and dolphin watching can only be undertaken by a limited number of permitted (licensed) operators is the essential first step to sustainable management. Ideally, the number of permits available should be based on an ongoing scientific assessment of the carrying capacity for that location. Decisions on the carrying capacity of the site should be evidence-based as a result of dedicated and ongoing scientific research on the local cetacean populations, as well as engagement with stakeholder groups, including tour operators, NGOs, scientists, and the public.

There are different methods for limiting the number of permits available. In South Africa, permits are generally limited to one permit per location. In Kaikoura, New Zealand, permits are limited by tourist activity, so the tour operator running dolphin swim programmes cannot also run whale watching trips. In the past, Hervey Bay, Australia, implemented a zoning system, separating boats by restricting permit holders to specific zones. One of the most advanced systems for permit use for whale and dolphin watching tour operators is in South Africa. The system is founded on the principles of 'continuous criteria' and 'permit re-application', whereby operators are required to provide evidence of their high standard of responsible whale and dolphin watching in order to successfully retain a permit on reapplication every five years. Examples of the evidence provided include cooperation with researchers, equality within the workforce, and involvement in educational programmes.

Frequent monitoring of licensed operators and enforcement of permit agreements is an effective mechanism to ensure responsible practices. The costs for enforcement may be recouped either through the licence fee or through a tax paid per passenger.

5.5. TIME-AREA CLOSURES AND ZONING

Allowing periods of time or areas where cetaceans cannot be disturbed can be achieved by applying one or more of the following management options, all of which require in depth knowledge of the ecological requirements of local cetacean species and populations.

1. Cetacean tourism activities are prohibited at allotted times, reducing pressure during times of biologically important activities such as foraging or resting;
2. Exclusion zones are established, restricting tourism access to areas where cetaceans may be particularly vulnerable to disturbance such as nursing grounds or resting areas;
3. Zoning, in which each licensed operator is allocated a separate area.

5.6. MULTI-DAY EXCURSIONS

Multi-day excursions are a particularly effective mechanism for reducing the impact of swim-with tours. Short trips that only last a few hours, and especially those that offer a guaranteed swim-with experience, can put pressure on the guide and skipper, resulting in swim-with attempts that may compromise the welfare of cetaceans or swimmer safety. A shift away from short trips of a few hours to multi-day tours can help to relieve this pressure.

Multi-day trips tend to offer clients a more holistic and educational experience, and include full training and careful preparation before the first swim-with attempt. The practice of swimming with cetaceans in the wild is more likely to be only one of several motives for the trip, providing the operator with the flexibility to place swimmers in the water only when the conditions really are appropriate to do so.

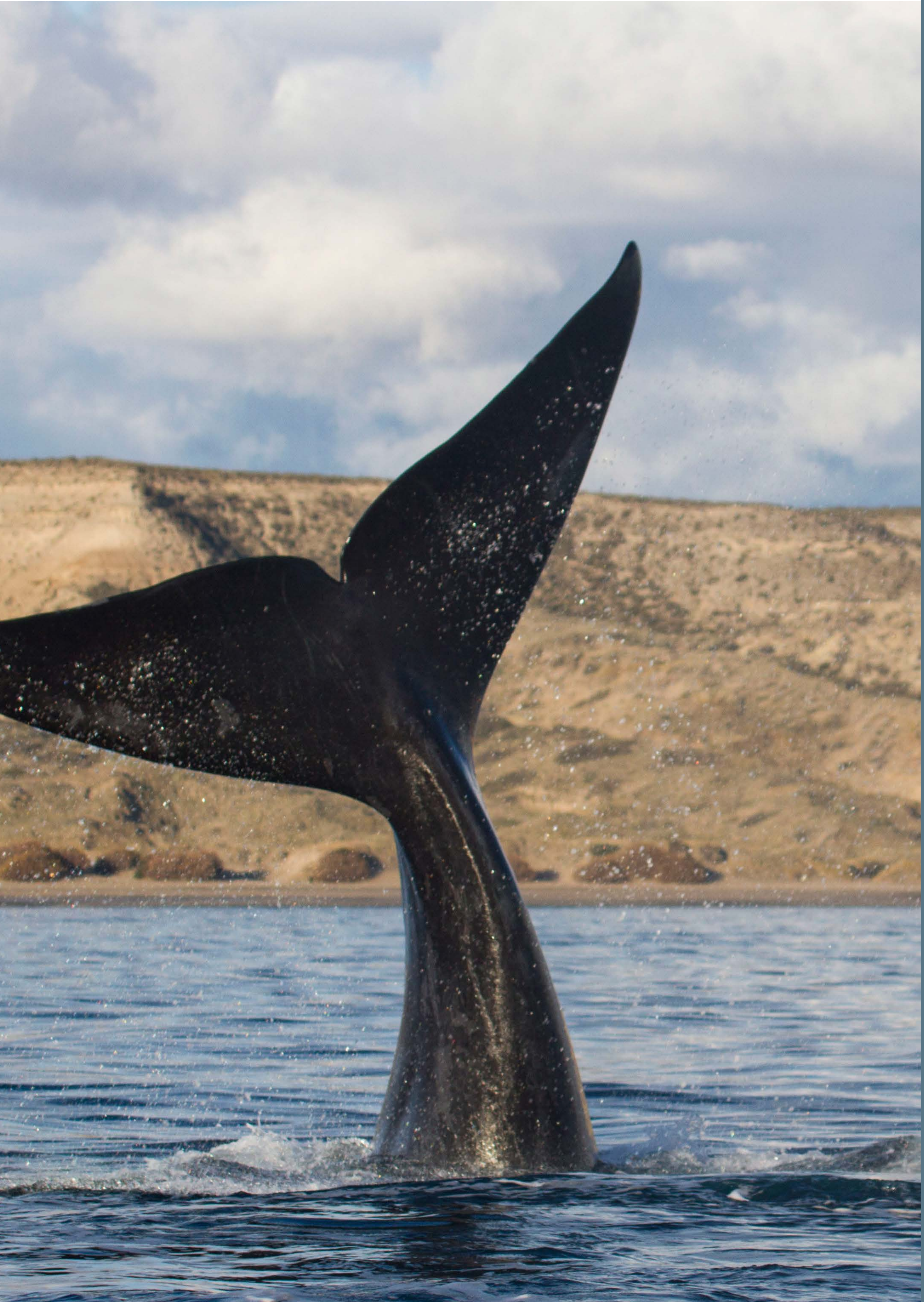

THE SCIENCE BEHIND WHALE AND DOLPHIN WATCHING: IMPACTS AND BENEFITS

CHAPTER 06

Our history with cetaceans reaches far beyond our recent expansion into tourism, with the lives of humans and cetaceans intertwining for millennia. The links between whales, dolphins and people have been embedded in many cultures since prehistoric times, including depictions of cetaceans in cave paintings from the Neolithic period. Over the years, the connection between humans and cetaceans has been solidified through hunting, mythology, cultural celebrations, the arts, media, and tourism.

© STEPHANIE STEFANSKI

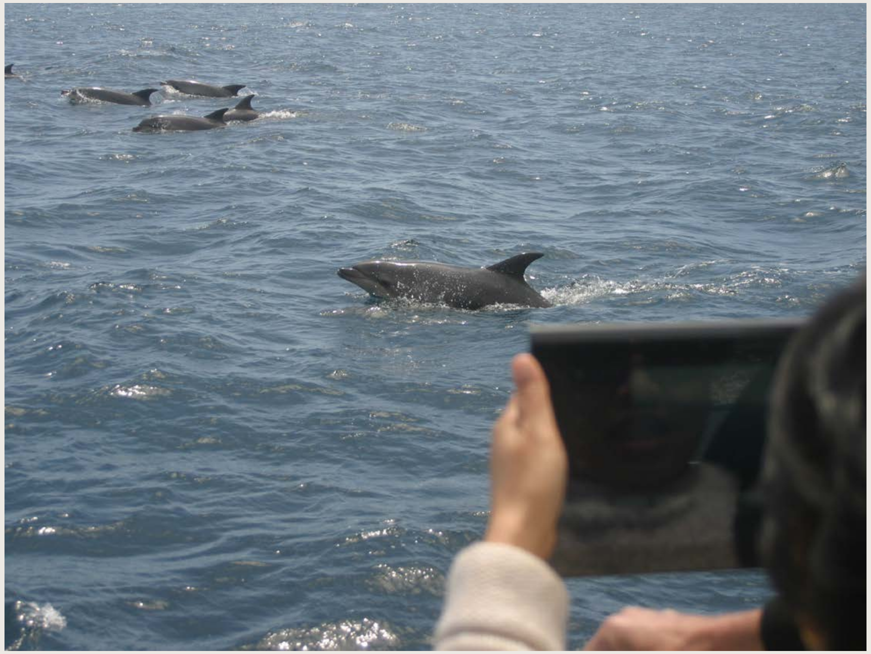

TOURISTS HELP TO GATHER PHOTO IDENTIFICATION DATA FOR CITIZEN SCIENCE RESEARCH WITH WCA RESPONSIBLE WHALE WATCHING OPERATOR AZORES WHALE WATCHING FUTURISMO. © DYLAN WALKER

6.1. BENEFITS

In recent years, the consumptive use of cetaceans through whaling has rapidly declined and commercial whale and dolphin watching has increasingly proven to be a viable and beneficial economic alternative. In many regions across the world the value of a live cetacean is now recognised as far exceeding its previous value when dead. One example of this is in the village of Kizimakzi in Zanzibar. Since the first dolphin tour operation began in this village in 1992, the whale and dolphin watching industry has replaced the traditional hunting of Indo-Pacific bottlenose dolphins (*Tursiops aduncus*), with the last hunt occurring in 1996. The economic value of the dolphin tourism industry in this region is evident, providing employment and income for villagers [8]. Similarly, Taiwan has also transitioned from whaling to tourism as a source of income [9]. In some regions, such as the Kingdom of Tonga, the tourist demand for commercial whale and dolphin watching and swim-with trips remains a strong counter-argument to the resumption of commercial whaling [10,11].

Whale and dolphin watching has the potential to be a powerful tool to benefit marine conservation. Studies have shown that cetacean watching experiences, when combined with high quality environmental education and interpretation, significantly increase tourists' knowledge of conservation issues and their empathy towards these animals [12].

Whale and dolphin watching can also directly contribute to the conservation of cetaceans and the marine environment by providing a platform for scientific research. Many whale and dolphin watching operators collect their own data, most commonly in the form of photographic identification images, providing opportunities for researchers on board to conduct studies. With millions of tourists participating in this activity each year, the potential for citizen science, in which the public collect and submit important data to scientists, is vast, as demonstrated by new and innovative projects such as Happywhale (www.happywhale.com). This has led to the publication of many research papers on the social, economic, and environmental impacts of whale and dolphin watching.

6.2. IMPACTS: BOAT-BASED WHALE AND DOLPHIN WATCHING

There is substantial evidence that whale watching can have a detrimental effect on cetacean populations. Poor boat handling, too many operators, and the cumulative effects from tourism and other environmental pressures, likely play a large part in this trend. When there is evidence that negative effects have both short and long-term impacts, then the sustainability of the industry should be challenged, and appropriate management practices implemented immediately.

Physical injury or even death as a result of strikes from tourism boats are the most visually obvious and dramatic sign of harm to cetaceans. This is of concern as the number of tourism boats approaching these animals in unregulated locations continues to increase, often associated with irresponsible boat handling; including travelling at high speeds, rapid changes in direction, and chasing cetaceans. Furthermore, there is evidence that whales and dolphins can become habituated to boats that are repeatedly in the area, forgoing avoidance manoeuvres and even approaching boats [13]. This habituation could potentially lead to an increased risk of strikes from these boats.

A growing catalogue of research has focused on the short-term alteration of the animals' normal behaviours as a result of exposure to tour boats and

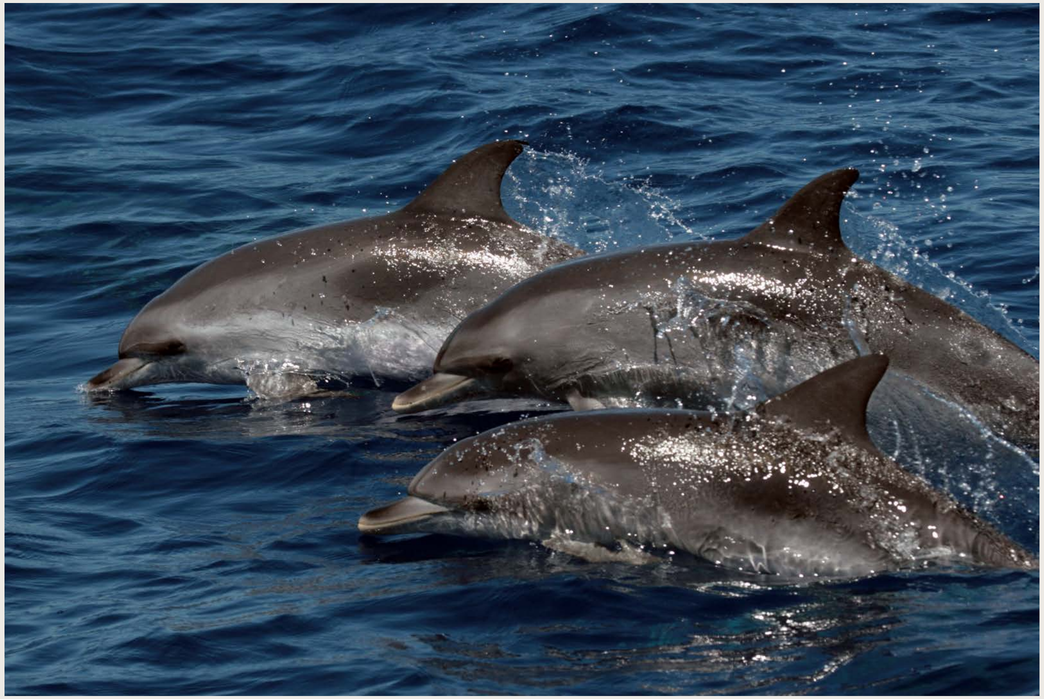

THREE SPOTTED DOLPHINS PASS WCA RESPONSIBLE WHALE WATCHING OPERATOR WHALE WATCH AZORES.
© WHALE WATCH AZORES

swimmers. These changes can manifest in a variety of ways such as alterations in acoustic behaviour, group cohesion, horizontal and vertical dive patterns, and the timing of diving and respiration rates [14]. For example, in Doubtful Sound, New Zealand, the acoustic behaviour of common bottlenose dolphins (*Tursiops truncatus*) was affected by the presence of tour boats. This resulted in reduced group cohesion and coordination, particularly in groups with calves [15]. This effect was also detected during swim-with tours to see Burrunan (bottlenose) dolphins (*Tursiops australis*) in Port Philip Bay, Australia [16]. In Doubtful Sound, New Zealand, common bottlenose dolphins displayed increased surface activity, deep dives and erratic movements during interactions with tour and recreational boats, particularly when the conditions of the New Zealand Marine Mammal Protection Regulations (MMPR) were violated [17]. Similarly, humpback whales (*Megaptera novaeangliae*) altered their horizontal movement patterns, either to avoid or approach boats, and dived for longer periods in the presence of whale watching boats off New South Wales, Australia [18].

Tourism presence can also disrupt biologically important activities such as foraging, resting, nursing and socialising. In the Bay of Plenty in New Zealand, interactions with tour boats significantly reduced the time short-beaked common dolphins (*Delphinus delphis*) spent foraging, and once disturbed, these dolphins took twice as long to return to this activity compared to control conditions [19]. Similar alterations in biologically important behaviours have been observed across a variety of species and locations. For example, a reduction in resting, foraging and socialising as a result of tourism boat presence was observed in Indo-Pacific bottlenose dolphins in Zanzibar [20]. Common bottlenose dolphins in the Bay of Islands, New Zealand, also rested less as boat numbers increased [21], and southern right whales (*Eubalaena australis*) in Península Valdés, Argentina, decreased their resting and socialising behaviours and increased travelling when subjected to simulated swim-with tourism [6].

Due to a lack of long-term studies, the true consequences of these short-term effects remain largely unknown. However, behavioural changes, particularly away from foraging and resting states, can potentially alter the energy budget of the animals, resulting in reduced travelling efficiency, fecundity and ability to avoid predation. A study in 2005 was the first to record the negative effects of long term disturbance from tourism boats on the reproductive rates of Indo-Pacific bottlenose dolphins in Shark Bay, Australia; showing that long-term disturbance reduced reproductive success [4]. Although the precise mechanisms that lead to these trends is not fully understood, several suggestions have been made. Reduced periods of resting and socialising could result in a decline in socialising behaviour and therefore lower pregnancy rates [22], as well as poorer calf fitness due to reduced opportunities for nursing during resting periods [5]. Similarly, tourism pressure during pregnancy has been suggested as a cause of reduced calf survival of common bottlenose dolphins in New Zealand [23,24] and elsewhere [4,25].

These behavioural changes are often witnessed and reported by the operators themselves. When questioned for Global Best Practice Guidance for Responsible Whale and Dolphin Watching, many stakeholders involved in whale and dolphin watching noted the occurrence of behavioural changes at least sometimes during tourism activities. Many of these behavioural changes were not viewed as negative (e.g. avoidance or stress related) but confirm that cetaceans regularly alter their behaviours in proximity to boats or swimmers. Cetaceans are sociable, curious animals, and operators and tourists often report individuals seeking out boats or humans. It is important to understand, however, that even seemingly positive encounters with cetaceans could potentially have long-term negative effects if these activities take time away from behaviours that are vital to their health and survival [26,27]. A long-term study of Burrunan (bottlenose) dolphins in Port Philip Bay, Australia, found that over the two time-periods observed, neutral responses to tourism activities (no change in behaviour) decreased over time. This means that dolphins increasingly altered their behavioural states (whether to avoid or approach) as a result of long-term tourism activity [26].

In some instances, cumulative pressure from whale and dolphin watching can even lead to populations being displaced from favoured locations. When the costs of disturbance outweigh the benefits of staying in a preferred habitat, an individual, group or even population may abandon a site in favour of a less disturbed one. This may involve compromising by moving to an area with, for example, reduced food availability. In Shark Bay, Australia, an increase from one to two dolphin watching boats led to the reduction in abundance of the local group of Indo-Pacific bottlenose dolphins equivalent to one in seven individuals. This decline was attributed, at least in part, to the displacement of more sensitive individuals away from the disturbed site to a nearby undisturbed site [25]. In the Kisite-Mpungutu Marine Protected Area in Southern Kenya, 78% of a small population of Indo-Pacific bottlenose dolphins temporarily left the study area when the peak number of boats entered the MPA. This percentage greatly reduced with the introduction of a code of conduct [28]. It is important to note, however, that if an animal does not move from

a disturbed site, it does not mean that it is not affected by disturbance. Such decisions could be influenced by other factors such as suitability and proximity of other sites, density of competitors at other sites, etc. Those animals that do not move away from disturbance could, therefore, be the most vulnerable to population level effects [29]. Furthermore, disturbances impacting particularly sensitive individuals may cause them to move away from the disturbed location to areas where they are more vulnerable [30].

Tourism impacts on the behaviour of cetaceans could potentially also have a knock-on effect on ecosystem dynamics. For example, the feeding techniques employed by short-beaked common dolphins in the Hauraki Gulf, New Zealand, have been found to provide opportunities for other species, including the Australasian gannet (*Morus serrator*) and Bryde's whale (*Balaenoptera brydei*) [31]. The disturbance of feeding short-beaked common dolphins could therefore have a detrimental effect on other species feeding in association with them [32].

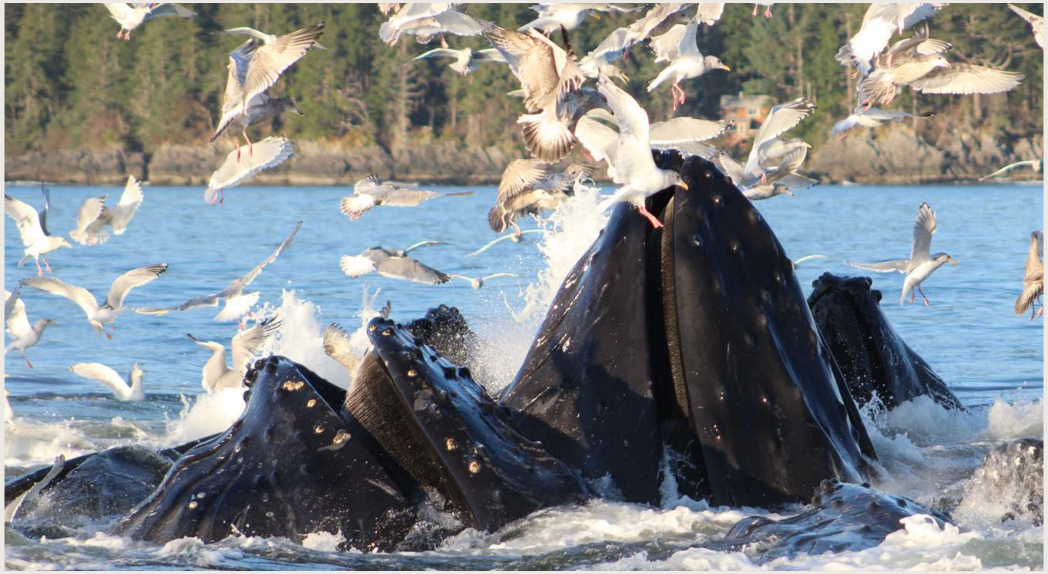

HUMPBACK WHALES LUNGE FEEDING ALONGSIDE SEABIRDS OFF THE COAST OF ALASKA, USA.
© NEIL MCDERMOTT/ A WHALE'S SONG EXPEDITIONS

6.3. IMPACTS: SWIMMING WITH WILD CETACEANS

The specific impacts of swim-with activities on wild populations of whales, dolphins and porpoises are often difficult to disentangle from the overall impact of boat-based whale watching. However, any impacts have the potential to be exacerbated during a swim-with trip if not properly managed. For example, long term exposure of common bottlenose dolphins to swim-with activities in the Bay of Islands, New Zealand, showed a decrease in swim-with attempt success and an increase in avoidance behaviour over two time periods from 1994-1998, suggesting these animals were becoming sensitised to this activity through continual exposure [33]. This type of decreased success rate because of tourism pressure illustrates how long term negative effects are not only detrimental to the animals but can also reduce the quality of tours.

The potential for injury to humans during swim-with experiences is another important consideration. Cetaceans are large, powerful animals and therefore have the ability to injure humans whether accidentally or intentionally. Although the potential of injury to humans swimming with cetaceans is extremely low, incidents have been recorded. Examples include a woman dragged down to approximately 15m below the surface by a short-finned pilot whale (*Globicephala macrorhynchus*) in the waters of Hawaii in 1992 [34] and a breaching humpback whale in Vava'u, Tonga, almost landing on swimmers in 2016 [35]. These accounts demonstrate why swim-with activities should be practiced by adopting precautionary principles to limit any risk to human safety. Additionally, cetaceans carry parasites and microorganisms that have the potential to be transferred to humans if close contact occurs, and vice versa [36], posing a risk to both cetaceans and humans.

Innovative and precautionary management approaches have been running in several regions across the world in the form of adopted guidelines or codes of conduct set by governing bodies or voluntarily by operators that strive to allow cetaceans to have control over all encounters (e.g. CASE STUDY 1).

However, there is a lack of scientific evidence examining the effects of guidelines specifically relating to swim-with activities in the wild, including the number of swimmers allowed during an encounter and time spent in the water. The lack of scientific research on the effectiveness of specific guidelines illustrates the importance of involving the operators themselves in future research efforts, and emphasises the need to take a precautionary approach at all times.

6.4. IMPORTANCE OF GUIDELINES

It is important to monitor and manage whale and dolphin watching, not only to protect cetaceans, but also to ensure a sustainable industry that provides long-term employment opportunities and benefits local communities (26). When thoughtfully developed, the implementation of operating guidelines for whale and dolphin watching activities are a vital mechanism to mitigate the negative impacts of cetacean watching in order to ensure its economic and environmental sustainability. In many regions, guidelines have been developed and implemented in the form of government regulations or voluntary codes of conduct, with varying degrees of success.

The benefits of following scientifically informed guidelines is evident in the scientific literature. For example, Burrunan (bottlenose) dolphins in Port Phillip Bay, Australia, were found to be significantly influenced by the type of approach tour boats used. Higher levels of avoidance were exhibited when illegal (direct or overtaking) approaches were made by boats compared to legal (parallel) approaches [26]. Similar responses have been recorded in several species in New Zealand; for example, Hector's dolphins (*Cephalorhynchus hectori hectori)* in Akaroa Harbour [27], and common bottlenose dolphins in the Bay of Islands [33] and in Doubtful Sound [17]. Humpback whales off the coast of southeast Australia avoided boats more frequently when boats were not in compliance with regulations and travelling within the 100m approach limit, compared with those travelling outside this limit [18]. This research demonstrates that compliance with scientifically reinforced regulations is critical to the sustainability of the industry and essential to minimise impacts on cetacean populations.

The impact of tourism on cetaceans is likely to differ across locations and species, making management particularly challenging [37]. The need for site, species and even population specific guidelines to help reduce the negative impact of tourism is undeniable. Many species have unique attributes, and ideally require focused management tailored to their specific needs. Many Spinner dolphin (*Stenella longirostris*) populations, for example, have a unique foraging pattern; feeding at night in open waters and returning to sheltered, protected bays to rest during the day. These times of rest are particularly important and leave them vulnerable to disturbance. Tourism activities targeting this species therefore require specific guidance to protect their resting sites.

Small localised populations, such as the Burrunan (bottlenose) dolphins in Port Phillip Bay, Australia are also particularly vulnerable due to their restricted range, making them priorities for specific and careful management. Conversely, the wide-ranging movements of humpback whales in the Kingdom of Tonga have important implications for the management of whale watching and swim-with activities in this region, with management plans needing to cover a wide geographical area to ensure their protection [10].

Although operators may feel that adhering to precautionary guidelines will reduce customer satisfaction, research has shown that the opposite is true. Large numbers of boats around dolphins in Port Phillip Bay, Australia, resulted in reduced levels of customer satisfaction due to perceived crowding [38]. Furthermore, customer satisfaction has been shown to increase when the importance of guidelines are clearly explained [39]. This evidence suggests that tourists have the potential to play an important role in encouraging compliance.

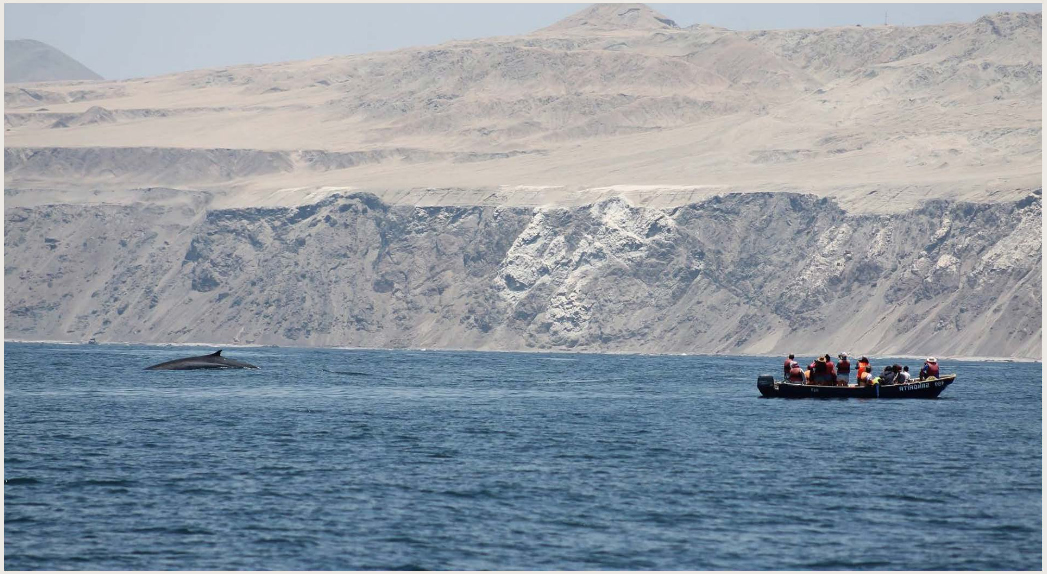

A WHALE WATCHING VESSEL OBSERVES A FIN WHALE AT A SAFE DISTANCE OF 100M OFF THE COAST OF CHILE.
© CIFAMAC

CETACEANS IN CAPTIVITY

CHAPTER 07

As a result of mounting evidence that the welfare needs of cetaceans are compromised in captivity, recent events have seen considerable changes in public opinion about their welfare in aquariums (dolphinariums). This has included breeding bans, the introduction of legislation to phase out dolphinariums in some countries, and pressure from tour operators to improve standards within existing facilities. With over 3,000 long-living cetaceans currently in captivity worldwide [2], there are an increasing number of experts and initiatives looking at what the future might look like for these individuals as these changes progress.

Many of those cetaceans that have spent numerous years living in captive facilities, or were born into them, may be considered unsuitable for release into the wild. As a proposed resolution to this, there is growing support for the development of seaside sanctuaries to provide places to retire captive cetaceans and, where appropriate, rehabilitate them for later release into the wild. These sanctuaries will likely be located in fenced off coves or bays, providing a safe space and enriched environment in which their health and welfare can be monitored and sustained. These areas may be accessible to both the staff that will provide the care for these animals, and also to the public, whose controlled access may provide fundamental financial support to keep the sanctuaries operational. As yet, there are no operating seaside sanctuaries, but several are under development and are likely to be completed soon.

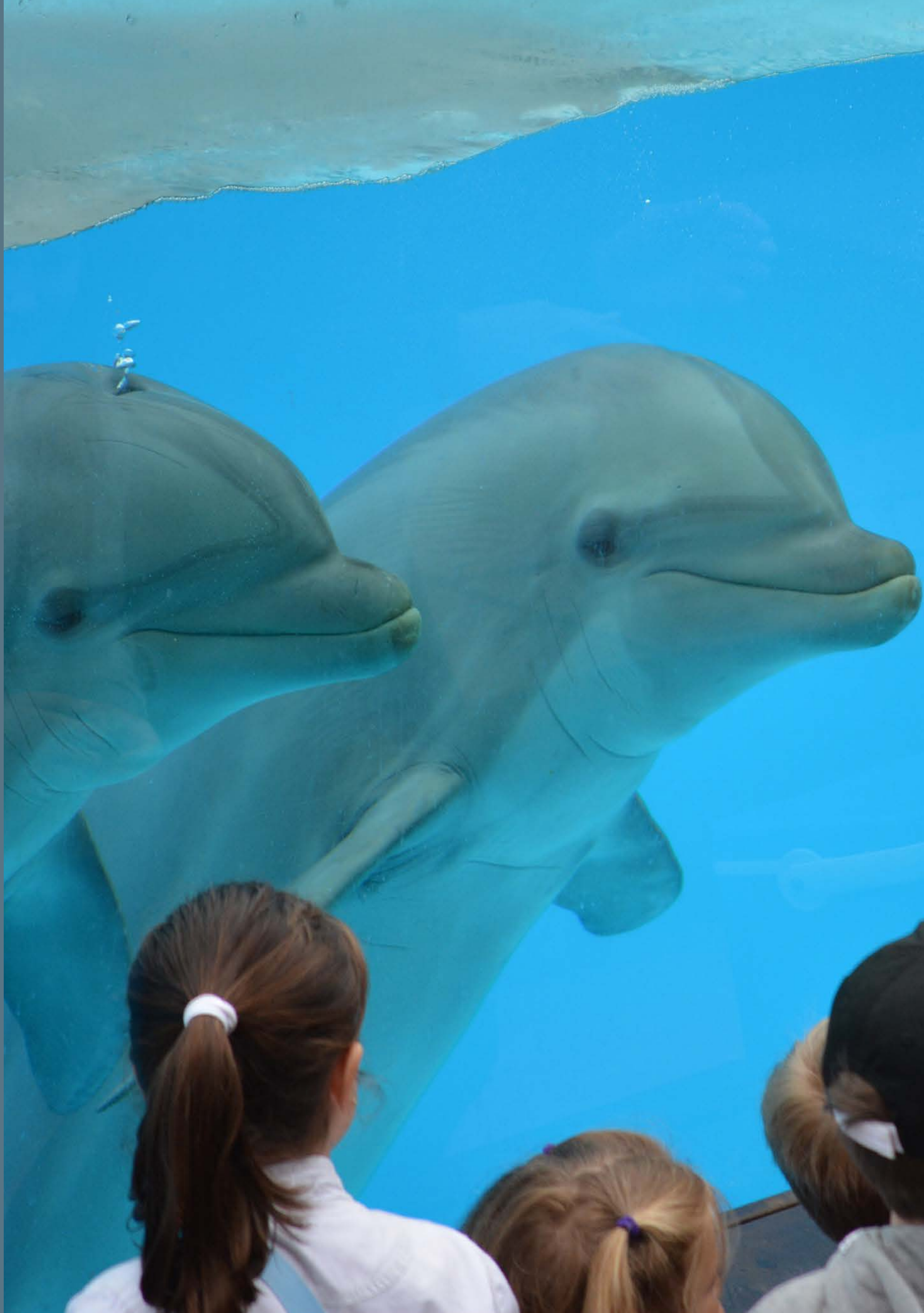

© ALEXANDER SÁNCHEZ

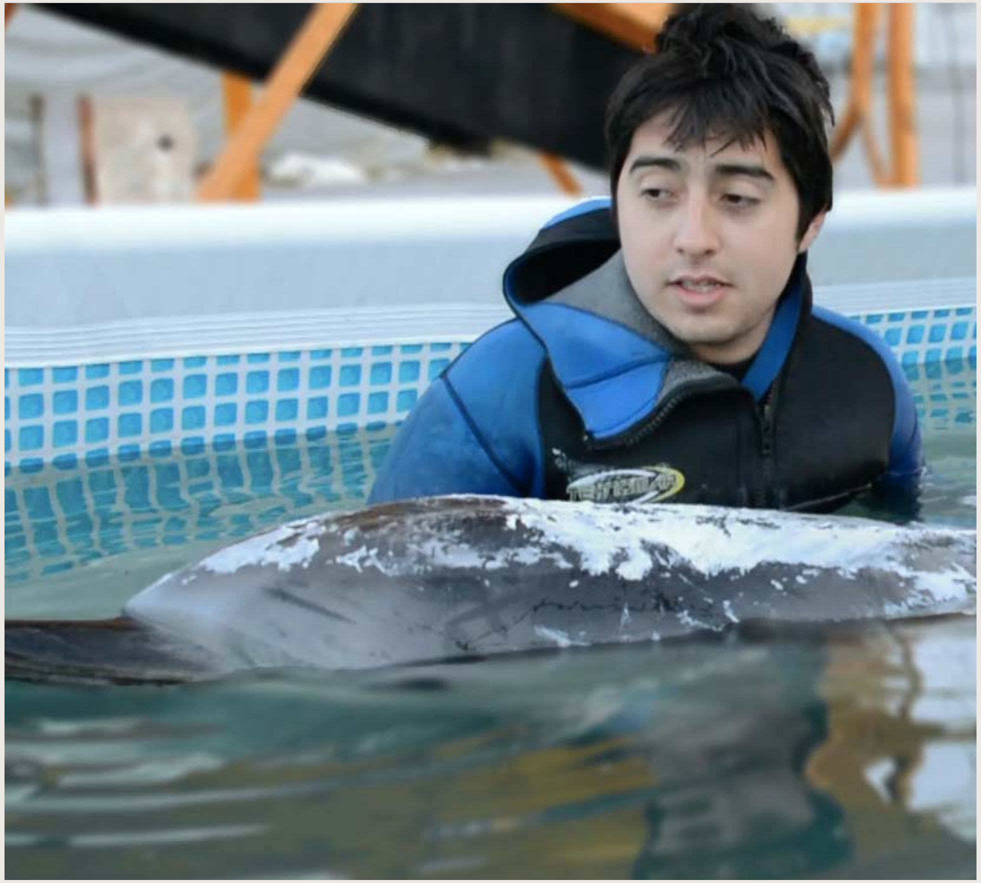

WCA PARTNER ALEXANDER SÁNCHEZ CARES FOR A STRANDED STRIPED DOLPHIN. A MEDICAL POOL IS BEING USED DUE TO THE DOLPHIN BEING WEAK AND UNABLE TO COPE WITH ROUGH SEA CONDITIONS. THE DEVELOPMENT OF SEASIDE SANCTUARIES WOULD PROVIDE A VASTLY IMPROVED ENVIRONMENT FOR SICK AND INJURED CETACEANS DURING RESCUE AND REHABILITATION. © ALEXANDER SÁNCHEZ

7.1. IMPROVING LIVING CONDITIONS FOR CETACEANS IN CAPTIVITY

Because opportunities for relocating captive cetaceans to seaside sanctuaries are currently limited, it is vital that while these individuals remain in captive facilities, they are provided with the highest level of care to ensure the highest possible quality of life.

One method for assessing welfare for captive bottlenose dolphins has been developed by Isabella Clegg and colleagues [40]. The 'C-Well' assessment includes eleven criterion and 36 species-specific measures developed in situ at three marine mammal zoological facilities. The assessment has been tested for feasibility and accuracy, and substantiated by published literature on wild and captive dolphins and veterinary and professional expertise. C-Well scores can be calculated for each measure or combined to achieve an overall score, which allows for the comparison of welfare among individuals and facilities. This work represents a first step in quantifying and systematically measuring welfare among captive cetaceans and can be used as a model for future development in zoos and dolphinariums, as well as a means to support benchmarking, industry best practices, and certification.

One example of the travel industry's efforts to improve standards at dolphinariums comes from the Association of British Travel Agents (ABTA). As part of a set of animal welfare guidance documents for captive and wild tourism activities, ABTA's Animal Welfare Guidelines – Specific Guidance Manual: Dolphins in Captive Environments has provided its members with a set of standards by which to measure the minimum welfare requirements for cetaceans in zoos and aquariums. This guidance document was produced by ABTA in partnership with the Born Free Foundation in 2013, following a multi-stakeholder advisory process. Whilst it is widely acknowledged that ABTA's Animal Welfare

Guidelines require revision and improvement, they have provided a benchmark for the travel industry with which to encourage improvements.

The ABTA Animal Welfare Guidelines can be accessed at:

https://abta.com/abta-shop/abta-animal-welfare-guideline.

ACKNOWLEDGEMENTS

The authors would like to thank Club Med for financially supporting the development of this guide and in particular, Florian Duprat, Corporate Social Responsibility Manager, Club Med, for his support, advice and patience throughout this project. We would also like to extend a special thanks to illustrator Giulia De Amicis and designer Ailsa Marrs for bringing this publication to life! This guide is the result of five years of collaboration with our World Cetacean Alliance (WCA) partners representing experts from the tourism, scientific, and conservation communities, and in particular through the WCAs Responsible Whale and Dolphin Watching Working Group.

This guide has benefitted tremendously from the WCAs collective experience and understanding of the challenges and benefits of whale and dolphin watching worldwide. There exists, of course, a wealth of knowledge outside of the WCA Partnership, and we are very grateful to the many experts that contributed their research, advice and information during several consultation rounds in the lead up to publication. The following list is not exhaustive, and we would like to take this opportunity to apologise for any omissions and thank all those not specifically listed here:

Enzo Aliaga-Rossel PhD., Institute of Ecology/ Universidad Mayor de San Andres; Debs Allbrook, University College Cork; Mariana Batha Alonso, Instituto Boto-Cinza; Pete Allsop, Ilha Blue; Caroline Armon, OnBoard Tours Whales; Andrew Armour, Whale Whisperer; Malcolm Barradell, Pembrokeshire Marine Environmental; Narjisse Ben Moussa, Club Med; Marco Bonato, University of Padova; Aireona Bonnie Raschke; Marcus Bridge; Lynne Burrell; Valcina Candy Stoute, Fisheries Division, Commonwealth of Dominica; Nala Cardillo, WildQuest; Pamela Carzon, G.E.M.M; Josephine Chazot, Souffleurs d'Ecume; Ted Cheeseman, Cheesemans' Ecology Safaris & HappyWhale; Isabella Clegg; Catarina Correia-Fagundes, Madeira Wind Birds; Mel Cosentino, University of Strathclyde; Milly Costa; Sarah Courbis PhD; Anouk de Plaa, Naturalist Guide; Casey De Lange, The Dolphin Centre; Liria del Monte Madrigal, Whale Quest Mx; Kate Dranginis; Ali Druskat; Lloyd Edwards, Raggy Charters; Rocia Espada, Dolphin Adventure Gibraltar; Raphael Fennimore, American Cetacean Society; Luena Fernandes, Humpback Whale Institute; Rita Ferreira, Ventura Nature Emotions; Gene Flipse, Conscious Breath Adventures; Heidrun Frisch-Nwakanma, United Nations Environment Programme/ Convention on the Conservation of Migratory Species of Wild Animals (UNEP/CMS) Secretariat; Anna M Garcia, Antofagasta University, CIFAMAC; Paola Garcia Buron; Shane Gero PhD., Aarhus University; Inês Gomes; Sierra Goodman, Divine Dolphin; Anne Gordon de Barrigon, Whale and Dolphin Wisdom Retreats; Oscar Guzón Zatarain, Onca Explorations; Anouk Ilangakoon, IUCN Cetacean Specialist Group; Lei Lani Stelle, University of Redlands; Catherine Lea, Auckland Whale & Dolphin Safari; Charlotte Leger; Scott Leonard, Marine Mammal Alliance Nantucket; Mette Lubczyk, Naturalist Guide; Torie Mackinnon, Moonraker Dolphin Swims; Ivan Martin, Swiss Cetacean Society; Stephen McCulloch, Protect Wild Dolphins Alliance; Vanessa McKay, Carino Sailing & Dolphin Adventures; Claudia Merill; Patricia O'Sullivan, Chair, World Cetacean Alliance; Chantal Pagel MSc., Auckland University of Technology; Morgane Ratel, Souffleurs d'Ecume; Shavojn Read; Denis Richard, Water Planet; Erick Ross, Fundación MarViva; John Rumney, Eye to Eye Marine Encounters; Siena Schaar, Keiko Conservation; David Schofield, National Oceanic and Atmospheric Administration; Vittoria Sesani; Nic Slocum, Whale Watch West Cork; Amanda Stafford, Dolphin and Whale Connection; Lisa Steiner, Whale Watch Azores; Sharyn Taylor, Melbourn Dolphin; Laura Torre; Wendy van Gool; Heidi van der Watt, Massey University; Courtney Vail, Lightkeepers Foundation; Miranda van der Linde, Whale Watch Azores Futurismo; Elizabeth Zwamborn PhD.

The views and opinions expressed in this report are those of the World Cetacean Alliance and do not necessarily reflect the views or positions of contributors. Additionally, inclusion of specific case studies showing elements of best practice or poor practice should not be interpreted to mean that best practice or poor practice is applicable across all aspects of either the showcased business or the site. The World Cetacean Alliance does not take any responsibility for errors or omissions occurring due to translation of this document into languages from the original version in English.

WE NEED YOUR HELP

- Tell us about any errors, either of omission or of fact, to assist us with improving the next edition of this guide;
- Tell us about your experience of whale and dolphin watching around the world and let us know what you think of this guidance document here:

www.worldcetaceanalliance.org/guidelinesfeedback

REFERENCES

1. O'Connor S, Campbell R, Cortez H, Knowles T. Whale watching worldwide: tourism numbers,expenditures and expanding economic benefits. a special report from the International Fund for Animal Welfare. 2009.
2. Lott R, Williamson C. Cetaceans in Captivity. In: Butterworth A, editor. Marine mammal welfare: human induced change in the marine environment and its impacts on marine mammal welfare. Cham, Switzerland: Springer Nature; 2017.
3. Filby N, Christiansen F, Scarpaci C, Stockin K. Effects of swim-with-dolphin tourism on the behaviour of a threatened species, the Burrunan dolphin Tursiops australis. Endanger Species Res. 2017;32(July):479–90.
4. Bejder L. Linking short and long terms effects of nature based tourism on cetaceans. Dalhousie University; 2005.
5. Stensland E, Berggren P. Behavioural changes in f female Indo-Pacific bottlenose dolphins in response to boat-based tourism. Mar Ecol Prog Ser. 2007;332:225–34.
6. Lundquist D, Sironi M, Würsig B, Rowntree V, Martino J, Lundquist L. Response of southern right whales to simulated swim-with-whale tourism at Península Valdés, Argentina. Mar Mammal Sci. 2013;29(2):1–15.
7. Towers JR, Jones A, Quesnel B, Webber A. Estimated revenue , employment and traffic of NIMMSA member businesses based on a survey conducted in 2012 . 2013;
8. Berggren P, Amir OA, Guissamalo A, Jiddawi NS, Ngazy Z, Stensland E, et al. Sustainable dolphin tourism in East Africa. WIOMSA. Western Indian Ocean Marine Science Association. 2007.
9. Chen CL. From catching to watching: Moving towards quality assurance of whale/dolphin watching tourism in Taiwan. Mar Policy. Elsevier; 2011;35(1):10–7.
10. Kessler M, Harcourt R. Management implications for the changing interactions between people and whales in Ha'apai, Tonga. Mar Policy. Elsevier; 2012;36(2):440–5.
11. Personnal Communications.
12. Wilson C, Tisdell C. Conservation and economic benefits of wildlife-based marine tourism: Sea turtles and whales as case studies. Brisbane; 2002. Report No.: 64.
13. Van Waerebeek K, Baker AN, Félix F, Gedamke J, Iñiguez M, Sanino GP, et al. Vessel collisions with small cetaceans worldwide and with large whales in the Southern Hemisphere, an initial assessment. Lat Am J Aquat Mamm. 2007;6(1):43–69.
14. Parsons ECM. The negative impacts of whale-watching. J Mar Biol. 2012;2012:1–9.
15. Guerra M, Dawson SM, Brough TE, Rayment WJ. Effects of boats on the surface and acoustic behaviour of an endangered population of bottlenose dolphins. Endanger Species Res. 2014;24(3):221–36.
16. Scarpaci C, Bigger SW, Corkeron PJ, Nugegoda D. Bottlenose dolphins (Tursiops truncatus) increase whistling in the presence of" swim-with-dolphin" tour operations. J Cetacean Res Manag. 2000;2(3):183–5.
17. Lusseau D. The short-term behavioral reactions of bottlenose dolphins to interactions with boats in Doubtful Sound, New Zealand. Mar Mammal Sci. 2006;22(4):802–18.
18. Stamation KA, Croft DB, Shaughnessy PD, Waples KA, Briggs S V. Behavioral responses of humpback whales (Megaptera novaeangliae) to whale-watching vessels on the southeastern coast of Australia. Mar Mammal Sci. 2010;26(1):98–122.
19. Meissner AM, Christiansen F, Martinez E, Pawley MDM, Orams MB, Stockin KA. Behavioural effects of tourism on oceanic common dolphins, Delphinus sp., in New Zealand: The effects of markov analysis variations and current tour operator compliance with regulations. PLoS One. 2015;10(1):1–23.
20. Christiansen F, Lusseau D, Stensland E, Berggren P. Effects of tourist boats on the behaviour of Indo-Pacific bottlenose dolphins off the south coast of Zanzibar. Endanger Species Res. 2010;11(1):91–9.
21. Constantine R, Brunton DH, Dennis T. Dolphin-watching tour boats change bottlenose dolphin (Tursiops truncatus) behaviour. Biol Conserv. 2004;117(3):299–307.
22. Lusseau D. The hidden cost of tourism: Detecting long-term effects of tourism using behavioral information. Ecol Soc. 2004;9(1).
23. Currey RJC, Dawson SM, Slooten E, Schneider K, Lusseau D, Boisseau OJ, et al. Survival rates for a declining population of bottlenose dolphins in Doubtful Sound, New Zealand: an information theoretic approach to assessing the role of human impacts. Aquat Conserv Mar Freshw Ecosyst. 2009;19:658–70.
24. Lusseau D, Slooten L, Currey RJC. Unsustainable dolphin-watching tourism in Fiordland, New Zealand. Tour Mar Environ. 2006;3(2):173–8.
25. Bejder L, Samuels A, Whitehead H, Gales N, Mann J, Connor R, et al. Decline in relative abundance of bottlenose dolphins exposed to long-term disturbance. Conserv Biol. 2006;20(6):1791–8.
26. Filby NE, Stockin KA, Scarpaci C. Long-term responses of Burrunan dolphins (Tursiops australis) to swim-with dolphin tourism in Port Phillip Bay, Victoria, Australia: A population at risk. Glob Ecol Conserv. 2014;2:62–71.
27. Martinez E, Orams MB, Stockin KA. Swimmming with an endemic and endangered species: Effects of tourism on Hector's dolphins in Akaroa Harbour, New Zealand. Tour Rev Int. 2011;14(2):99–115.
28. Pérez-Jorge S, Gomes I, Hayes K, Corti G, Louzao M,Genovart M, et al. Effects of nature-based tourism and environmental drivers on the demography of a small dolphin population. Biol Conserv. 2016;197:200–8.
29. Gill JA, Norris K, Sutherland WJ. Why behavioural responses may not reflect the population consequences of human disturbance. Biol Conserv. 2001;97(2):265–8.
30. Lusseau D, Bejder L. The long-term consequences of short-term responses to disturbance experiences from whalewatching impact assessment. Int J Comp Psychol. 2007;20(2).
31. Burgess E. Foraging ecology of common dolphins (Delphinus sp.) in the Hauraki Gulf, New Zealand. Institute of Natural Resources. Massey University; 2006.
32. Stockin KA, Lusseau D, Binedell V, Wiseman N, Orams MB. Tourism affects the behavioural budget of the common dolphin Delphinus sp. in the Hauraki Gulf, New Zealand. Mar Ecol Prog Ser. 2008;355:287–95.
33. Constantine R. Increased avoidance of swimmers by wild bottlenose dolphins (Tursiops truncatus) due to long-term exposure to swim-with-dolphin tourism. 2001;17(4):689–702.
34. Shane SH, Tepley L, Costello L. Life-threatening contact between a woman and a pilot whale captured on film. Mar Mammal Sci. Blackwell Publishing Ltd; 1993 Jul 1;9(3):331–6.
35. Pilgrim B. UNSEEN: Whale breach near miss with swimmer [Video File]. Tonga; 2016.
36. Geraci JR, Ridgway SH. On disease transmission between cetaceans and humans. Mar Mammal Sci. 1991;7(2):191–4.
37. Senigaglia V, Christiansen F, Bejder L, Gendron D, Lundquist D, Noren DP, et al. Meta-analyses of whale-watching impact studies: Comparisons of cetacean responses to disturbance. Mar Ecol Prog Ser. 2016;542(Hoyt 2001):251–63.
38. Filby NE, Stockin KA, Scarpaci C. Social science as a vehicle to improve dolphin-swim tour operation compliance? Mar Policy. Elsevier; 2015;51:40–7.
39. Ballantyne R, Packer J, Hughes K. Tourists' support for conservation messages and sustainable management practices in wildlife tourism experiences. Tour Manag. Elsevier Ltd; 2009;30(5):658–64.
40. Clegg I, Borger-Turner J, Eskelinen H. C-Well: The development of a welfare assessment index for captive bottlenose dolphins (Tursiops truncatus). Anim Welf. 2015;24(3):267–82.

TOGETHER WE ARE WCA

World Cetacean Alliance

- A Whale's Song Expeditions
- AIMM
- Atlantiangra
- Auckland Whale & Dolphin Safari
- Born Free
- Bottazzi
- Cheeseman's Ecology Safaris
- Eye to Eye Marine Encounters
- Dolphin Adventure
- Ionian Dolphin Conservation
- happywhale
- GEMM
- Dolphin Research Center
- CaboTrek
- Campbell River Whale Watching Adventure Tours
- Canadian Marine Environment Protection Society
- CET LAW
- Cetacean Society International
- Cétamada
- Change for Animals Foundation
- Whale Time
- Jean-Michel Cousteau's Ocean Futures Society www.oceanfutures.org
- Azores Whale Watching Futurismo
- CIFAMAC Peninsula Mejillones
- Eagle Eye Adventures Wildlife Tours
- North Island Kayak
- majestic Whale encounters
- Pottwale e.V.
- OCC www.occ.org.uy
- Dolphin & Whale Connection
- The Divine Dolphin Drake Bay, Costa Rica
- Instituto Baleia Jubarte
- Marine Mammal Alliance Nantucket
- Mirissa Water Sports Pioneers of Commercial Whale Watching in Sri Lanka
- Oceano Whale Watching La Gomera
- Knight Inlet Lodge Canada
- Farewell Harbour Lodge
- Individual Partners
- ilha blue
- Farewell Harbour Lodge
- Kingfisher Wilderness Adventures
- Freebird catamarans
- MelbournDolphin
- Marine life
- Love the Oceans
- Wild Side Hawaii
- whale watch Westcork
- North Island Marine Mammal Stewardship Association
- Whalefest
- verballenas.com
- Dana Wharf Whale Watching
- Adventure Quest Tours Canada Inc
- E-Ko New Zealand
- Sodurba Soul of South Durban
- whitetenerife
- Southern Right Charters
- Whale Watch Tenerife
- Life Aquatic
- Project Jonah New Zealand
- Turmares Tarifa
- Elding The Whale Watching Pioneers
- Wild Encounter Thailand
- Raggy Charters
- ROW Sea Kayak Adventures
- Sail Utopia
- SOS Delfines
- Stubbs Island Whale Watching
- The Whale & Dolphin Company
- Trinity Eco-Tours Marine Wildlife Adventure Tours
- Whale Quest Los Cabos, Mex.
- Norberto Whale Watch Azores
- Whale Wise Eco Tours
- Cape Ann Whale Watch Gloucester, MA
